# Supplementary material for: A missense mutation in human INSC causes peripheral neuropathy
Source: EMBO Mol Med. 2024 Apr 8;16(5):4. doi: 10.1038/s44321-024-00062-w (PMC11099080; doi:10.1038/s44321-024-00062-w)
Supplement: Supplementary file 1 — Appendix [file 44321_2024_62_MOESM1_ESM.pdf]

Appendix

A missense mutation in human *INSC* causes peripheral neuropathy

Table of Contents

Appendix Fig S1. Clinical pictures of the patients with INSC p.Met70Arg. 2

Appendix Fig S2. Multipoint parametric linkage analysis of CMT2 in the family. 3

Appendix Fig S3. *hINSC* shRNA knockdown SH-SY5Y cell and *Baz/dInsc/Pins* knockdown fly validation. 4

Appendix Fig S4. *hINSC* transgenic fly mRNA and protein level validations. 5

Appendix Fig S5. Schematic of a seamless editing knock-in strategy and sequence alignment of LGN binding domain. 6

Appendix Fig S6. The expression pattern and protein localization of *Drosophila Inscuteable* in larval tissues. 7

Appendix Fig S7. The time-lapse imaging of the *Drosophila* walking behaviors. 8

Appendix Fig S8. Feeding assay of Taxol feeding in different doses and genetic background. 9

Appendix Table S1. Nerve conduction studies of the patients carrying *INSC* p.Met70Arg. 10

Appendix Table S2. Bioinformatics analyses and filtering of the whole genome sequencing data. 11

Appendix Table S3. *Drosophila* stocks list. 12

Appendix Table S4. *Drosophila* genotype and associated figures. 14

Appendix Table S5. List of plasmids used in this study. 18

Appendix Table S6. List of primers used in this study. 19

Appendix Table S7. List of antibodies used in this study. 21

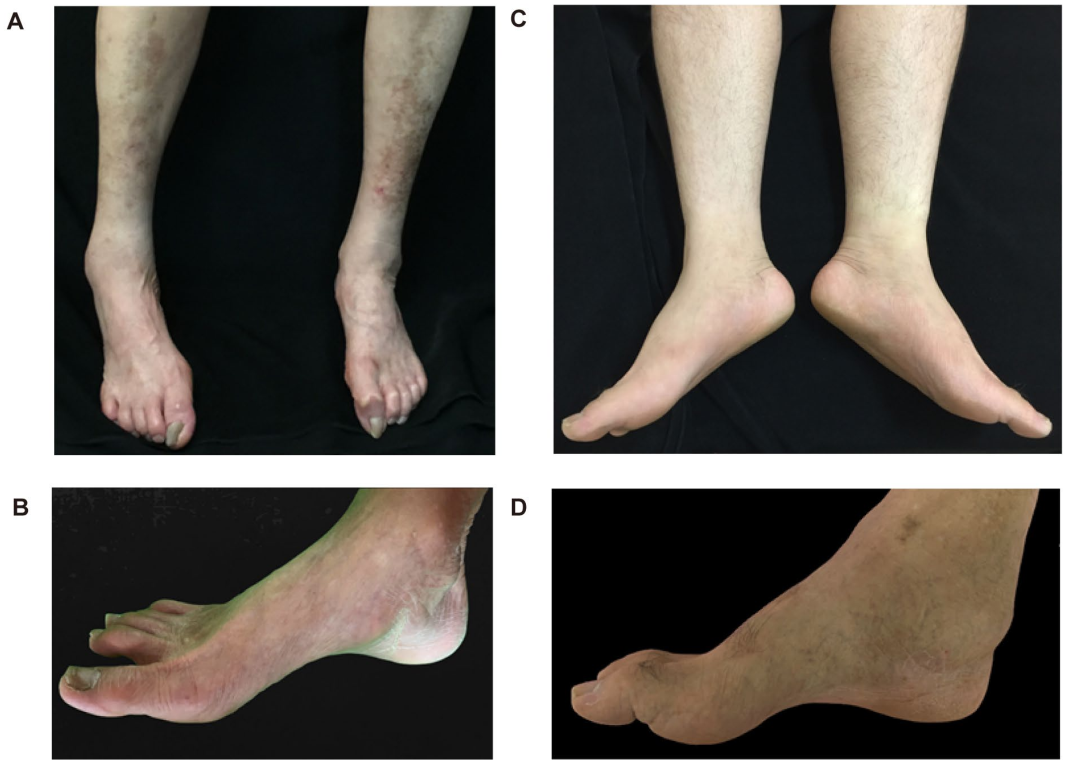

Appendix Figure S1. Clinical pictures of the patients with *INSC* p.Met70Arg.

A. Affected female II-2 aged 59 years, with muscle wasting below the knees.

B. Affected male II-3 aged 57, with pes cavus.

C. Affected male III-3 aged 30, with pes planus.

D. Affected male II-9 aged 49, with pes cavus.

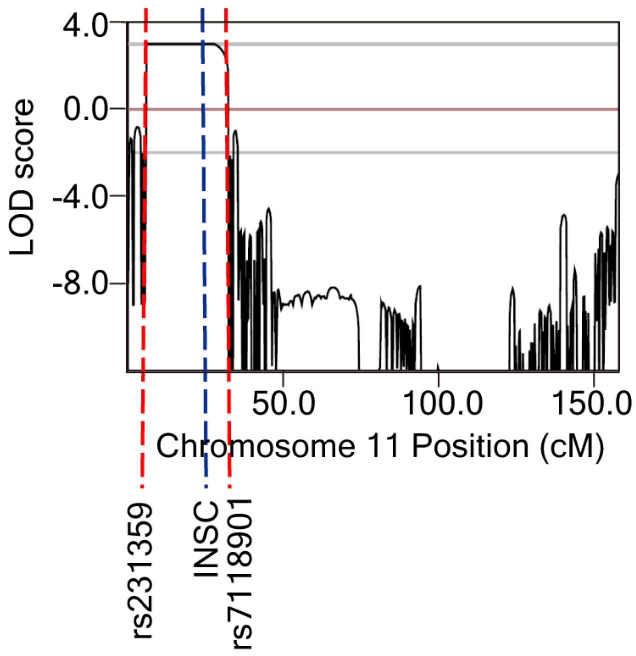

Appendix Figure S2. **Multipoint parametric linkage analysis of CMT2 in the family.** Multipoint parametric linkage analysis revealed three loci with a LOD score of approximately 3, including chr11:2694606-19743250 (GrCh37; Zmax 3.01), chr4:442021-6213622 (Zmax 3), and chr16:72022941-77978326 (Zmax 2.97). The red dotted lines define the flanking markers of the linkage peaks. The blue dotted line shows the location of the *INSC* gene within the chromosome 11 linkage region.

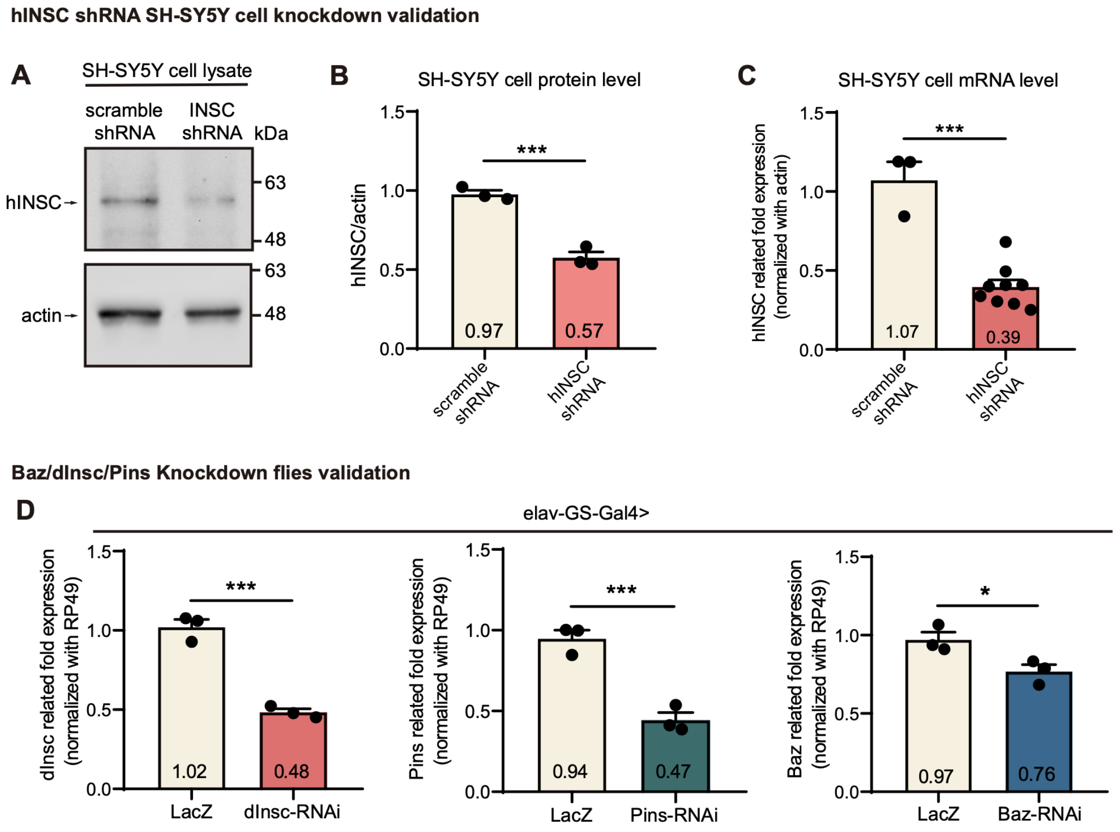

Appendix Fig S3. ***hINSC*** **shRNA knockdown SH-SY5Y cell and** ***Baz/dInsc/ Pins*** **knockdown fly validation**

A. Representative blot from 3 independent western blottings of SH-SY5Y cell extracts of *hINSC*-shRNA and scramble shRNA control.
B. Quantification of (A).
C. Relative *hINSC* mRNA abundance in SH-SY5Y cell extracts of *hINSC*-shRNA and scramble shRNA control.
D. Relative mRNA abundance in whole fly extracts of *dInsc, Pins* and *Baz* RNAi flies driven by *elav-GS*-Gal4; n = 24 flies/genotype from 3 independent fly crosses.

Data information: Error bars indicate mean ± SEM. Statistical analysis was performed using two-tailed Student's t-test. *P < 0.05, **P < 0.01, ***P < 0.001.

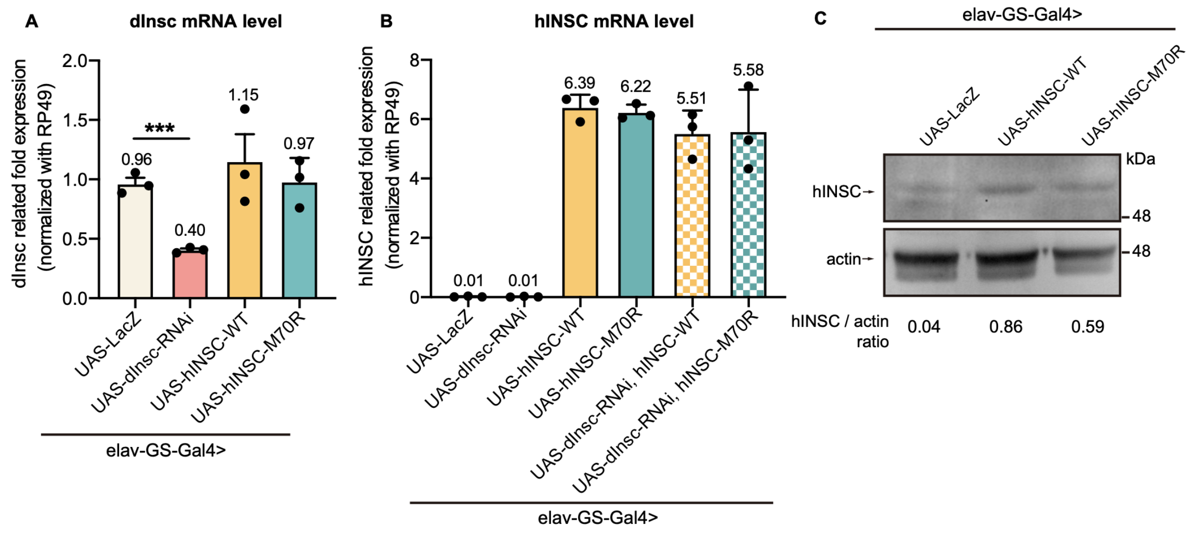

Appendix Fig S4. *hINSC* transgenic fly mRNA and protein level validations

A. Relative *dInsc* mRNA abundance in whole fly extracts of the indicated UAS transgenes driven by *elav-GS*-Gal4; n = 12 flies/genotype from 3 independent fly crosses.
B. Relative *hINSC* mRNA abundance in whole fly extracts of the above indicated groups driven by *elav-GS*-Gal4; n = 12 flies/genotype from 3 independent fly crosses.
C. Representative western blotting of whole fly extracts from UAS-$hINSC^{WT}$ and UAS-$hINSC^{M70R}$, and UAS-*LacZ* control, driven by *elav-GS*-Gal4; n = 5 flies/genotypes.

Data information: Error bars indicate mean ± SEM. Statistical analysis was performed using two-tailed Student's t-test. *P < 0.05, **P < 0.01, ***P < 0.001.

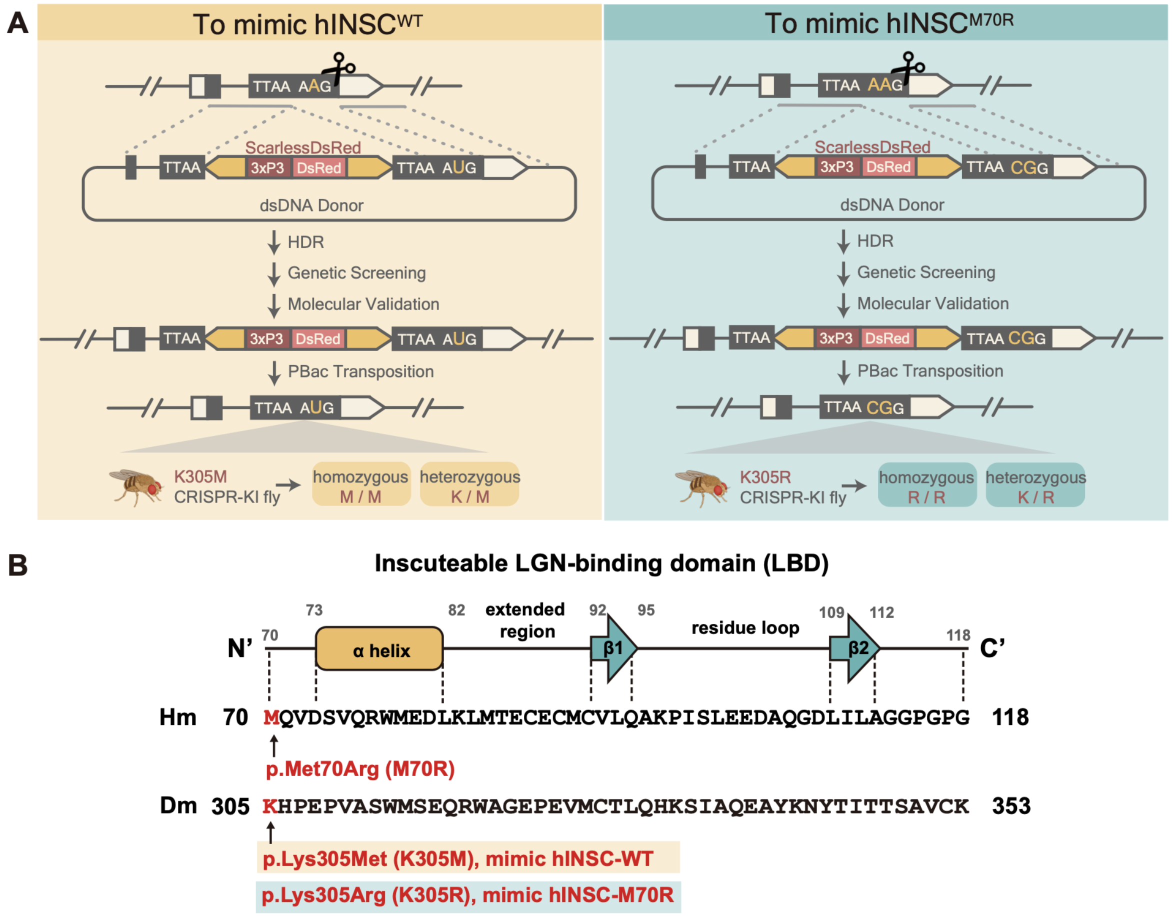

Appendix Fig S5. Schematic of a seamless editing knock-in strategy and sequence alignment of LGN binding domain.

A. Schematic presentation of a seamless editing knock-in strategy to generate two alleles, $dInsc^{K305M}$ and $dInsc^{K305R}$ with CRISPR-Cas9 technique to mimic $hINSC^{WT}$ and $hINSC^{M70R}$, respectively.

B. Sequence alignment of human and flies LGN-binding domain of INSC.

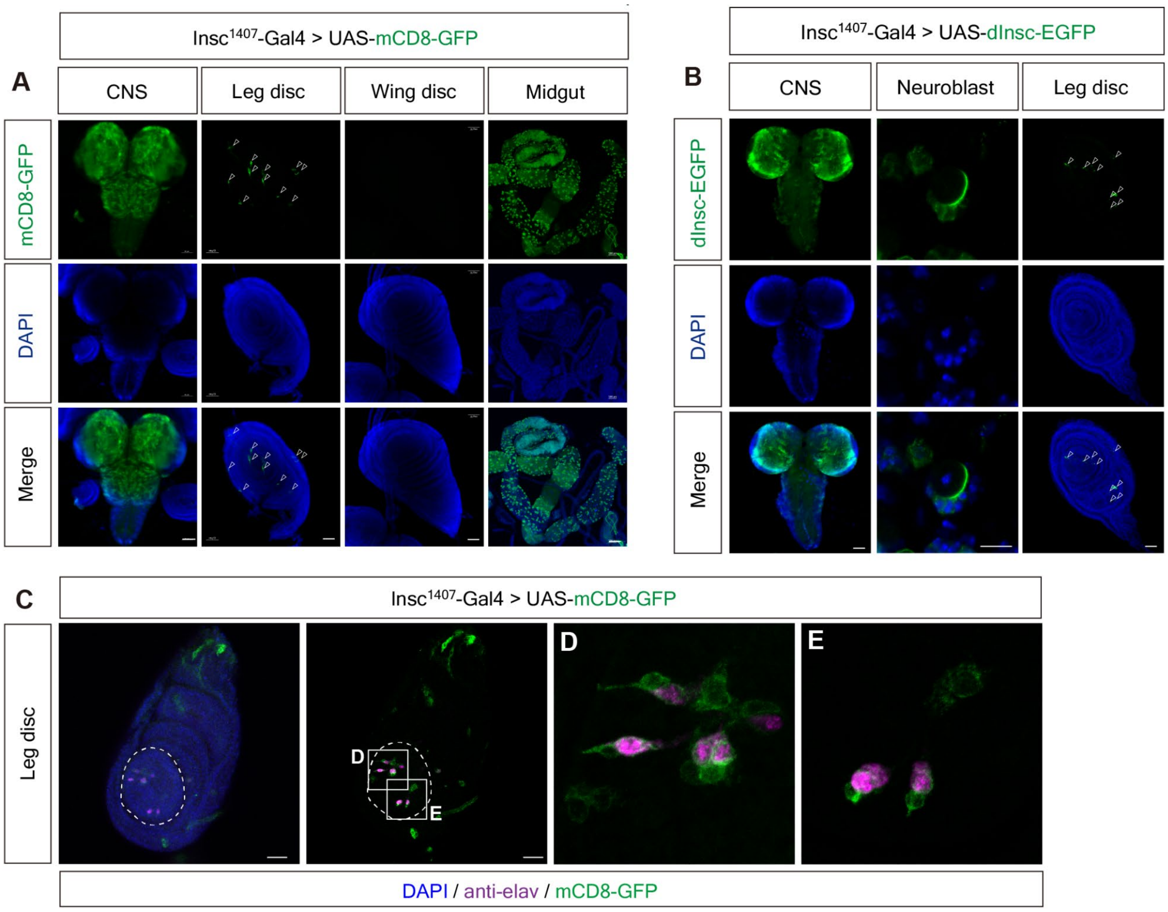

Appendix Fig S6. The expression pattern and protein localization of *Drosophila Inscuteable* in larval tissues.

A. Representative images of CNS, leg disc, wing disc and midgut labeled with *mCD8-GFP* (green) under the control of *dInsc*^{1407}-Gal4 in third instar larvae. (Blue) DAPI staining. Scale bars: 50, 50, 50 and 100 μm from left to right.

B. Representative confocal images of *dInsc-EGFP* (*dInsc*^{1407}-Gal4>UAS-*dInsc-EGFP*) in CNS, neuroblast, and leg disc of third instar larvae. Green indicates *dInsc*-EGFP. (Blue) DAPI staining. Scale bars: 50, 100 and 50 μm from left to right.

C-E. Representative confocal images of leg disc labeled with *mCD8*-GFP (green) under the control of *dInsc*^{1407}-Gal4 in third instar larvae. (Blue) DAPI staining; (magenta) anti-elav immunofluorescence. Scale bars: 50 μm.

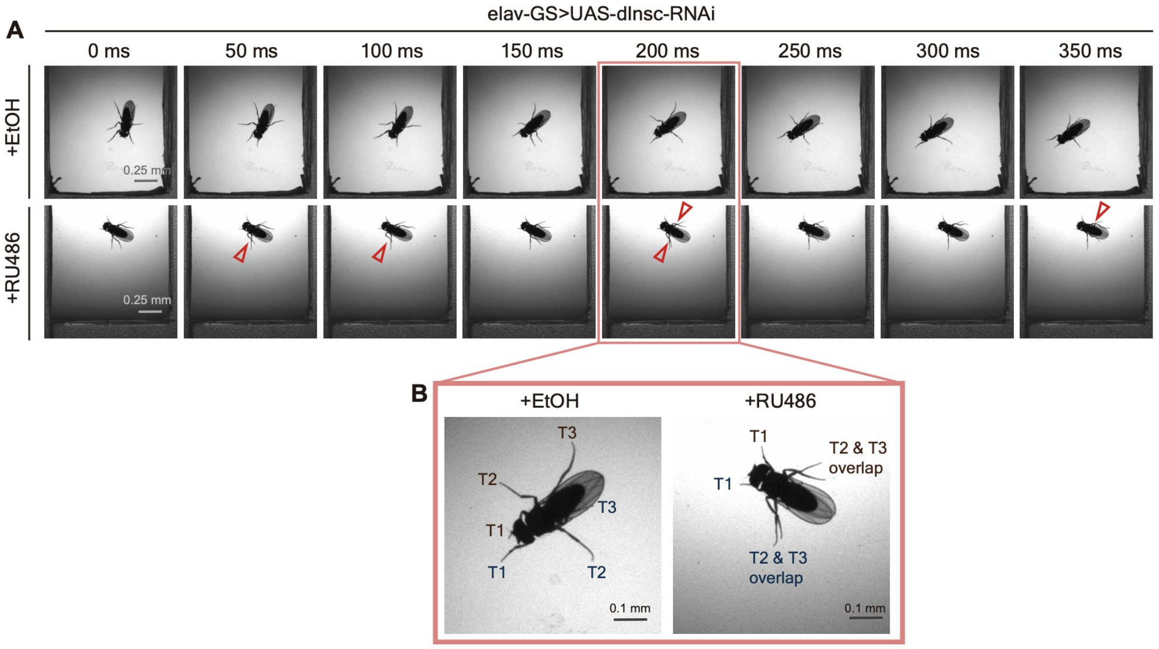

Appendix Fig S7. The time-lapse imaging of the *Drosophila* walking behaviors

A. Snapshots from *Drosophila in vivo* imaging dataset for *elav-GS*>UAS-*dInsc*-RNAi flies fed with RU486 (bottom row), compared with the solvent (EtOH)-fed controls (top row). The interval of time-lapse is 50 ms. Scale bars: 0.25 mm.
B. Magnified images of (A). The RU486-fed flies, but not in solvent-feeding control, showed overlapped T2 and T3 legs (red arrowheads in (A)). Scale bars: 0.1 mm.

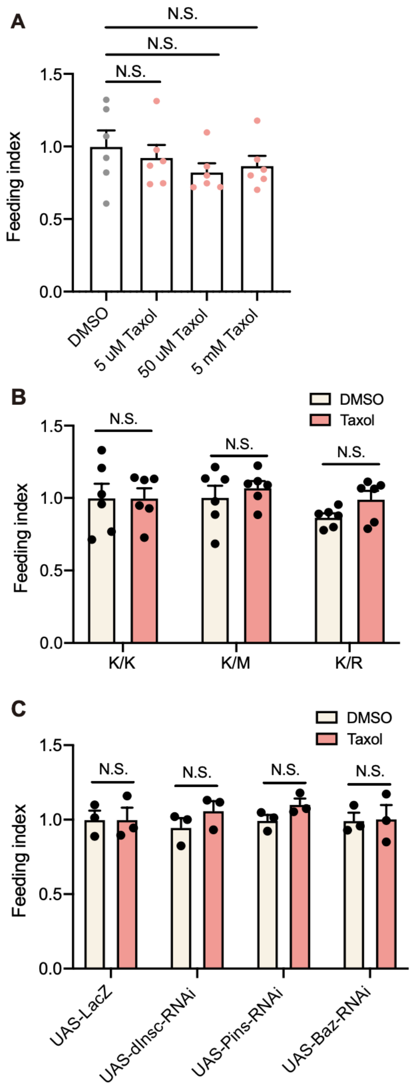

Appendix Fig S8. Feeding assay of Taxol feeding in different doses and genetic background

A. Feeding index of *W1118* (*K/K*) flies fed with different doses (5 µM, 50 µM and 5 mM) of Taxol; 24 flies/genotypes from 6 independent technical replicates.
B. Feeding index of *K/K*, *K/M*, *K/R* flies fed with 50 µM Taxol; 24 flies/genotypes from 6 independent technical replicates.
C. Feeding index of UAS-*LacZ*, UAS-*dInsc*-RNAi, UAS-*Pins*-RNAi and UAS-*Baz*-RNAi flies driven by *elav-GS*-Gal4. The flies were fed with 50 µM Taxol; 12 flies/genotypes from 3 independent technical replicates.

Data information: Error bars indicate mean ± SEM. Statistical analysis was performed using two-tailed Student's t-test. *P < 0.05, **P < 0.01, ***P < 0.001.

Appendix Table S1 Nerve conduction studies of the patients carrying *INSC* p.Met70Arg

Patient	Age at exam (y)	Motor nerve conduction studies						Sensory nerve conduction studies		
		Median nerve		Peroneal nerve		Tibial nerve		Median nerve		Sural nerve
		CV, m/s	CMAP, mV	CV, m/s	CMAP, mV	CV, m/s	CMAP, mV	CV, m/s	SNAP, uV	CV, m/s
II-2	59	53.9	6	37.6	0.3	NR	NR	NR	NR	NR
II-3	57	44.4	4.4	ND	ND	ND	ND	NR	NR	ND
II-8	52	56.6	4.7	45	2.6	42.2	2.0	NR	NR	NR
II-9	49	51.5	7	38	3.9	36.8	4.4	45.2	6	NR
III-3	30	55.5	12.2	39.6	2.1	40.1	1.4	NR	NR	NR
III-7	25	51.3	2.7	43.3	3.0	31.7	1.6	NR	NR	NR
III-8	22	54.5	5	41.7	0.7	36.8	0.7	NR	NR	NR
III-10	21	49	15.6	41.1	1.8	39.3	1	NR	NR	NR
Controls (mean ± SD)		58.6 ± 4	12.0 ± 3.9	48.8 ± 3.9	6.5 ± 2.9	49.6 ± 4.5	14.1 ± 4.5	56 ± 9	37.6 ± 16.4	48.3 ± 10.1

Control values are based on a previously published report.[ref]

CV = conduction velocity; CMAP = compound motor action potential; SNAP = sensory action potential; NR = no response; ND = not done; SD = standard deviation.

Ref: Lin KP, Chan MH, Wu ZA. Nerve conduction studies in healthy Chinese: correlation with age, sex, height and skin temperature. Zhonghua Yi Xue Za Zhi (Taipei) 1993;52:293-297.

Appendix Table S2 Bioinformatics analyses and filtering of the whole genome sequencing data

Filtering criteria	Affected individuals with CMT	
	III-3 (S009)	III-8 (Q217)
Total bases sequenced by whole genome sequencing (Mbases)	118,721	120,758
Average depth in targeted region (x)	37.2	38
Percentage of >10x coverage in targeted region (%)	99.5	99.6
Heterozygous variants	30,671	34,709
Shared heterozygous variants in affected individuals	14824	
Variants not present in 1517 Taiwan biobank control genomes	82	
Variants altering coding sequences	1	
Variants completely segregated with CMT phenotype	1 (*INSC* c.209T>G p.Met70Arg)	

CMT: Charcot-Marie-Tooth disease

Appendix Table S3 *Drosophila* stocks

Name	Genotype	Source
Insc^{1407}-Gal4	*w[*]; P{w[+mW.hs]=GawB}insc[Mz1407]*	BDSC_8751
UAS-dInsc-EGFP	*w[*]; P{UAS-dInsc.EGFP}2*	This study
UAS-mCD8-GFP	*y[1] w[*]; Pin[Yt]/CyO; P{w[+mC]=UAS-mCD8::GFP.L}LL6*	BDSC_5130
elav-GS-Gal4	*y[1] w[*]; P{w[+mC]=elav-Switch.O}GSG301*	BDSC_43642
UAS-dInsc-RNAi	*y[1] v[1]; P{y[+t7.7] v[+t1.8]=TRiP.HMJ22537}attP40/CyO*	BDSC_60358
UAS-w-RNAi	*y[1] v[1]; P{y[+t7.7] v[+t1.8]=TRiP.HMS00017}attP2*	BDSC_33623
UAS-LacZ	*y[1] w[1118]; P{w[+mC]=UAS-lacZ.Exel}3*	BDSC_8530
UAS-Pins-RNAi	*y[1] sc[*] v[1] sev[21]; P{y[+t7.7] v[+t1.8]=TKO.GS00670}attP40*	BDSC_77190
UAS-Bazooka-RNAi	*y[1] v[1]; P{y[+t7.7] v[+t1.8]=TRiP.JF01079}attP2*	BDSC_31523
W1118	*W[1118]*	BDSC_3605
Insc^{InSITE}-Gal4	*w[1118]; PBac{w[+mC]=IT.GAL4}insc[1235-G4] sktl[1235-G4]/CyO*	BDSC_65626
UAS-dInsc-WT	*w[*]; P{w[+mC]=UAS-insc.K}3*	BDSC_39676
Iav-Gal4	*w[*]; P{w[+mC]=iav-GAL4.K}3*	BDSC_52273
OK371-Gal4	*w[1118]; P{w[+mW.hs]=GawB}VGlut[OK371]*	BDSC_26160
Insc^{p49}	*insc[P49]/CyO*	DGRC_108467
UAS-hINSC-WT-FLAG	*w[*]; P{UAS-hINSC-WT.FLAG}3*	This study
UAS-hINSC-M70R-FLAG	*w[*]; P{UAS-hINSC-M70R.FLAG}3*	This study
nan-Gal4	*w[*]; P{w[+mC]=nan-GAL4.K}2*	BDSC_24903
UAS-dPins-mCherry	*w[*]; P{UAS-dPins-mCherry}3*	This study
UAS-hNSC-WT-EGFP	*w[*]; P{UAS-hINSC-WT.EGFP}3*	This study
UAS-hNSC-M70R-EGFP	*w[*]; P{UAS-hINSC-M70R.EGFP}3*	This study
UAS-Bazooka-mCherry	*w[*]; P{y[+t7.7] w[+mC]=UAS-baz::mCherry}attP40/CyO*	BDSC_65844
Insc^{1407}-Gal4, UAS-tub-mCherry	*w[*]; P{w[+mW.hs]=GawB}insc[Mz1407]/CyO; P{w[+mC]=UAS-ChRFP-Tub}3*	BDSC_25773
Elav-Gal4	*P{w[+mC]=GAL4-elav.L}2/CyO*	BDSC_8765
PiggyBac	*w[*]; PBac{GFP[ECFP.3xP3]=5pBlueEye.hsp70-*	BDSC_32174

	PBac\T}Dmel2	
CRISPR dInsc K305M KI	*dInsc-K305M-KI*	This study
CRISPR dInsc K305R KI	*dInsc-K305R-KI*	This study
UAS-MTDS (microtubule-destabilizing system)	*w[*];;UAS-MTDS*	This study

Appendix Table S4 *Drosophila* genotype and associated figures

**Figures**	**Abbreviated genotype**	**Full genotype**
Fig 2A	*elav-GS*-Gal4>UAS-*mCD8-GFP*	*w[*];+/+;P{w[+mC]=elav-Switch.O}GSG301/P{w[+mC]=UAS-mCD8::GFP.L}LL6*
Fig 2A	*elav-GS*-Gal4>UAS-*dInsc*-RNAi	*w[*];P{y[+t7.7] v[+t1.8]=TRiP.HMJ22537}2/+; P{w[+mC]=elav-Switch.O}GSG301*
Fig 2A	*elav-GS*-Gal4>UAS-*w*-RNAi	*w[*];+/+;P{w[+mC]=elav-Switch.O}GSG301/P{y[+t7.7] v[+t1.8]=TRiP.HMS00017}3*
Fig 2A	*elav-GS*-Gal4>UAS-*Pins*-RNAi	*w[*];P{y[+t7.7] v[+t1.8]=TKO.GS00670}2/+; P{w[+mC]=elav-Switch.O}GSG301/+*
Fig 2A	*elav-GS*-Gal4>UAS-*Baz*-RNAi	*w[*];+/+;;P{w[+mC]=elav-Switch.O}GSG301/P{y[+t7.7] v[+t1.8]=TRiP.JF01079}3*
Fig 2B	*dInsc-InSITE*-Gal4> UAS-*dInsc-WT*	*w[*]; PBac{w[+mC]=IT.GAL4}insc[1235-G4] sktl[1235-G4]/+;P{w[+mC]=UAS-insc.K}3/+*
Fig 2B	*dInsc-InSITE*-Gal4 / +	*w[*]; PBac{w[+mC]=IT.GAL4}insc[1235-G4] sktl[1235-G4]/+*
Fig 2B	*dInsc-InSITE*-Gal4> UAS-*hINSC-WT*	*w[*]; PBac{w[+mC]=IT.GAL4}insc[1235-G4] sktl[1235-G4]/+;P{UAS-hINSC-WT.FLAG}3/+*
Fig 2B	*dInsc-InSITE*-Gal4> UAS-*hINSC-M70R*	*w[*]; PBac{w[+mC]=IT.GAL4}insc[1235-G4] sktl[1235-G4]/+;P{UAS-hINSC-M70R.FLAG}3/+*
Fig 2B	*dInsc-InSITE*-Gal4> UAS-*dInsc*-RNAi	*w[*]; PBac{w[+mC]=IT.GAL4}insc[1235-G4] sktl[1235-G4]/P{y[+t7.7] v[+t1.8]=TRiP.HMJ22537}attP40*
Fig 2B	*dInsc-InSITE*-Gal4> UAS-*dInsc*-RNAi, UAS-*hINSC-WT*	*w[*]; PBac{w[+mC]=IT.GAL4}insc[1235-G4] sktl[1235-G4]/P{y[+t7.7] v[+t1.8]=TRiP.HMJ22537}attP40;P{UAS-hINSC-WT.FLAG}3/+*
Fig 2B	*dInsc-InSITE*-Gal4> UAS-*dInsc*-RNAi, UAS-*hINSC-M70R*	*w[illegible]; PBac{w[+mC]=IT.GAL4}insc[1235-G4] sktl[1235-G4]/P{y[+t7.7] v[+t1.8]=TRiP.HMJ22537}attP40;P{UAS-hINSC-M70R.FLAG}3/+*
Fig 2D	*dInsc K305M/+*	w[*];dInsc K305M-KI/+

Fig 2D	*dInsc K305M/dInsc K305M*	w[*];dInsc K305M-KI/dInsc K305M-KI
Fig 2D	*dInsc K305R/+*	w[*];dInsc K305R-KI/+
Fig 2D	*dInsc K305R/dInsc K305R*	w[*];dInsc K305R-KI/dInsc K305R-KI
Fig 2E, Fig EV1A	*insc1407*-Gal4>UAS-*mCD8-GFP*	w[*]; P{w[+mW.hs]=GawB}insc[Mz1407]/+ ; P{w[+mC]=UAS-mCD8::GFP.L}LL6/+
Fig 2I	*Iav*-Gal4 > UAS-*mCD8-GFP*	w[*];+/+; P{w[+mC]=iav-GAL4.K}3/P{w[+mC]=UAS-mCD8::GFP.L}LL6
Fig 2I	*Iav*-Gal4 > UAS-*dInsc*-RNAi	w[*];P{y[+t7.7] v[+t1.8]=TRiP.HMJ22537}2/+; P{w[+mC]=iav-GAL4.K}3/+
Fig 2I	*Iav*-Gal4 > UAS-*Baz*-RNAi	w[*];+/+; P{w[+mC]=iav-GAL4.K}3/P{y[+t7.7] v[+t1.8]=TRiP.JF01079}3
Fig 2I	*Iav*-Gal4 > UAS-*Pins*-RNAi	w[*];+/P{y[+t7.7] v[+t1.8]=TKO.GS00670}2;P{w[+mC]=iav-GAL4.K}3/+
Fig 2J-N	*elav-GS*-Gal4>UAS-*dInsc*-RNAi	w[*];P{y[+t7.7] v[+t1.8]=TRiP.HMJ22537}2/+; P{w[+mC]=elav-Switch.O}GSG301
Fig 3D&E	*nan*-Gal4>UAS-*w*-RNAi	w[*]; P{w[+mC]=nan-GAL4.K}2/+;P{y[+t7.7] v[+t1.8]=TRiP.HMS00017}3/+
Fig 3D&E	*nan*-Gal4>UAS-*dInsc*-RNAi	w[*]; P{w[+mC]=nan-GAL4.K}2/P{y[+t7.7] v[+t1.8]=TRiP.HMJ22537}2;+/+
Fig 3D&E	*nan*-Gal4>UAS-*Pins*-RNAi	w[*]; P{w[+mC]=nan-GAL4.K}2/P{y[+t7.7] v[+t1.8]=TKO.GS00670}2;+/+
Fig 3D&E	*nan*-Gal4>UAS-*Baz*-RNAi	w[*]; P{w[+mC]=nan-GAL4.K}2/+;P{y[+t7.7] v[+t1.8]=TRiP.JF01079}3/+
Fig 3F, Fig EV3A	*dInsc K305M/+*	w[*];dInsc K305M-KI/+
Fig 3F, Fig EV3A	*dInsc K305M/dInsc K305M*	w[*];dInsc K305M-KI/dInsc K305M-KI
Fig 3F	*dInsc K305R/+*	w[*];dInsc K305R-KI/+
Fig 3F, Fig EV3A	*dInsc K305R/dInsc K305R*	w[*];dInsc K305R-KI/dInsc K305R-KI
Fig 3G	*dInsc K305M/+*	w[*];dInsc K305M-KI/+

Fig 3G, Fig EV3B	*dInsc K305M; elav-*Gal4>UAS-*hINSC-WT*	w[*];dInsc K305M-KI/P{w[+mC]=GAL4-elav.L}2;P{UAS-hINSC-WT.FLAG}3/+
Fig 3G, Fig EV2B	*dInsc K305M; elav-*Gal4>UAS-*hINSC-M70R*	w[*];dInsc K305M-KI/P{w[+mC]=GAL4-elav.L}2;P{UAS-hINSC-M70R.FLAG}3/+
Fig 3G, Fig EV2B	*dInsc K305M; elav-*Gal4>UAS-*dInsc-WT*	w[*];dInsc K305M-KI/P{w[+mC]=GAL4-elav.L}2;P{w[+mC]=UAS-insc.K}3/+
Fig 3G, Fig EV3B	*dInsc K305R/+*	w[*];dInsc K305R-KI/+
Fig 3G, Fig EV2B	*dInsc K305R; elav-*Gal4>UAS-*hINSC-WT*	w[*];dInsc K305R-KI/P{w[+mC]=GAL4-elav.L}2;P{UAS-hINSC-WT.FLAG}3/+
Fig 3G, Fig EV2B	*dInsc K305R; elav-*Gal4>UAS-*hINSC-M70R*	w[*];dInsc K305R-KI/P{w[+mC]=GAL4-elav.L}2;P{UAS-hINSC-M70R.FLAG}3/+
Fig 3G, Fig EV2B	*dInsc K305R; elav-*Gal4>UAS-*dInsc-WT*	w[*];dInsc K305R-KI/P{w[+mC]=GAL4-elav.L}2;P{w[+mC]=UAS-insc.K}3/+
Fig 4C-F	*nan-*Gal4>UAS-*hINSC-WT,* UAS-*Pins-mCherry*	w[*]; P{w[+mC]=nan-GAL4.K}2/+;P{UAS-hINSC-WT.FLAG}3/P{UAS-dPins-mCherry}3
Fig 4C-F	*nan-*Gal4>UAS-*hINSC-M70R,* UAS-*Pins-mCherry*	w[*]; P{w[+mC]=nan-GAL4.K}2/+;P{UAS-hINSC-M70R.FLAG}3/P{UAS-dPins-mCherry}3
Fig 4K-L	*iav-*Gal4>UAS-*hINSC-WT,* UAS-*Baz-mCherry*	w[*]; P{w[+mC]=iav-GAL4.K}2/P{y[+t7.7] w[+mC]=UAS-baz::mCherry}2;P{UAS-hINSC-WT.FLAG}3/+
Fig 4K-L	*iav-*Gal4>UAS-*hINSC-M70R,* UAS-*Baz-mCherry*	w[*]; P{w[+mC]=iav-GAL4.K}2/P{y[+t7.7] w[+mC]=UAS-baz::mCherry}2;P{UAS-hINSC-M70R.FLAG}3/+
Fig 5A-C	*Insc1407-*Gal4> UAS-*tubulin-mCherry,* UAS-*w-*RNAi	w[*]; P{w[+mW.hs]=GawB}insc[Mz1407]/+; P{w[+mC]=UAS-ChRFP-Tub}3/P{y[+t7.7] v[+t1.8]=TRiP.HMS00017}3
Fig 5A-C	*Insc1407-*Gal4> UAS-*tubulin-mCherry,* UAS-*dInsc-*RNAi	w[*]; P{w[+mW.hs]=GawB}insc[Mz1407]/P{y[+t7.7] v[+t1.8]=TRiP.HMJ22537}2; P{w[+mC]=UAS-ChRFP-Tub}3/+
Fig 5A-C	*Insc1407-*Gal4> UAS-*tubulin-mCherry,* UAS-*dInsc-*RNAi, UAS-	w[*]; P{w[+mW.hs]=GawB}insc[Mz1407]/P{y[+t7.7] v[+t1.8]=TRiP.HMJ22537}2; P{w[+mC]=UAS-

	hINSC-WT	ChRFP-Tub}3/P{UAS-hINSC-WT.FLAG}3
Fig 5A-C	*Insc1407*-Gal4> UAS-*tubulin-mCherry,* UAS-*dInsc*-RNAi, UAS-*hINSC-M70R*	w[*]; P{w[+mW.hs]=GawB}insc[Mz1407]/P{y[+t7.7] v[+t1.8]=TRiP.HMJ22537}2; P{w[+mC]=UAS-ChRFP-Tub}3/P{UAS-hINSC-M70R.FLAG}3
Fig 5D&E	*W1118*	w[1118]
Fig 5D&E	*dInsc K305M/+*	w[*];dInsc K305M-KI/+
Fig 5D&E	*dInsc K305R/+*	w[*];dInsc K305R-KI/+
Fig 5F&G	*Insc1407*-Gal4> UAS-*tubulin-mCherry,* UAS-*dInsc*-RNAi	w[*]; P{w[+mW.hs]=GawB}insc[Mz1407]/P{y[+t7.7] v[+t1.8]=TRiP.HMJ22537}2; P{w[+mC]=UAS-ChRFP-Tub}3/+
Fig 5F&G	*Insc1407*-Gal4> UAS-*tubulin-mCherry,* UAS-*Baz*-RNAi	w[*]; P{w[+mW.hs]=GawB}insc[Mz1407]/+;P{y[+t7.7] v[+t1.8]=TRiP.JF01079}3/P{w[+mC]=UAS-ChRFP-Tub}3
Fig 5F&G	*Insc1407*-Gal4> UAS-*tubulin-mCherry,* UAS-*Pins*-RNAi	w[*]; P{w[+mW.hs]=GawB}insc[Mz1407]/P{y[+t7.7] v[+t1.8]=TKO.GS00670}2;P{w[+mC]=UAS-ChRFP-Tub}3/+
Fig 6A&D	*dInsc K305M; Insc1407*-Gal4> UAS-*tubulin-mCherry*	w[*];dInsc K305M-KI/P{w[+mW.hs]=GawB}insc[Mz1407];P{w[+mC]=UAS-ChRFP-Tub}3/+
Fig 6A&D	*dInsc K305R; Insc1407*-Gal4> UAS-*tubulin-mCherry*	w[*];dInsc K305R-KI/P{w[+mW.hs]=GawB}insc[Mz1407];P{w[+mC]=UAS-ChRFP-Tub}3/+
Fig 6H, Fig EV5A	*dInsc K305M/+*	w[*];dInsc K305M-KI/+
Fig 6H, Fig EV5A	*dInsc K305R/+*	w[*];dInsc K305R-KI/+
Fig EV4A	*Insc1407*-Gal4> UAS-*tubulin-mCherry*	w[*]; P{w[+mW.hs]=GawB}insc[Mz1407]/+; P{w[+mC]=UAS-ChRFP-Tub}3/+
Fig EV4B	*Insc1407*-Gal4> UAS-*tubulin-mCherry,* UAS-*dInsc*-RNAi	w[*]; P{w[+mW.hs]=GawB}insc[Mz1407]/P{y[+t7.7] v[+t1.8]=TRiP.HMJ22537}2; P{w[+mC]=UAS-ChRFP-Tub}3/+

Appendix Table S5 List of plasmids used in this study

**Name**	**Source**
pUASt-attB-hINSC^{WT}-FLAG	This study (FLAG tag fused to C terminus)
pUASt-attB-UAS-hINSC^{M70R}-FLAG	This study (FLAG tag fused to C terminus)
pUASt-attB-UAS-hINSC^{WT}-EGFP	This study (EGFP fused to C terminus)
pUASt-attB-UAS-hINSC^{M70R}-EGFP	This study (EGFP fused to C terminus)
pUASt-attB-UAS-dInsc-EGFP	This study (EGFP fused to C terminus)
pUASt-attB-UAS-Pins-mCherry	This study (mCherry fused to C terminus)
pFLAG-hINSC^{WT}	This study (FLAG tag fused to C terminus)
pFLAG-hINSC^{M70R}	This study (FLAG tag fused to C terminus)
pMyc-LGN	This study (Myc tag fused to C terminus)
pHA-PAR3	This study (HA tag fused to C terminus)
pBFv-U6.2	Addgene #138400
pScarlessHD-DsRed	Addgene #64703

Appendix Table S6 List of primers used in this study

**Quantitative real-time PCR primers**	
**Gene**	**Primer sequence**
Human INSC	fwd: ACAGTGCTGTCAGCAGGAACTC
	rev: TGAAGGCACCTCTCTGTGACTG
Human beta-Actin	fwd: GCGTCGGTCAATTCAATCTT
	rev: AAGCTGCAACCTCTTCGTCA
Fly Baz	fwd: GACTCGGAGTTGGACTGATGG
	rev: CAGCGTTTGTTACTGCCCTCT
Fly Insc	fwd: GGCGGTTTCTATTCGAGCTTC
	rev: GATGCTGGCTGTCTTTGTGG
Fly Pins	fwd: GCGGAGAATGTGTCCAGTTTG
	rev: GAGTGCGAAGATCCTCGGTTC
Fly RP49	fwd: ACTTCATCCGCCACCAGTCG
	rev: CGGGTGCGCT[illegible]TCGATCC
**Site-Directed Mutagenesis primers**	
**Gene**	**Primer sequence**
hINSC-M70R	fwd: GCGGCTACACCTGAGGCAGGTGGACTCAGTC
	rev: GACTGAGTCCACCTGCCTCAGGTGTAGCCGC
**Molecular cloning primers**	
**Plasmid**	**Primer sequence**
pUASt-attB-UAS-dInsc-EGFP	fwd (BglII site): ATTCGTTAACAGATCTATGTCCTTTCAGCGTAGC<br>rev (XhoI site): TAGAGGTACCCTCGAGTTACTTGTACAGCTCGTCC
pUASt-attB-UAS-dPins-mCherry	fwd (EcoRI site): AGGGAATTGGGAATTCATGTCCTCGCTCTCTGCG
	rev (KpnI site): AAAGATCCTCTAGAGGTACCTTACTTGTACAGCTCGTCCAT

pUASt-attB-UAS-hINSC-WT-FLAG	fwd (EcoRI site): CCCGAATTCCAAAATGAGACGGCCCCCTGGC
	rev (XhoI site): GGGCTCGAGCTACTTGTCATCGTCGTC
pUASt-attB-UAS-hINSC-M70R-FLAG	fwd (EcoRI site): CCCGAATTCCAAAATGAGACGGCCCCCTGGC
	rev (XhoI site): GGGCTCGAGCTACTTGTCATCGTCGTC
pUASt-attB-UAS-hINSC-WT -EGFP	fwd (KpnI site): GTTTTGTGGGATCCGGTACCATGGTGAGCAAGGGCGAG
	rev (KpnI site): AAAGATCCTCTAGAGGTACCTTACTTGTACAGCTCGTCCA
pUASt-attB-UAS-hINSC-M70R -EGFP	fwd (KpnI site): GTTTTGTGGGATCCGGTACCATGGTGAGCAAGGGCGAG
	rev (KpnI site): AAAGATCCTCTAGAGGTACCTTACTTGTACAGCTCGTCCA
pFLAG-hINSC-WT	fwd (EcoRI site): AAATTGAATTCACCATGAGACGGCCCCC
	rev (BamHI site): CGCGGATCCCACAAAACTCTCCTCC
pFLAG-hINSC-M70R	fwd (EcoRI site): AAATTGAATTCACCATGAGACGGCCCCC
	rev (BamHI site): CGCGGATCCCACAAAACTCTCCTCC

Appendix Table S7 List of antibodies used in this study

**Name**	**Source**	**Identifier**	**concentration**
Rat anti-FLAG	Novus biologicals	Novus Cat# NBP1-06712; RRID:AB_1625981	1:500 (IF), 1:1000 (WB)
Mouse anti-DLG	DSHB	DSHB Cat# 4F3	1:200 (IF)
Rabbit anti-HA	Abcam	Abcam Cat# ab9110; RRID:AB_307019	1:500 (IF), 1:1000 (WB)
Mouse anti-c-Myc	DSHB	DSHB Cat# 9E10; RRID:AB_2266850	1:500 (IF), 1:1000 (WB)
Rat anti-alpha-tubulin	Abcam	Abcam Cat# ab6160; RRID:AB_305328	1:500 (IF)
Mouse anti-acetylated tubulin	Sigma-Aldrich	Sigma-Aldrich Cat# T6793; RRID:AB_477585	1:500 (IF)
Rabbit anti-*Drosophila* Insc antibody	A gift from Dr. Hongyan Wang, Duke-NUS medical school	-	1:1000 (WB)
Rabbit anti-human INSC antibody	Invitrogen	Thermo Fisher Scientific Cat#PA5-58921	1:1000 (WB)
Mouse anti-actin	Biolegend	Biolegend Cat#643807	1:10000 (WB)
Alexa Fluor@-568 Phalloidin	Invitrogen	Thermo Fisher Scientific Cat#A12380	1:500
Alexa Fluor@-488 anti-Mouse IgG	Jackson ImmunoResearc h Laboratories	Jackson ImmunoResearch Labs Cat# 715-545-150; RRID:AB_2340846	1:500
