# Supplementary figures and images for: A missense mutation in human INSC causes peripheral neuropathy
Source: EMBO Mol Med. 2024 Apr 8;16(5):4. doi: 10.1038/s44321-024-00062-w (PMC11099080; doi:10.1038/s44321-024-00062-w)

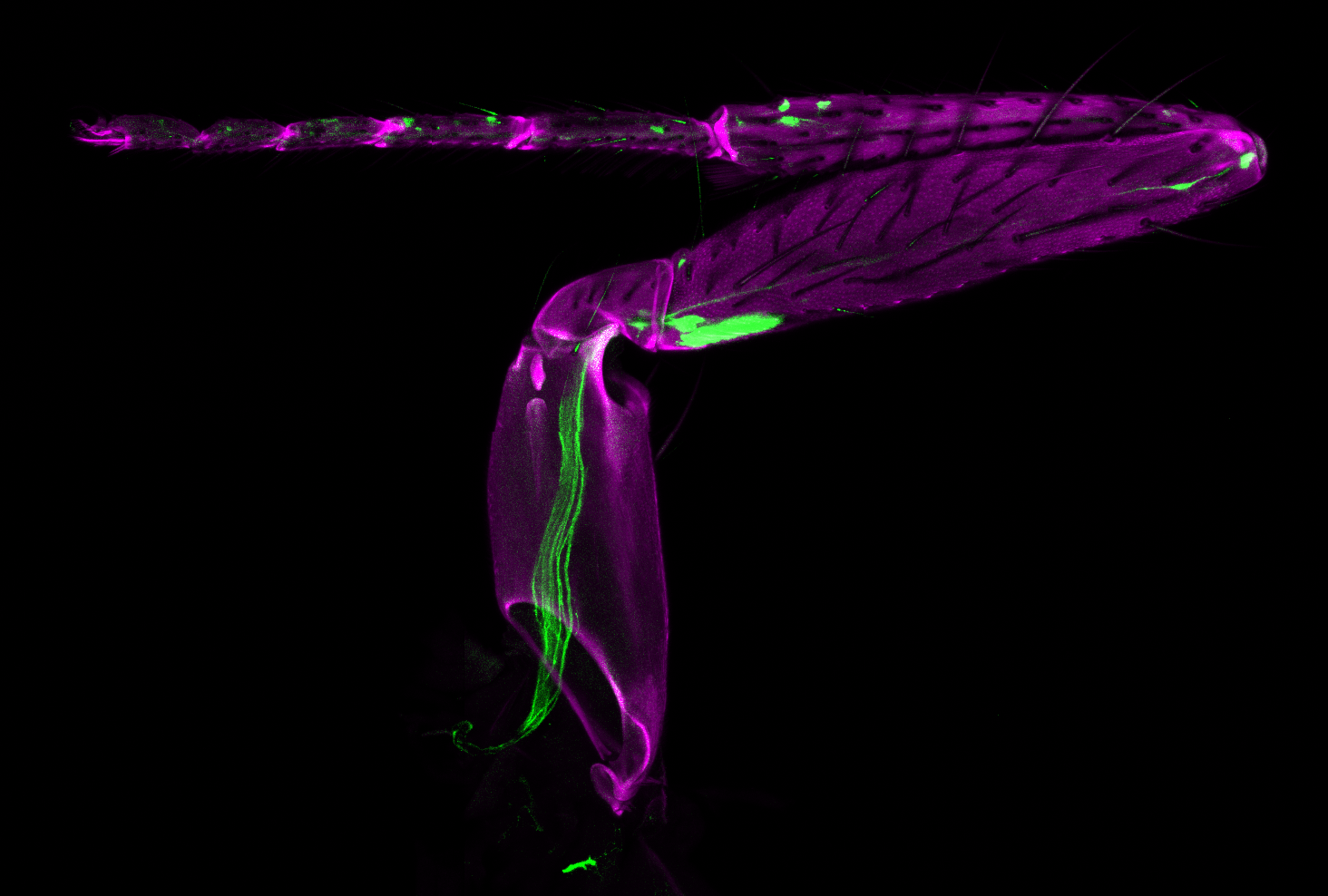

Supplement: Supplementary file 5 — Source data Fig. 2 [file 44321_2024_62_MOESM5_ESM.zip › Figure 2/Fig2E.tif]

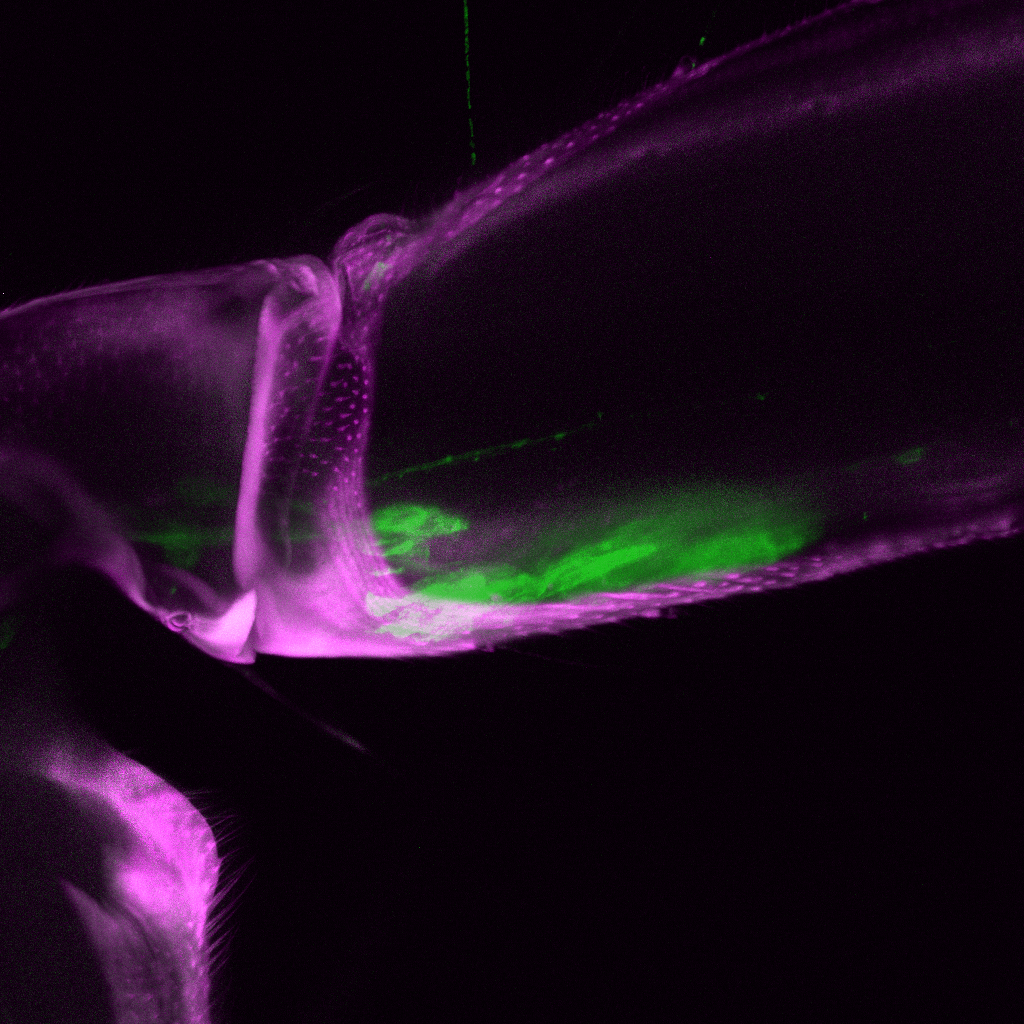

Supplement: Supplementary file 5 — Source data Fig. 2 [file 44321_2024_62_MOESM5_ESM.zip › Figure 2/Fig2F.tif]

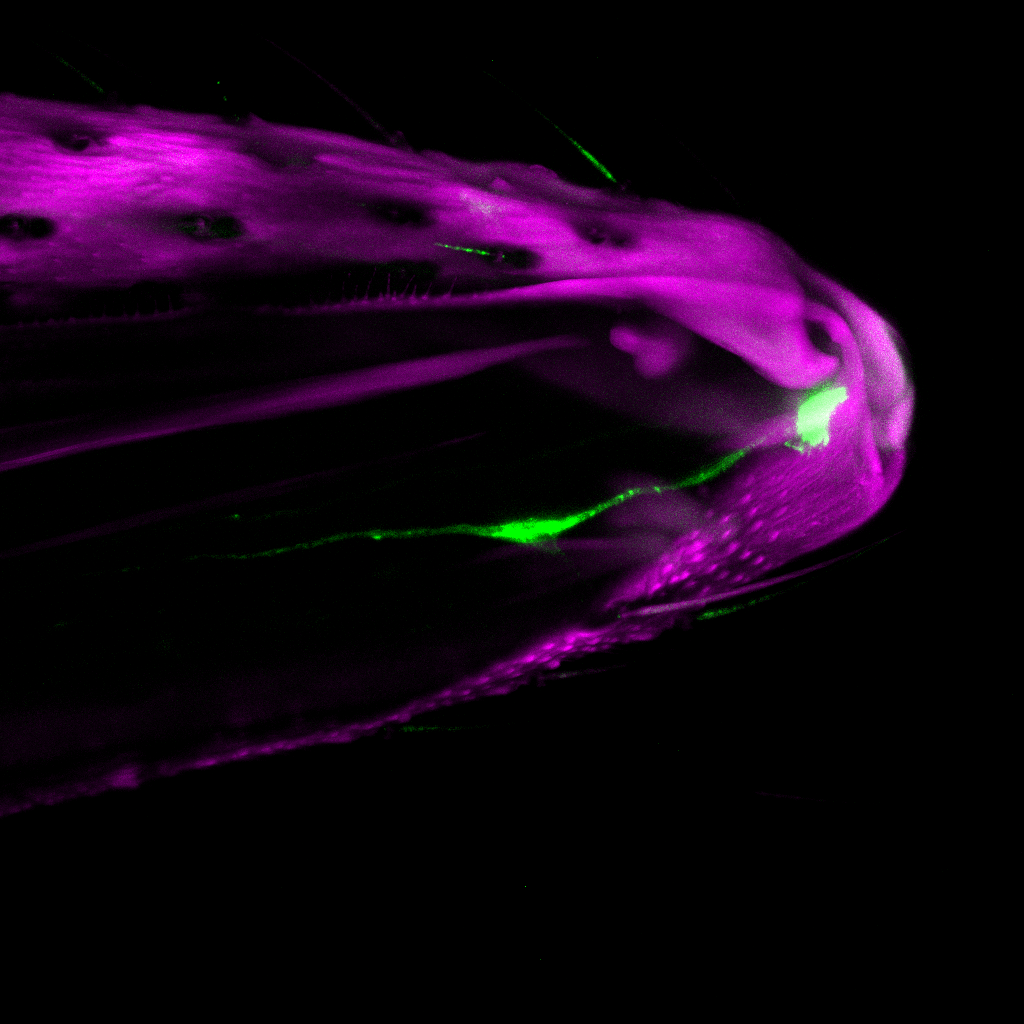

Supplement: Supplementary file 5 — Source data Fig. 2 [file 44321_2024_62_MOESM5_ESM.zip › Figure 2/Fig2G.tif]

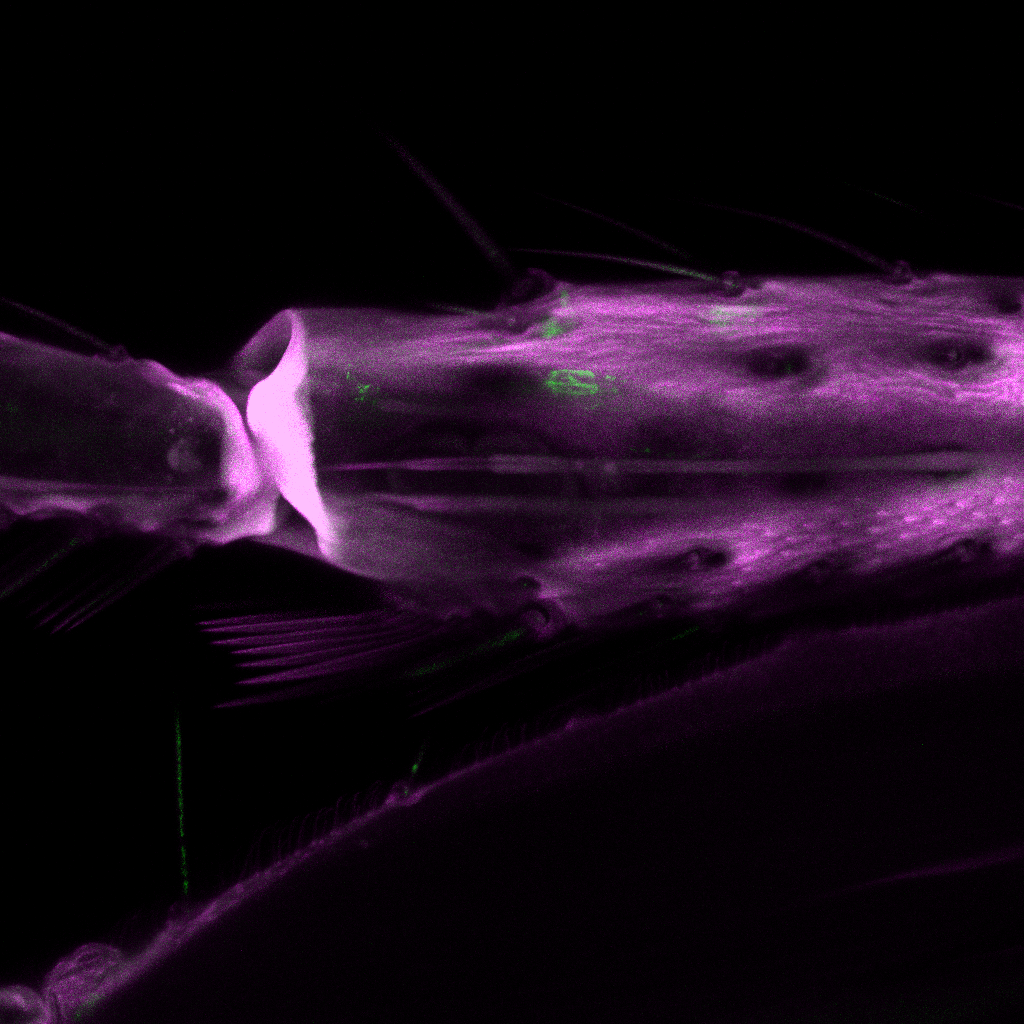

Supplement: Supplementary file 5 — Source data Fig. 2 [file 44321_2024_62_MOESM5_ESM.zip › Figure 2/Fig2H.tif]

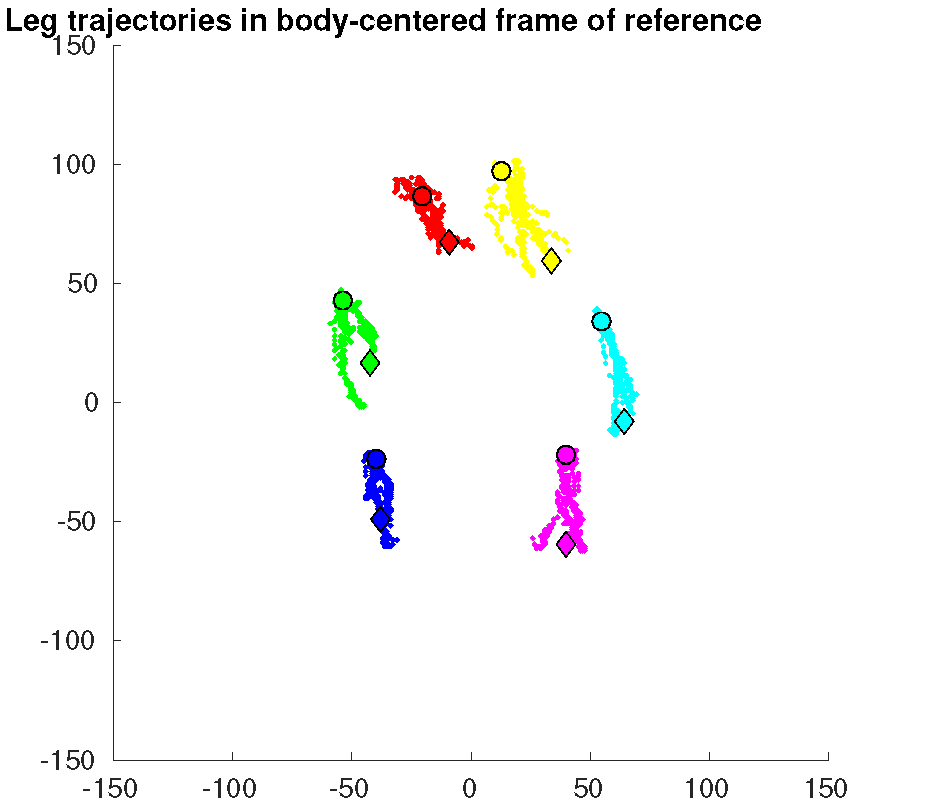

Supplement: Supplementary file 5 — Source data Fig. 2 [file 44321_2024_62_MOESM5_ESM.zip › Figure 2/Fig2J/Fig2J-EtOH.tiff]

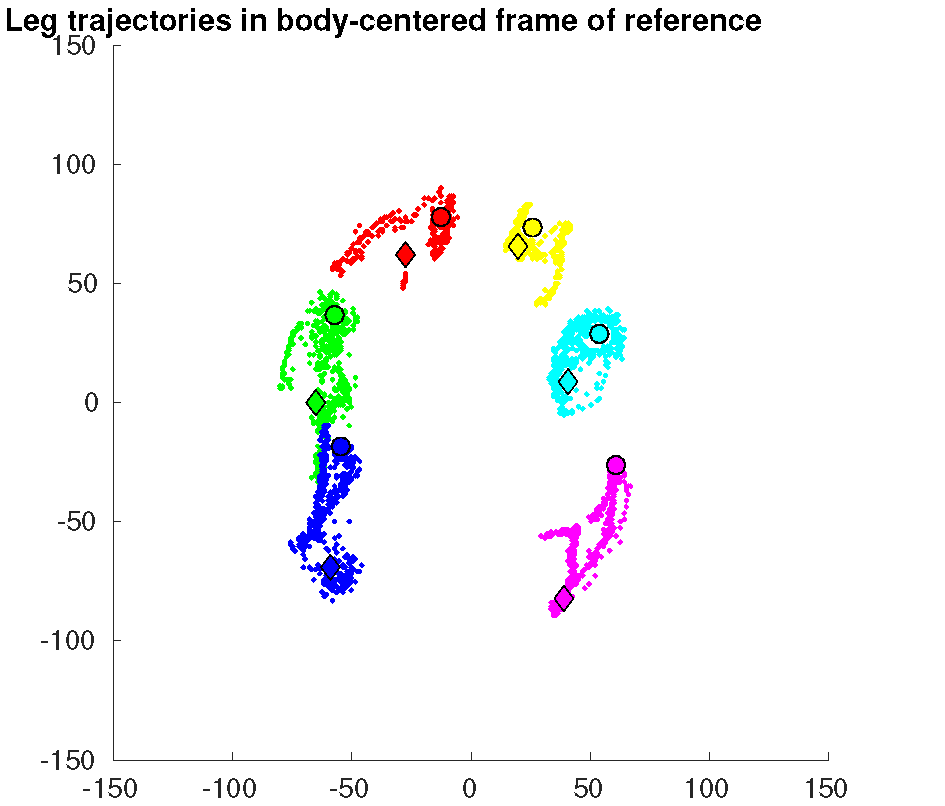

Supplement: Supplementary file 5 — Source data Fig. 2 [file 44321_2024_62_MOESM5_ESM.zip › Figure 2/Fig2J/Fig2J-RU486.tif]

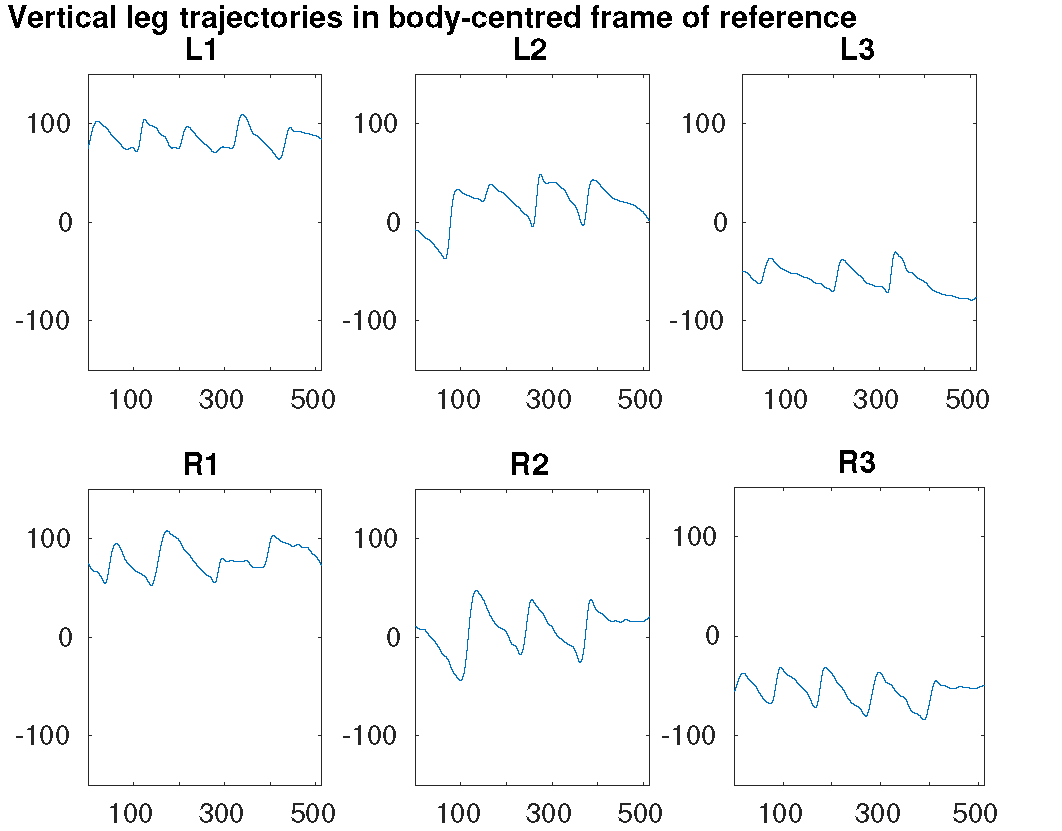

Supplement: Supplementary file 5 — Source data Fig. 2 [file 44321_2024_62_MOESM5_ESM.zip › Figure 2/Fig2K/Fig2K-EtOH.tif]

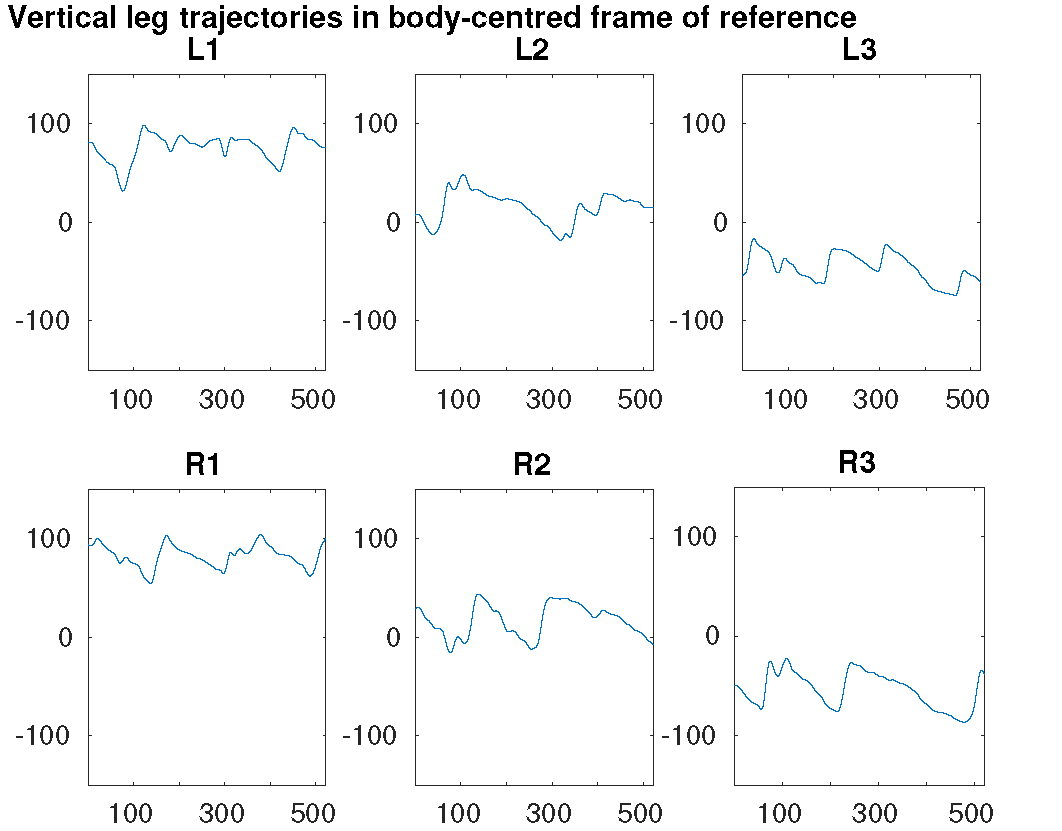

Supplement: Supplementary file 5 — Source data Fig. 2 [file 44321_2024_62_MOESM5_ESM.zip › Figure 2/Fig2K/Fig2K-RU486.tif]

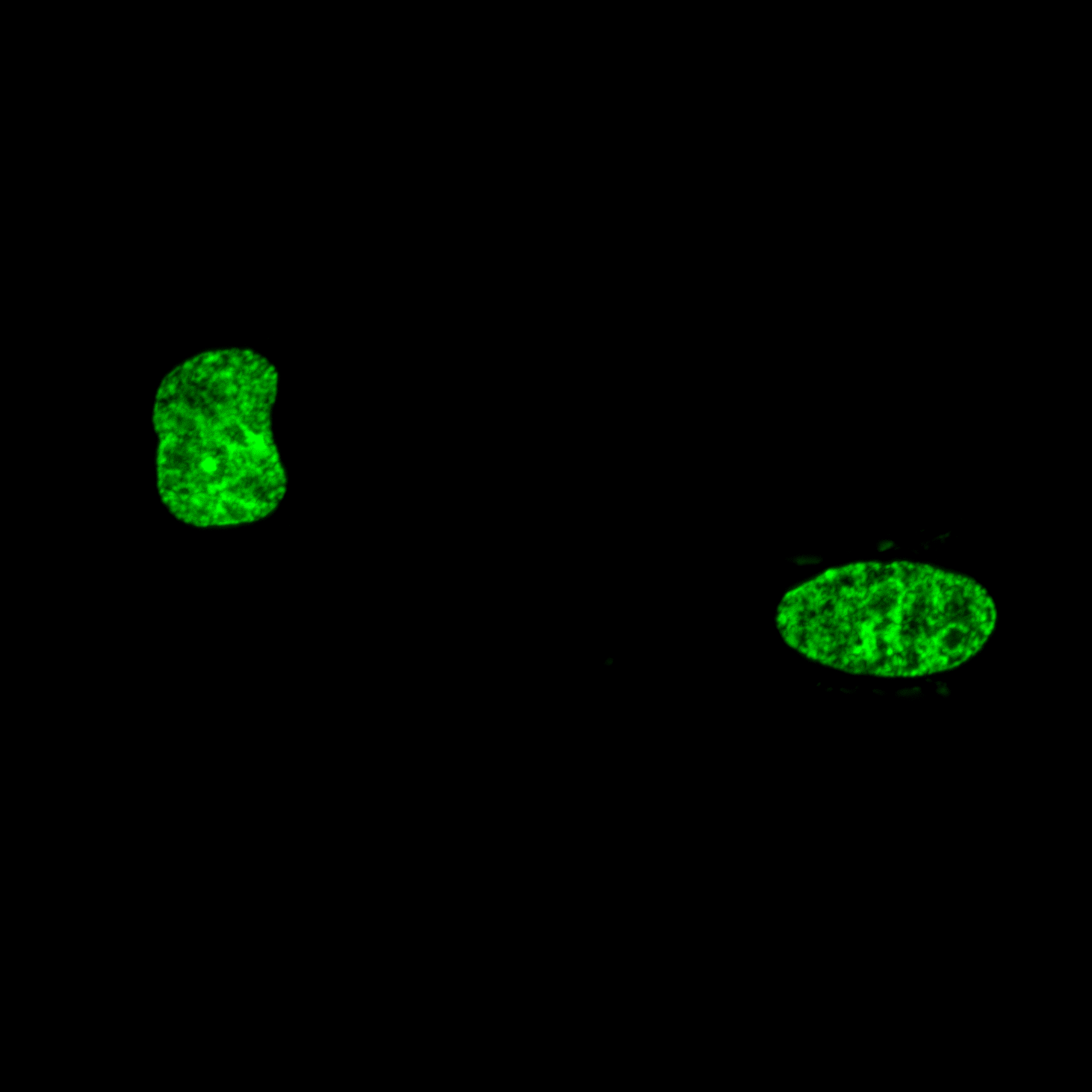

Supplement: Supplementary file 6 — Source data Fig. 3 [file 44321_2024_62_MOESM6_ESM.zip › Figure 3/Fig3B/Fig3B-INSC shRNA-1-DAPI.tif]

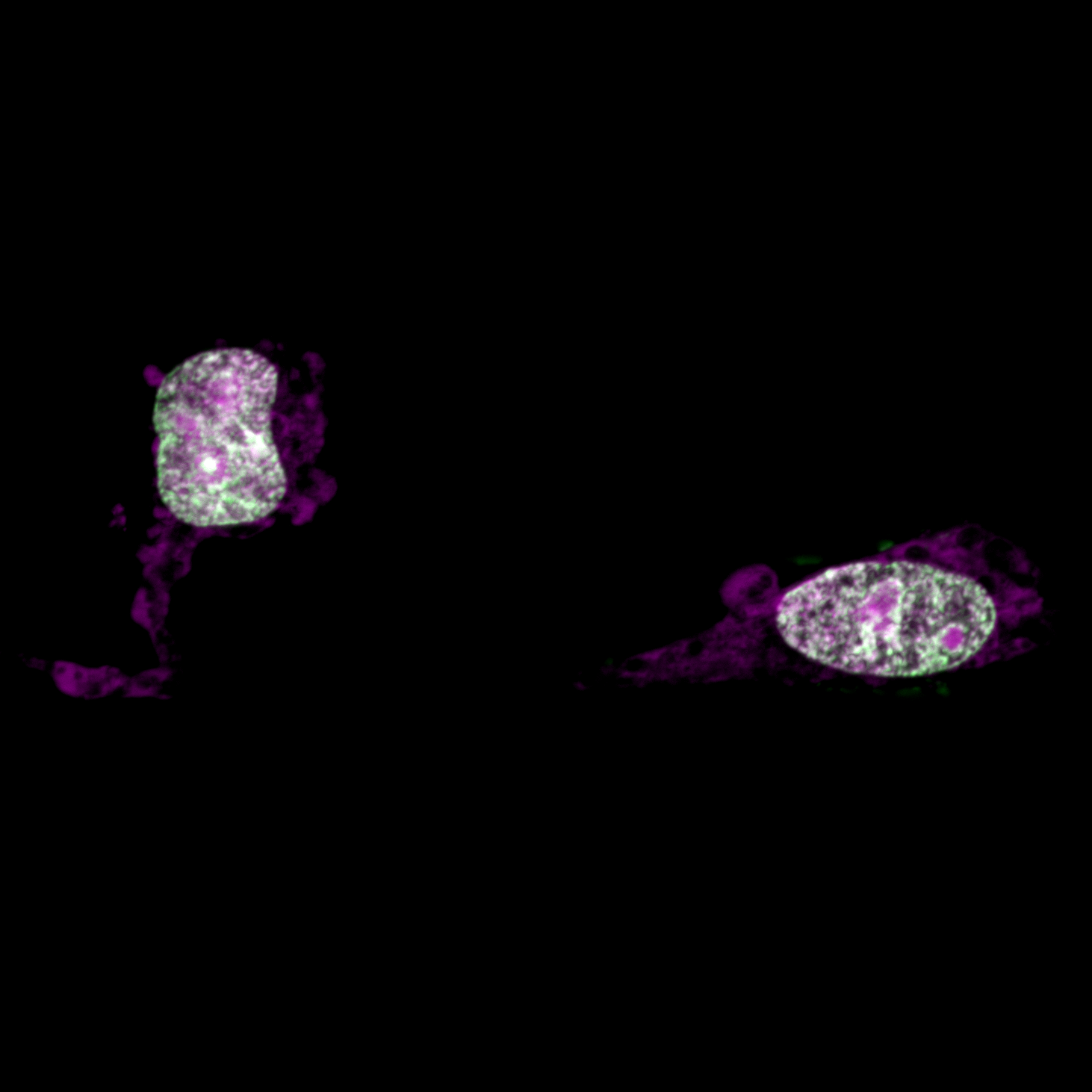

Supplement: Supplementary file 6 — Source data Fig. 3 [file 44321_2024_62_MOESM6_ESM.zip › Figure 3/Fig3B/Fig3B-INSC shRNA-1-Merge.tif]

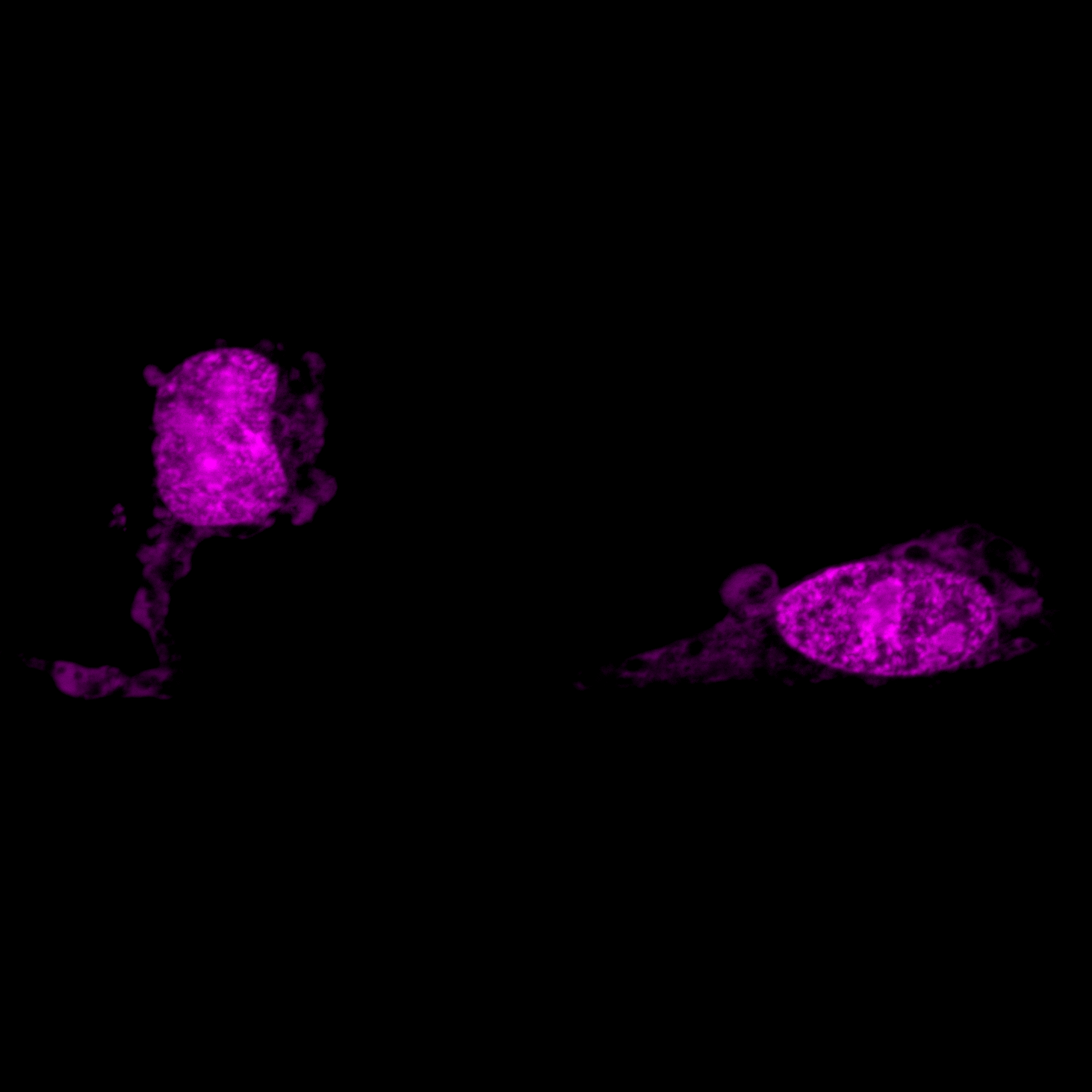

Supplement: Supplementary file 6 — Source data Fig. 3 [file 44321_2024_62_MOESM6_ESM.zip › Figure 3/Fig3B/Fig3B-INSC shRNA-1-PI.tif]

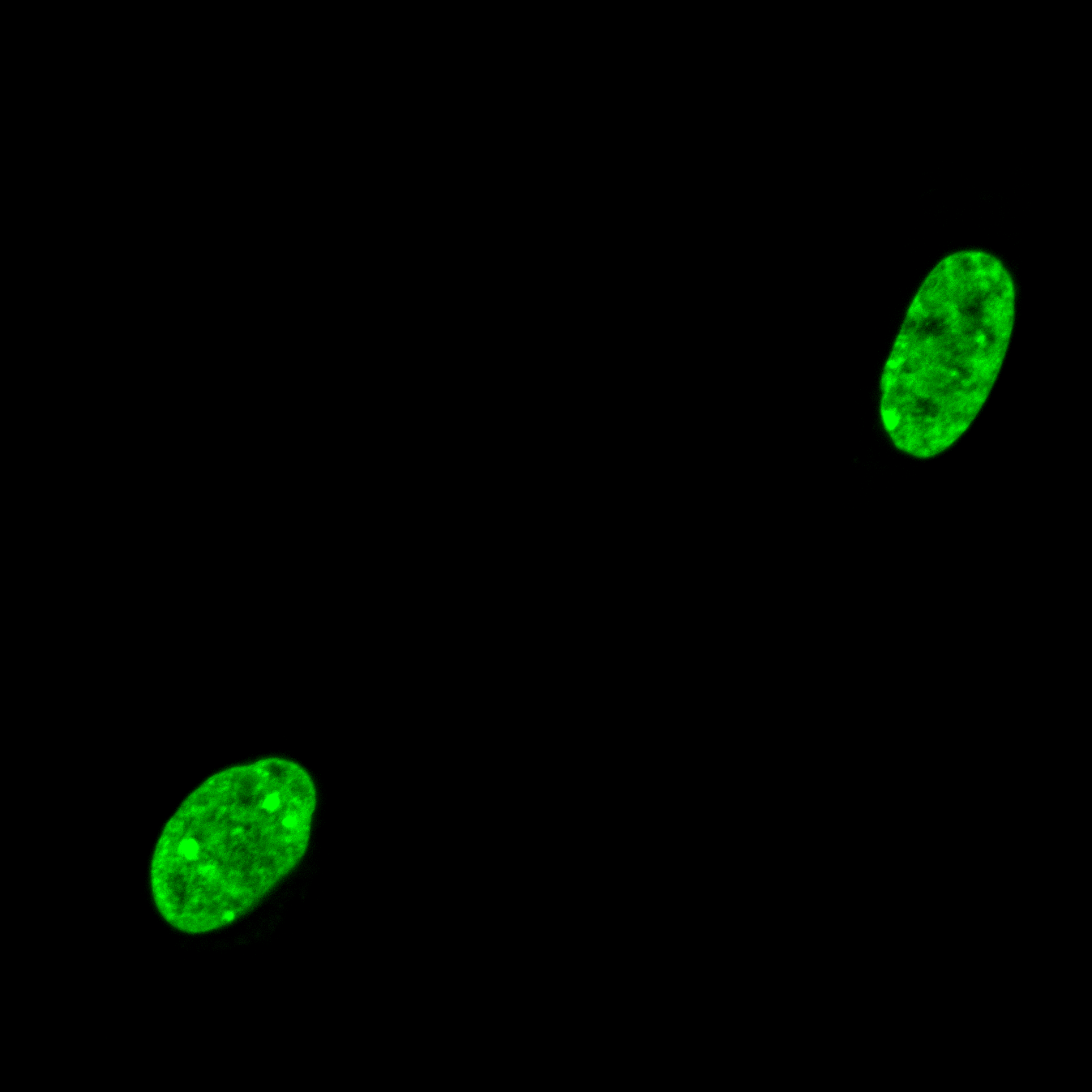

Supplement: Supplementary file 6 — Source data Fig. 3 [file 44321_2024_62_MOESM6_ESM.zip › Figure 3/Fig3B/Fig3B-INSC shRNA-2-DAPI.tif]

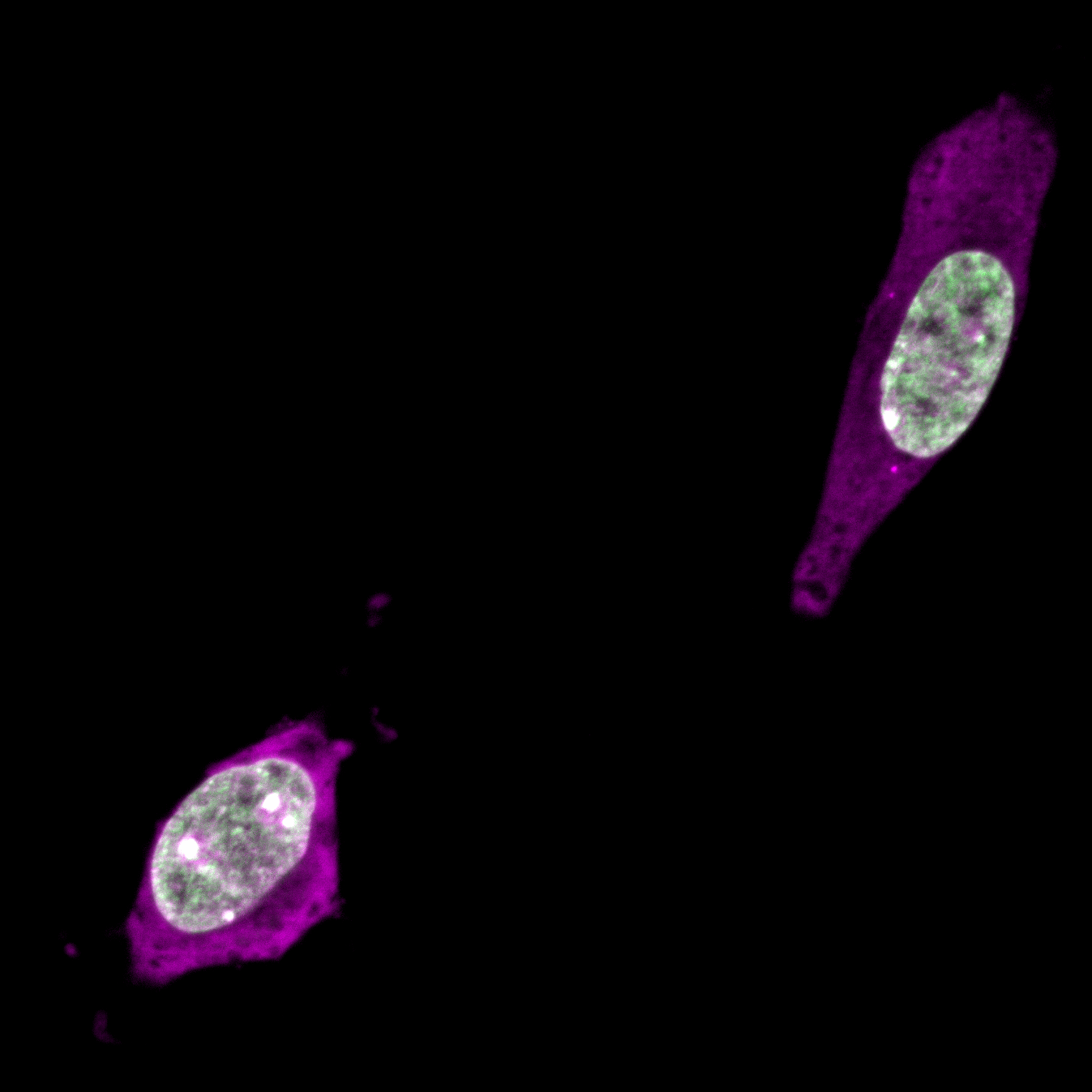

Supplement: Supplementary file 6 — Source data Fig. 3 [file 44321_2024_62_MOESM6_ESM.zip › Figure 3/Fig3B/Fig3B-INSC shRNA-2-Merge.tif]

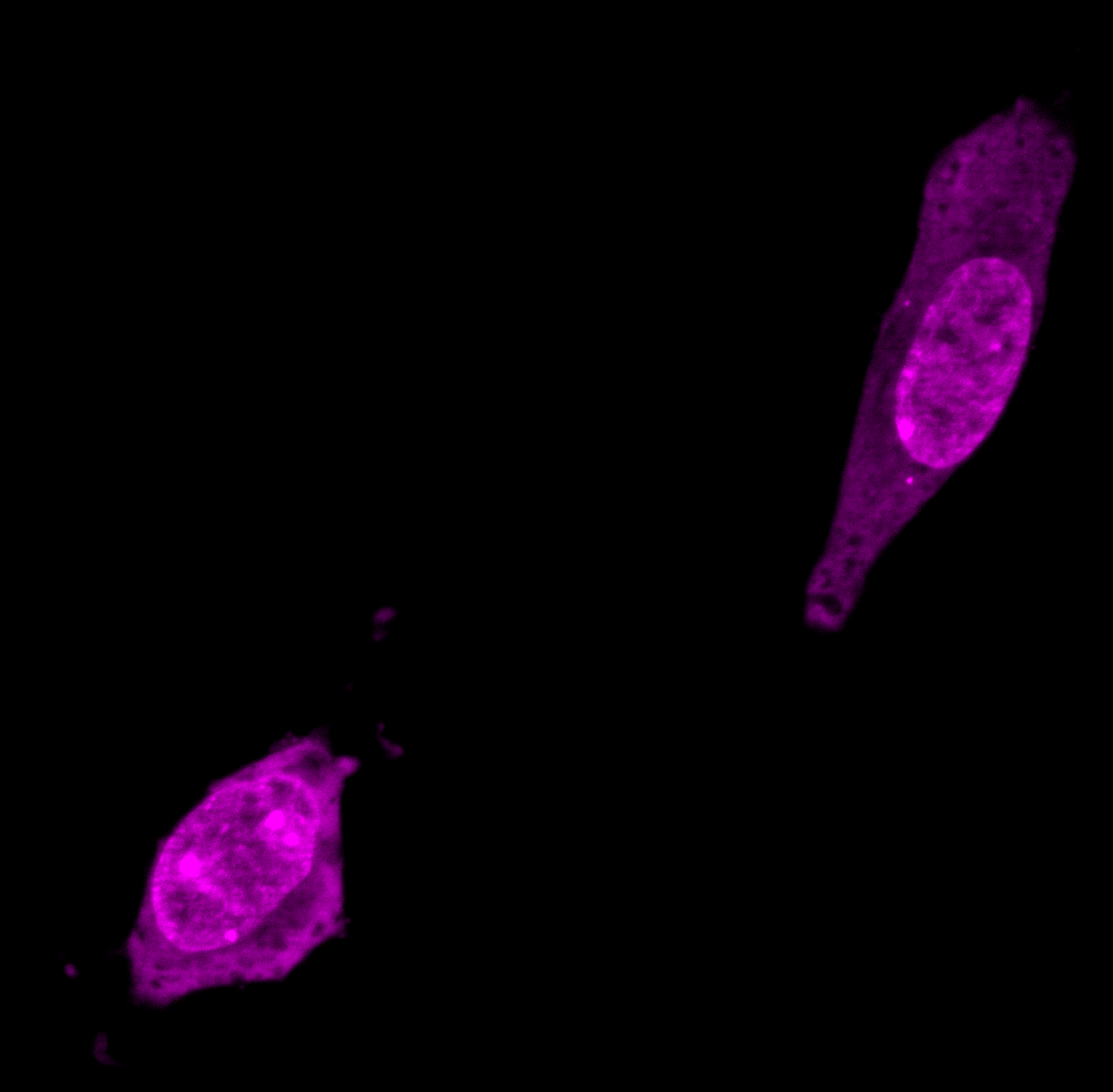

Supplement: Supplementary file 6 — Source data Fig. 3 [file 44321_2024_62_MOESM6_ESM.zip › Figure 3/Fig3B/Fig3B-INSC shRNA-2-PI.tif]

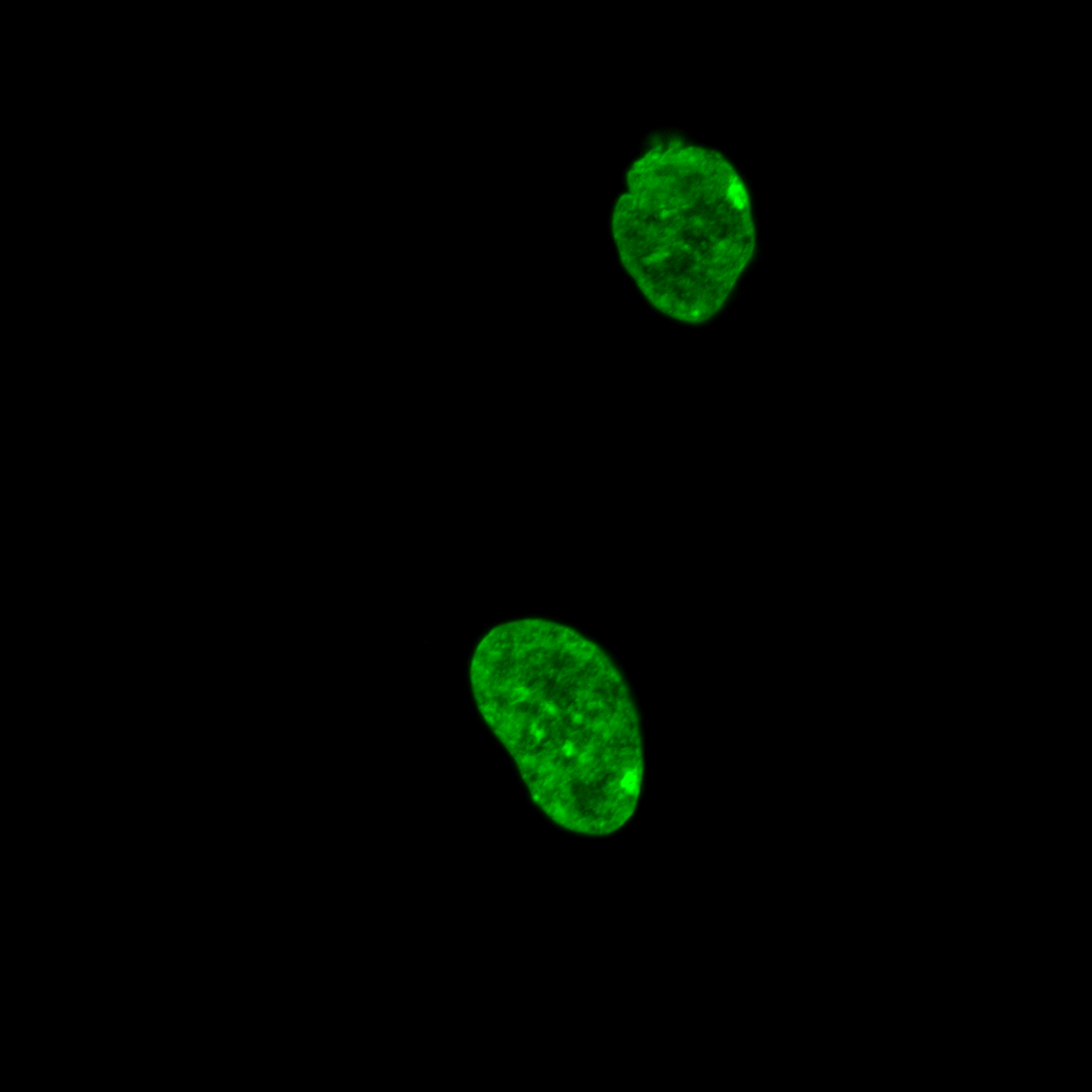

Supplement: Supplementary file 6 — Source data Fig. 3 [file 44321_2024_62_MOESM6_ESM.zip › Figure 3/Fig3B/Fig3B-scramble shRNA-DAPI.tif]

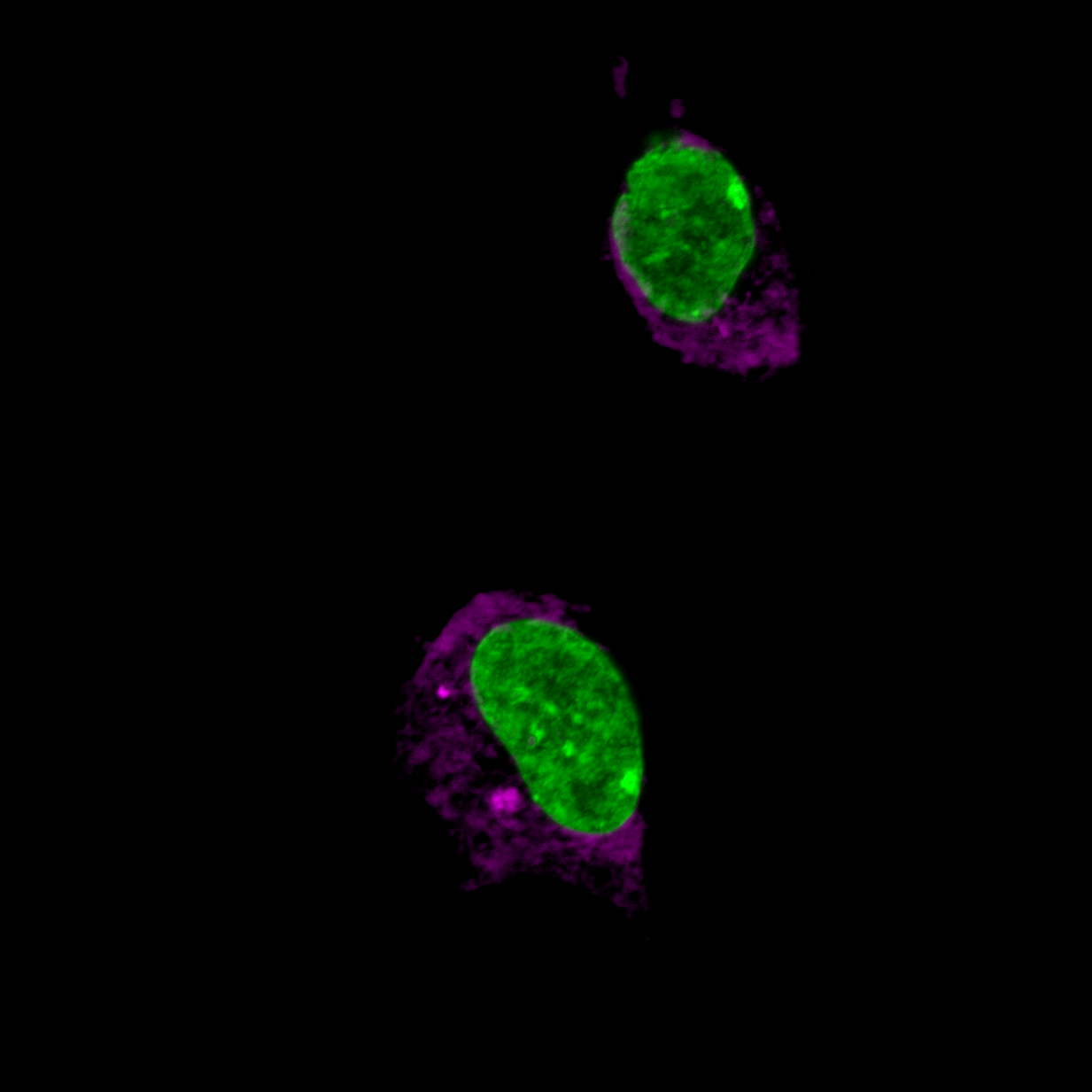

Supplement: Supplementary file 6 — Source data Fig. 3 [file 44321_2024_62_MOESM6_ESM.zip › Figure 3/Fig3B/Fig3B-scramble shRNA-Merge.tif]

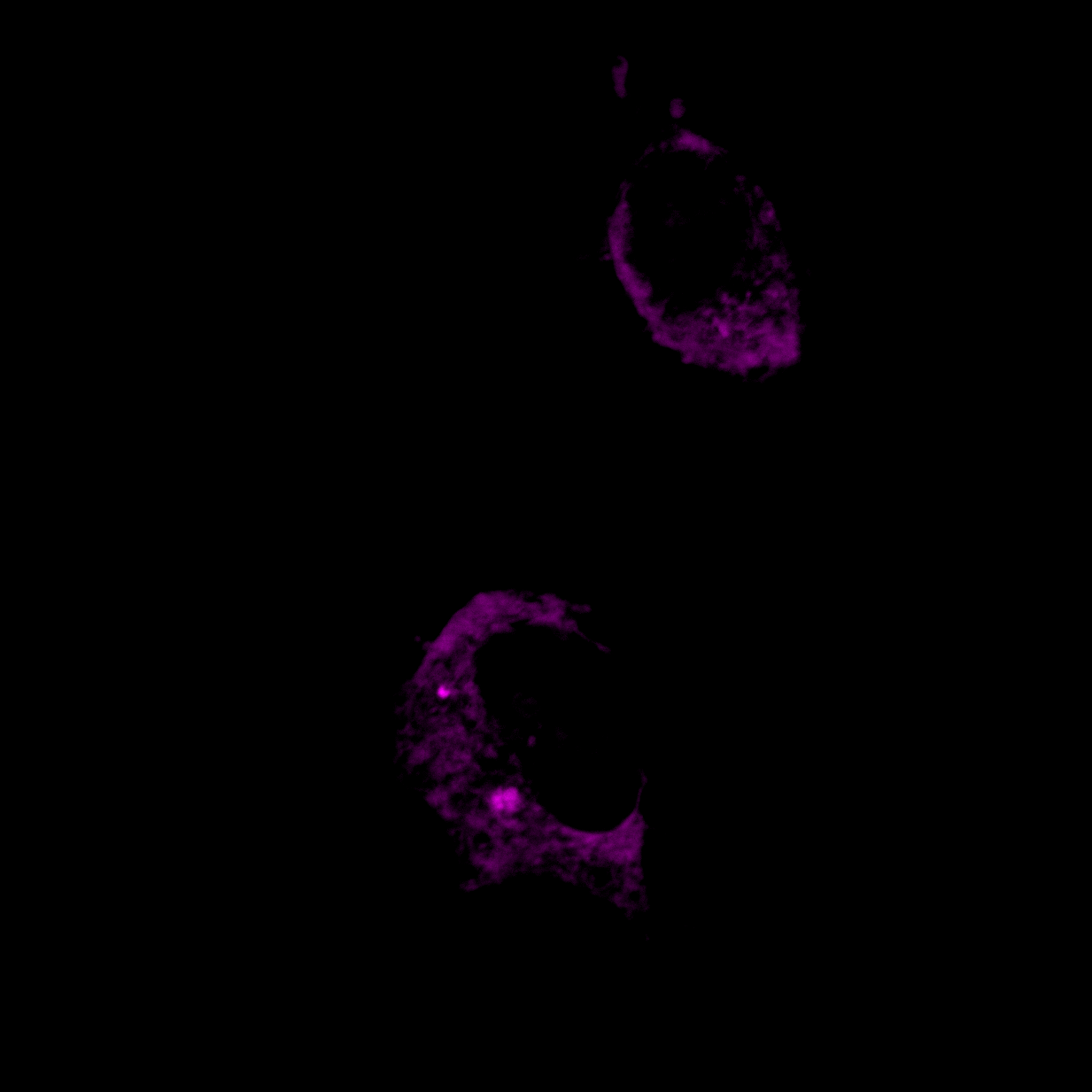

Supplement: Supplementary file 6 — Source data Fig. 3 [file 44321_2024_62_MOESM6_ESM.zip › Figure 3/Fig3B/Fig3B-scramble shRNA-PI.tif]

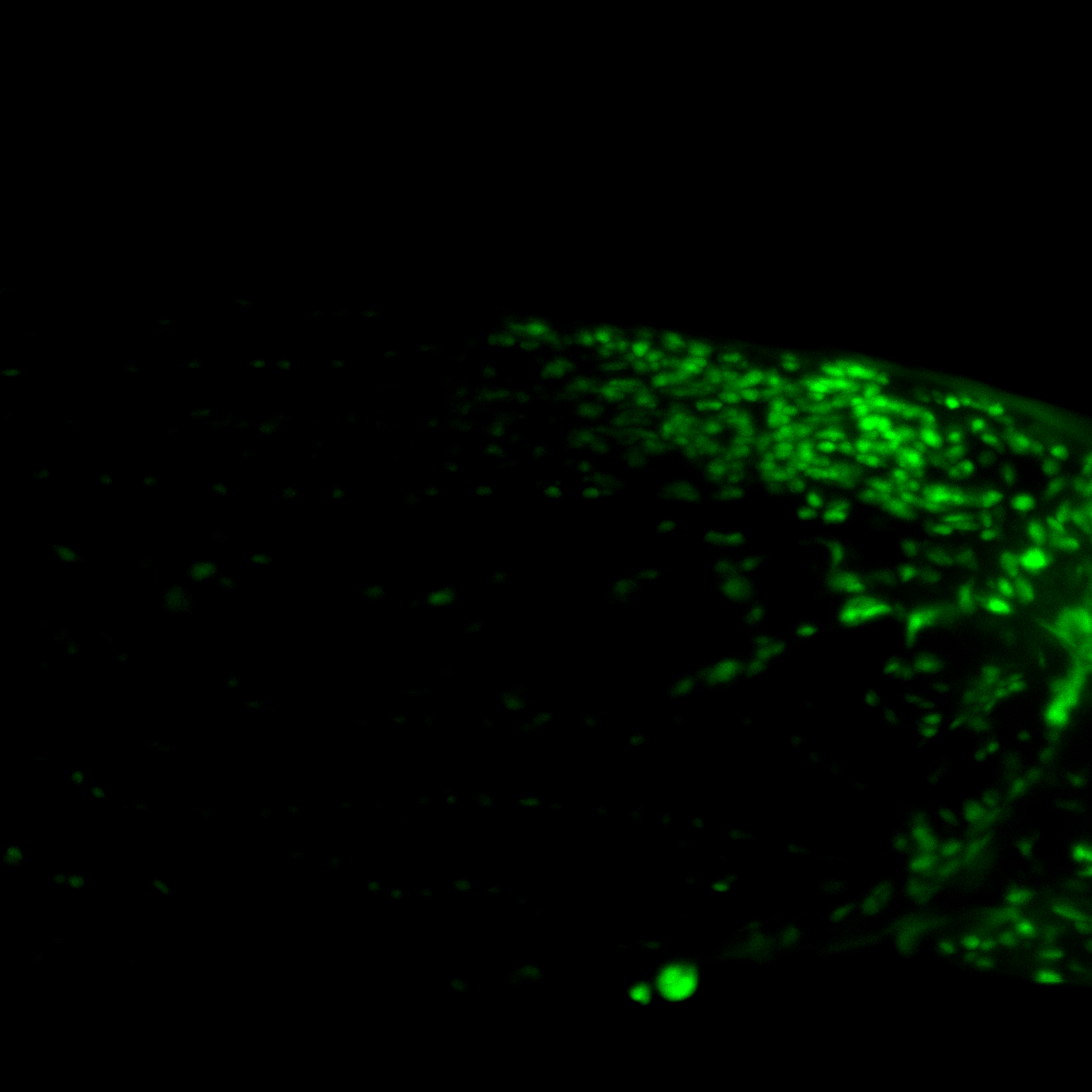

Supplement: Supplementary file 6 — Source data Fig. 3 [file 44321_2024_62_MOESM6_ESM.zip › Figure 3/Fig3D/Fig3D-W1-UAS-Baz-RNAi-DAPI.tif]

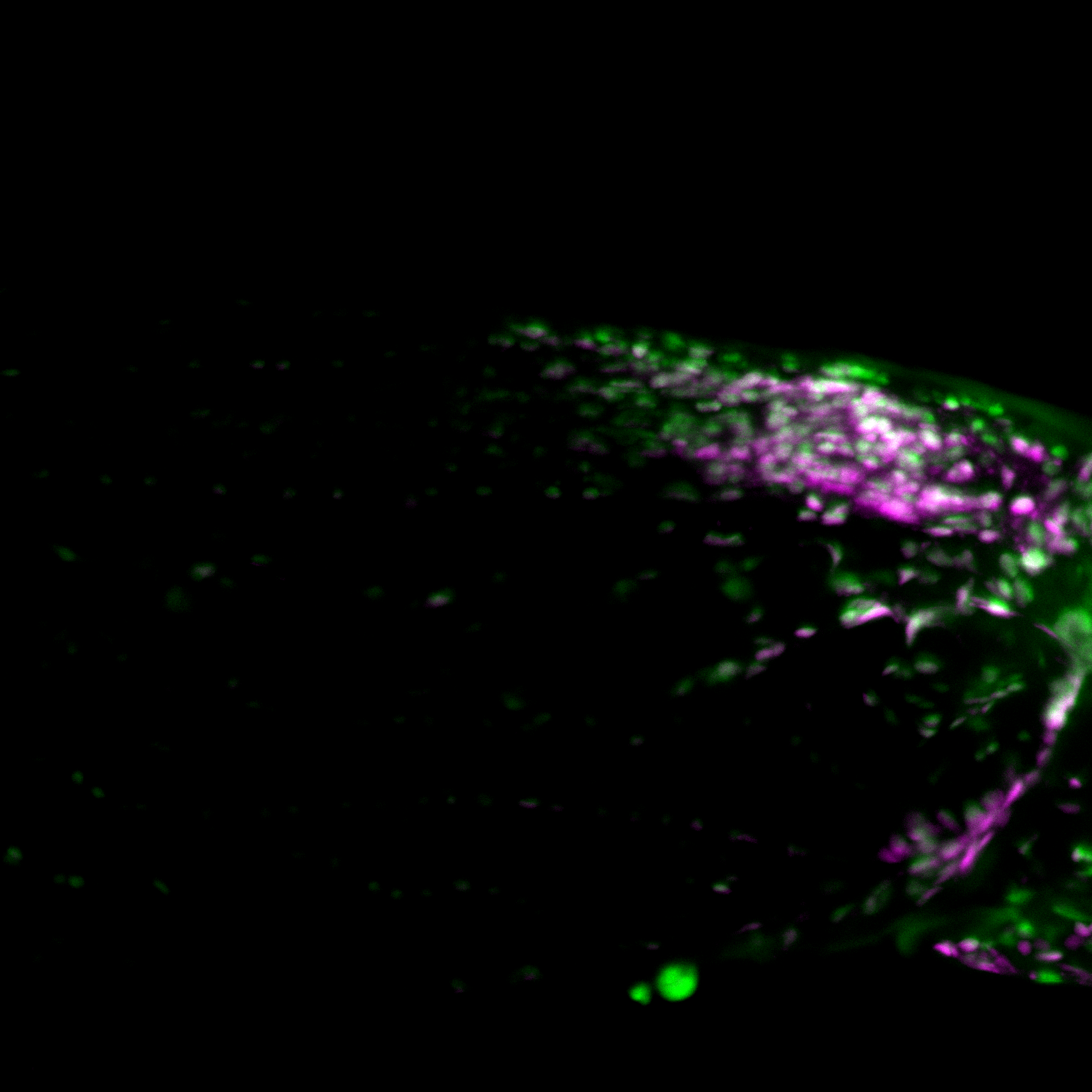

Supplement: Supplementary file 6 — Source data Fig. 3 [file 44321_2024_62_MOESM6_ESM.zip › Figure 3/Fig3D/Fig3D-W1-UAS-Baz-RNAi-Merge.tif]

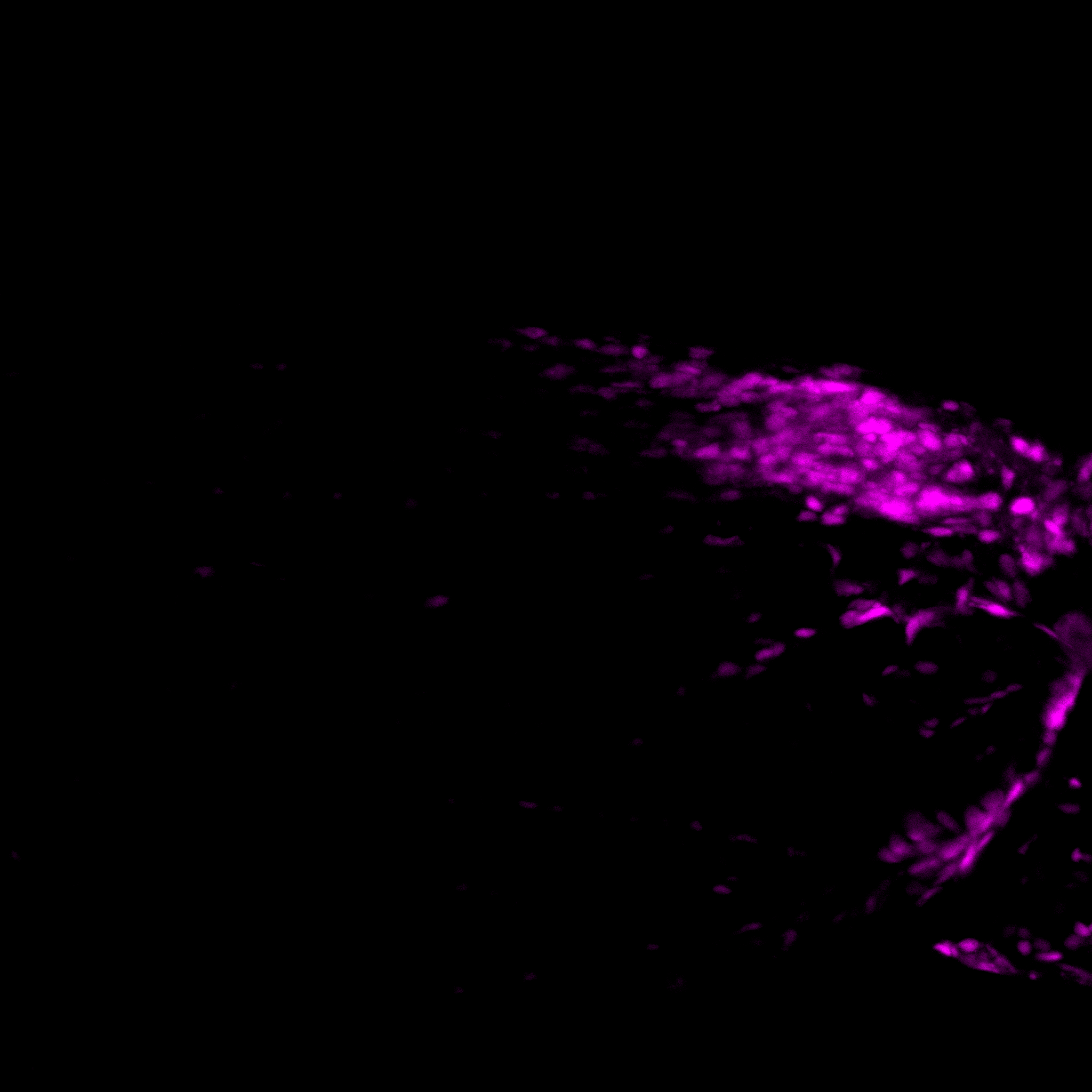

Supplement: Supplementary file 6 — Source data Fig. 3 [file 44321_2024_62_MOESM6_ESM.zip › Figure 3/Fig3D/Fig3D-W1-UAS-Baz-RNAi-PI.tif]

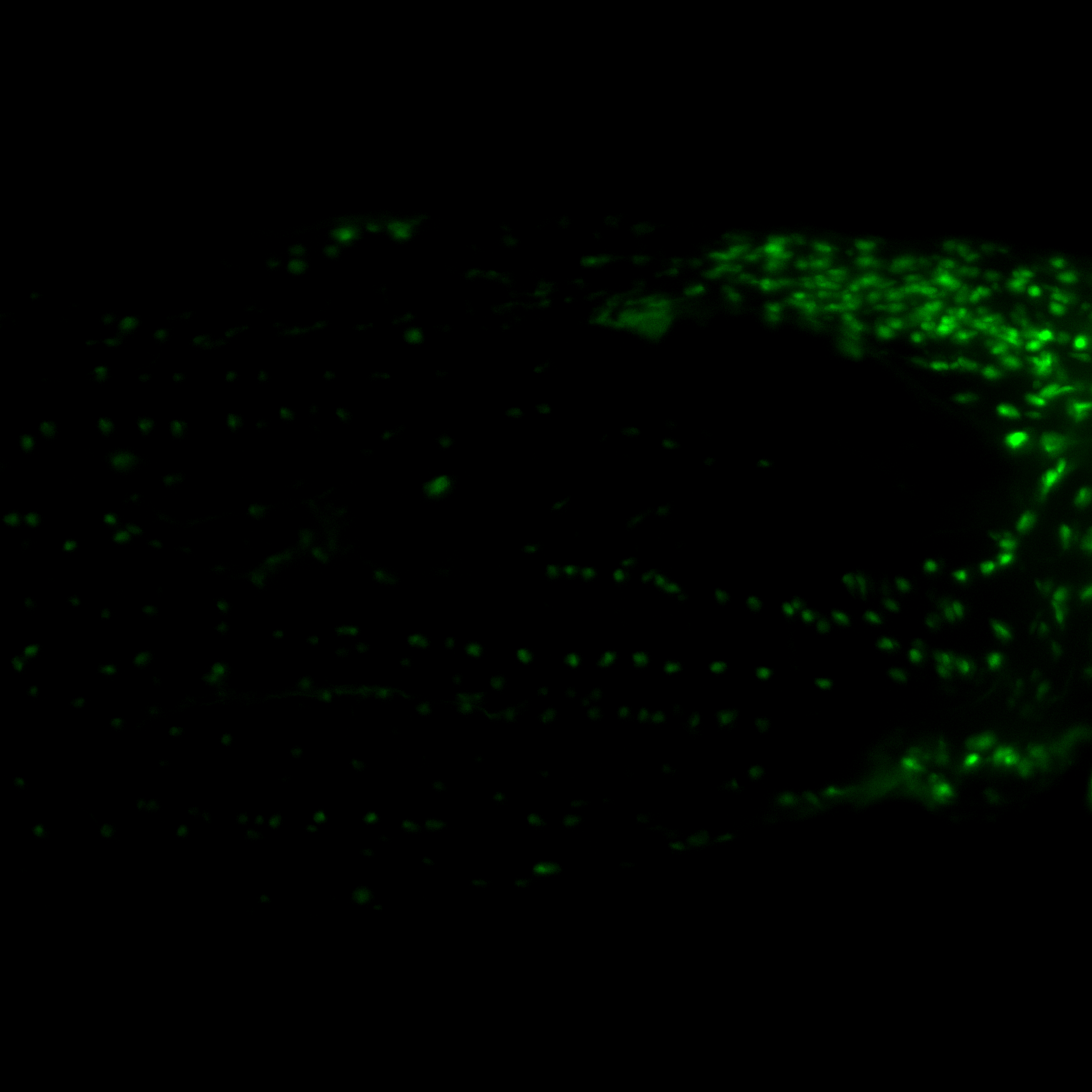

Supplement: Supplementary file 6 — Source data Fig. 3 [file 44321_2024_62_MOESM6_ESM.zip › Figure 3/Fig3D/Fig3D-W1-UAS-Insc-RNAi-DAPI.tif]

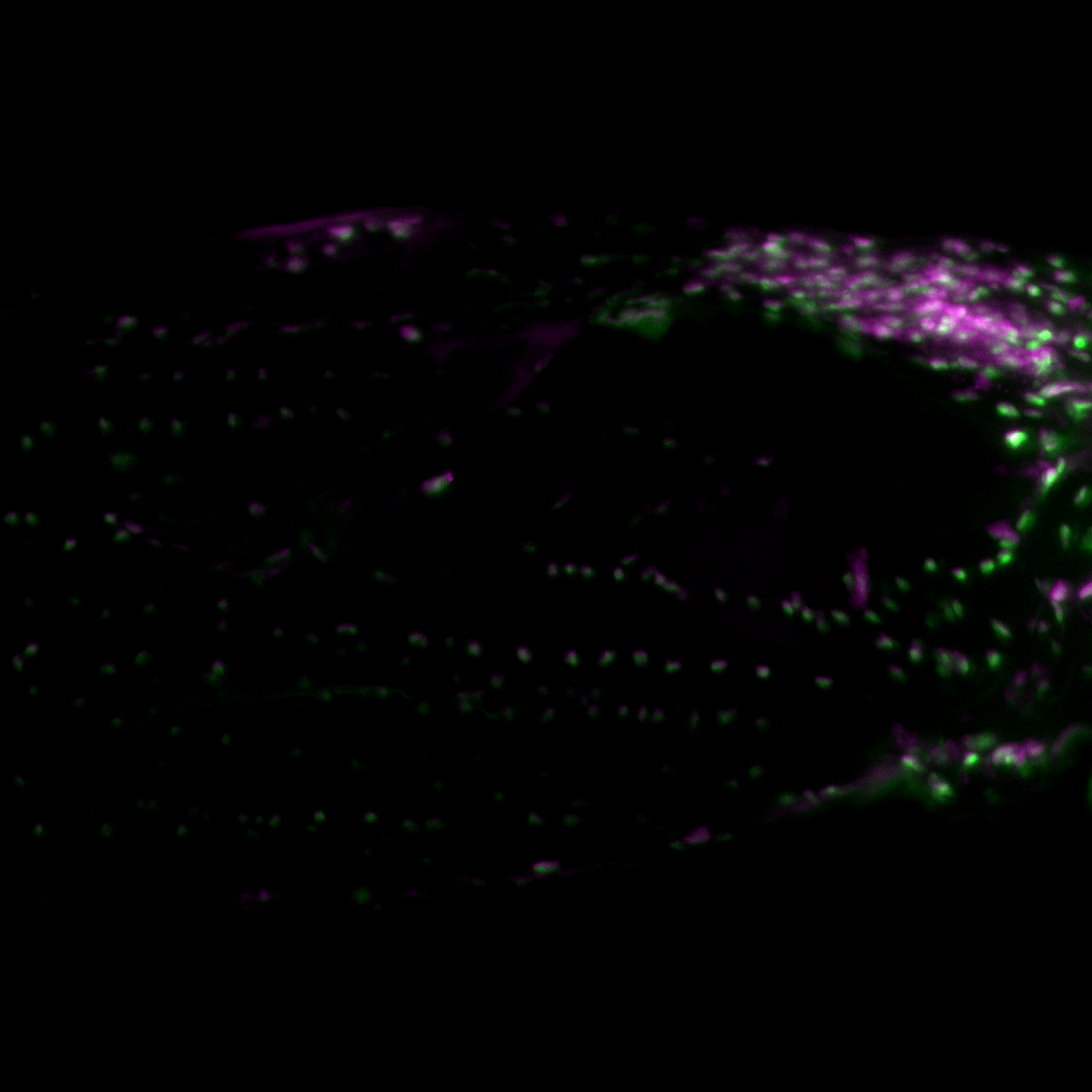

Supplement: Supplementary file 6 — Source data Fig. 3 [file 44321_2024_62_MOESM6_ESM.zip › Figure 3/Fig3D/Fig3D-W1-UAS-Insc-RNAi-Merge.tif]

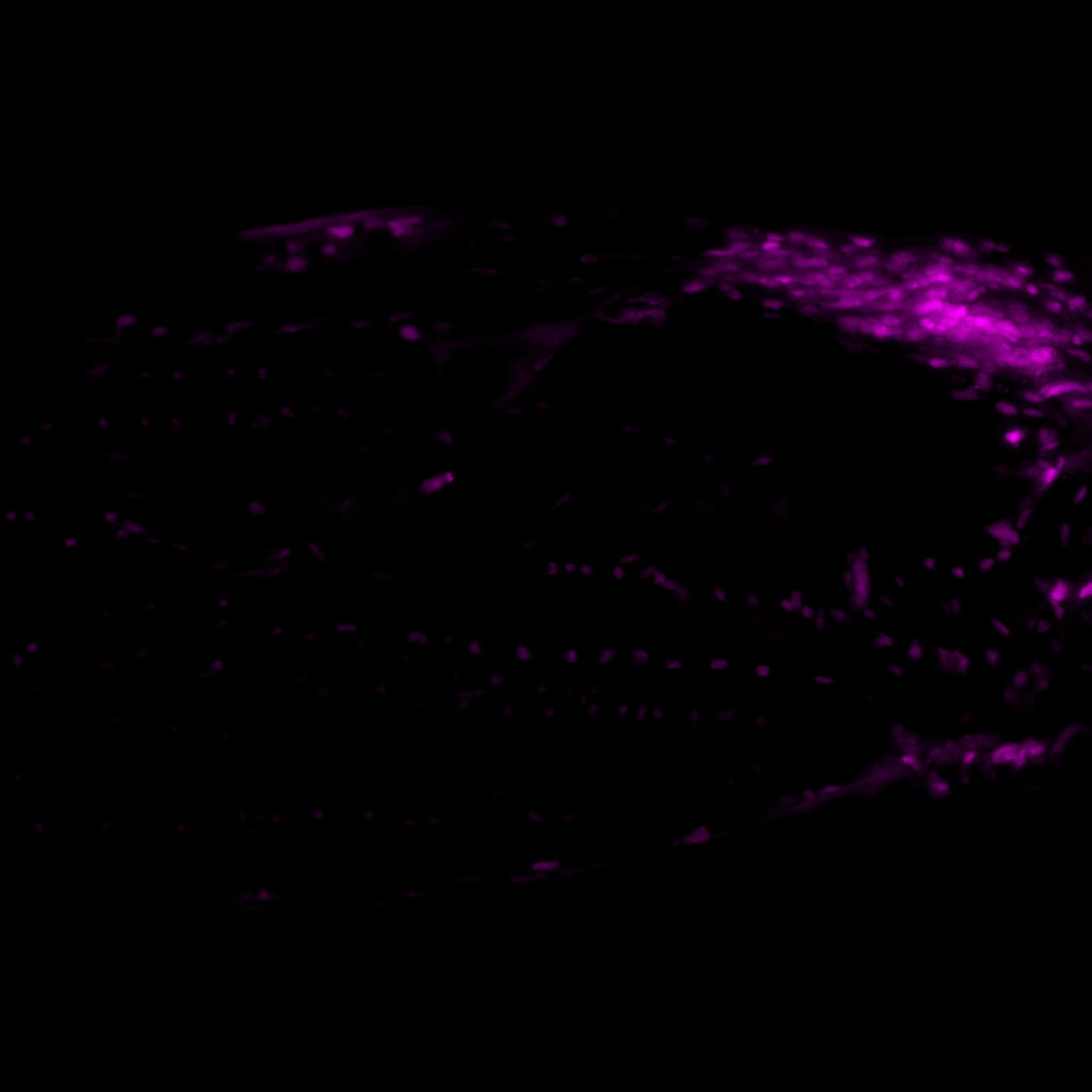

Supplement: Supplementary file 6 — Source data Fig. 3 [file 44321_2024_62_MOESM6_ESM.zip › Figure 3/Fig3D/Fig3D-W1-UAS-Insc-RNAi-PI.tif]

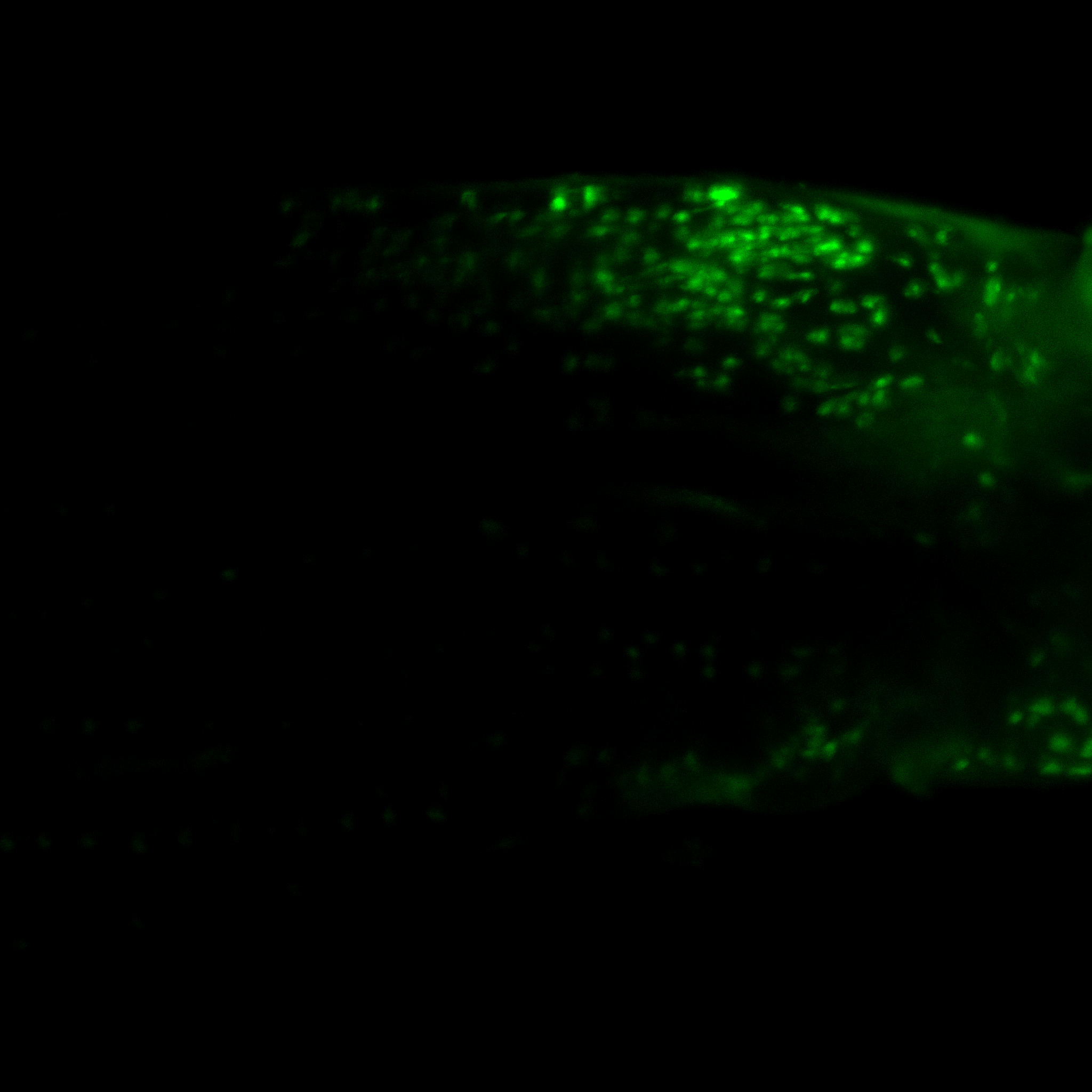

Supplement: Supplementary file 6 — Source data Fig. 3 [file 44321_2024_62_MOESM6_ESM.zip › Figure 3/Fig3D/Fig3D-W1-UAS-Pins-RNAi-DAPI.tif]

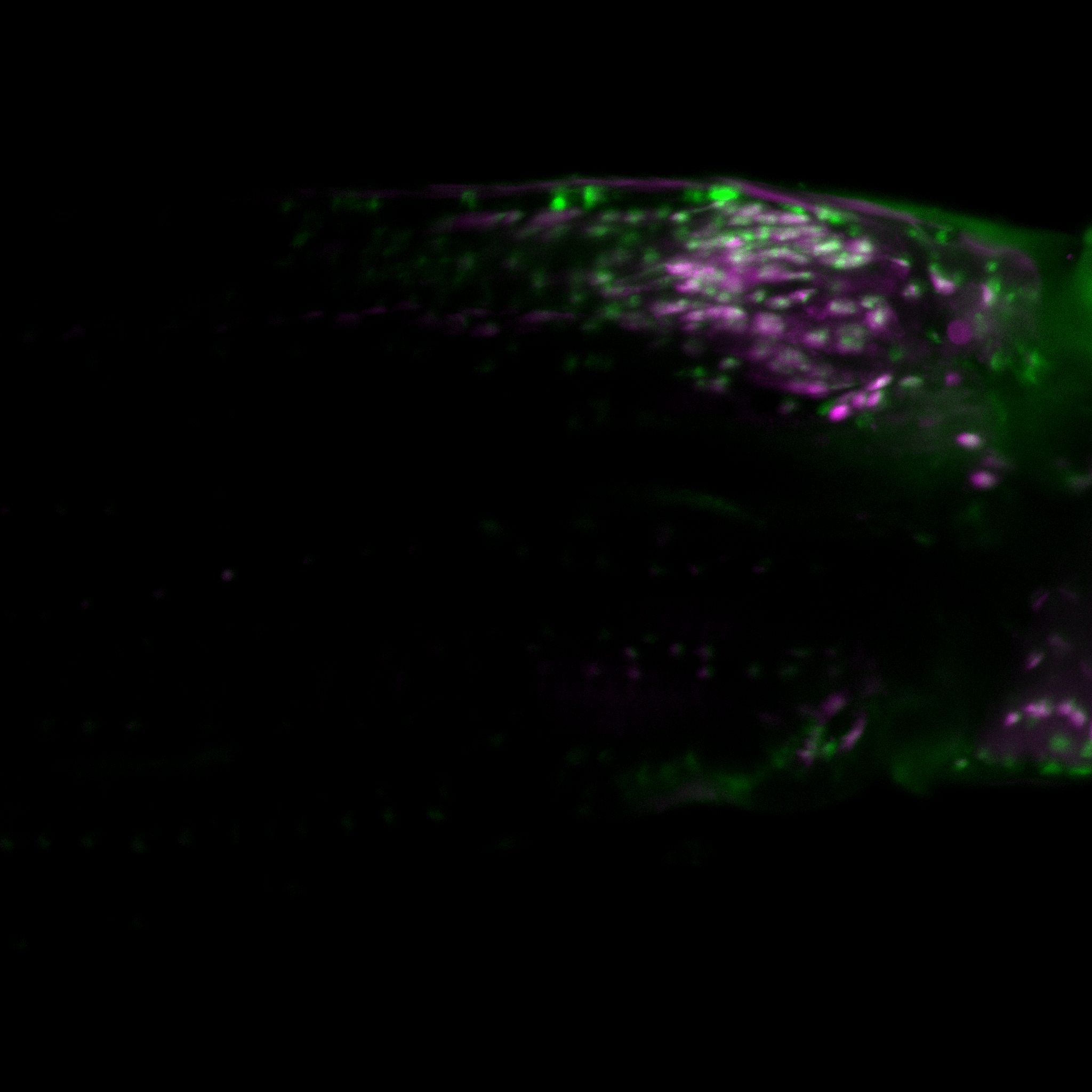

Supplement: Supplementary file 6 — Source data Fig. 3 [file 44321_2024_62_MOESM6_ESM.zip › Figure 3/Fig3D/Fig3D-W1-UAS-Pins-RNAi-Merge.tif]

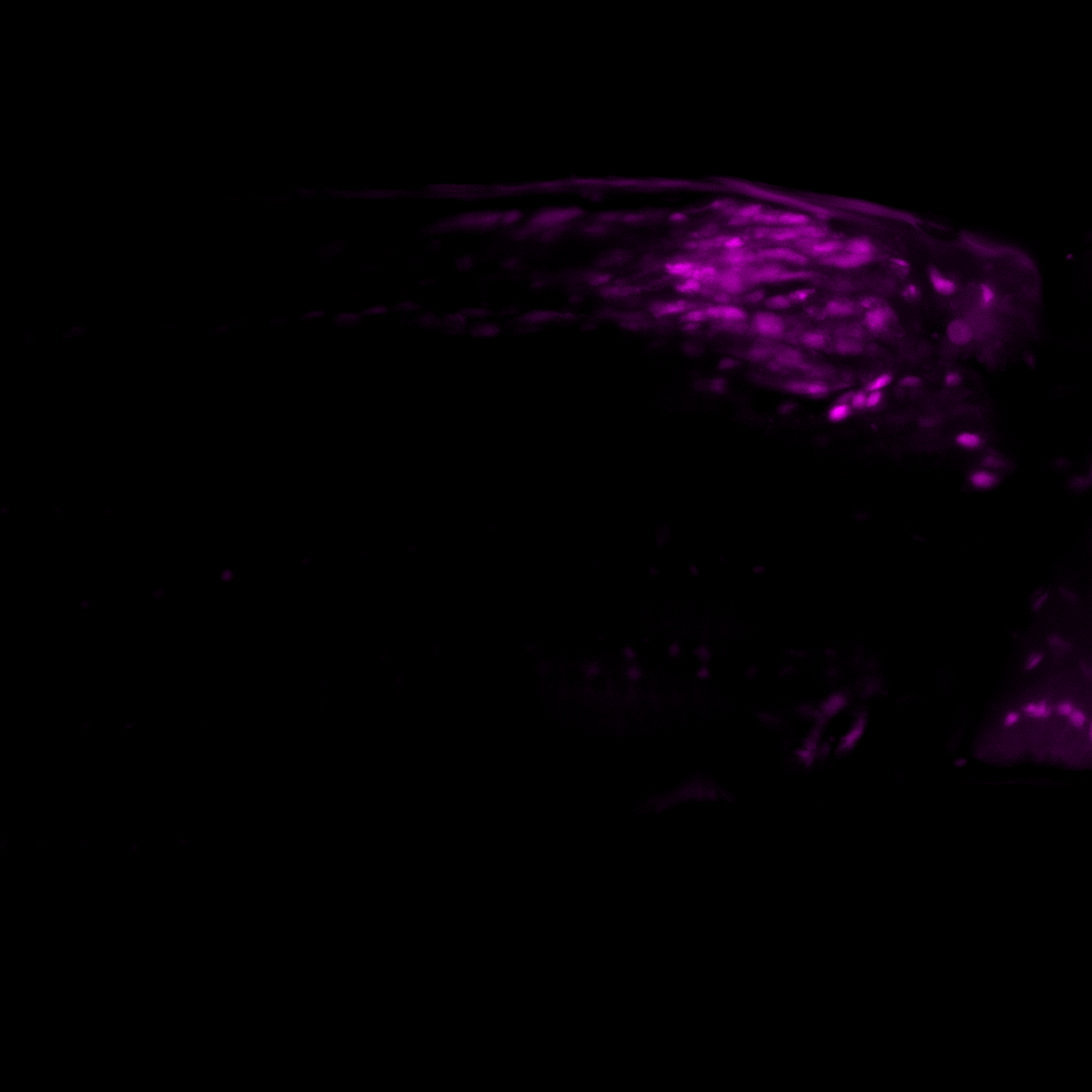

Supplement: Supplementary file 6 — Source data Fig. 3 [file 44321_2024_62_MOESM6_ESM.zip › Figure 3/Fig3D/Fig3D-W1-UAS-Pins-RNAi-PI.tif]

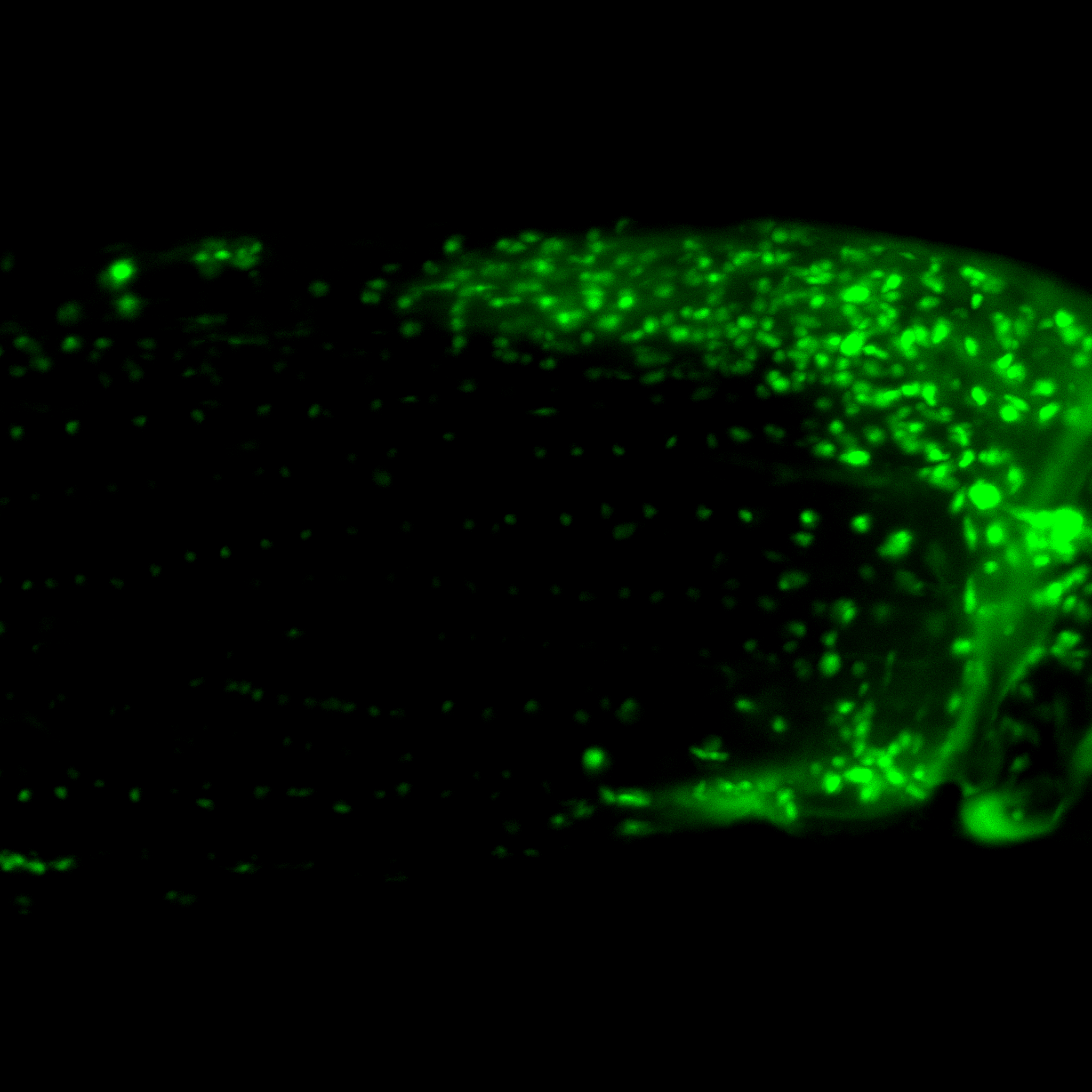

Supplement: Supplementary file 6 — Source data Fig. 3 [file 44321_2024_62_MOESM6_ESM.zip › Figure 3/Fig3D/Fig3D-W1-UAS-w-RNAi-DAPI.tif]

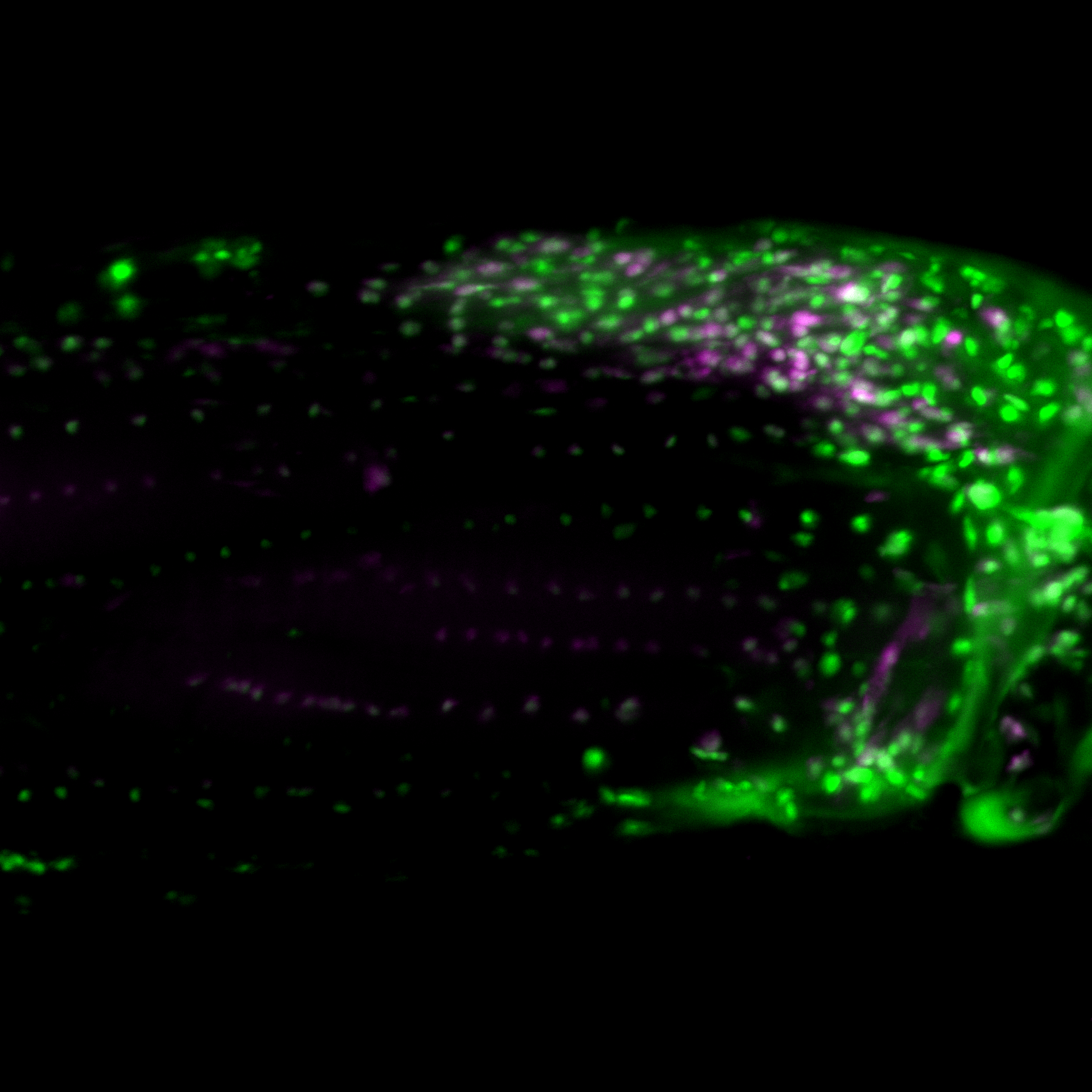

Supplement: Supplementary file 6 — Source data Fig. 3 [file 44321_2024_62_MOESM6_ESM.zip › Figure 3/Fig3D/Fig3D-W1-UAS-w-RNAi-Merge.tif]

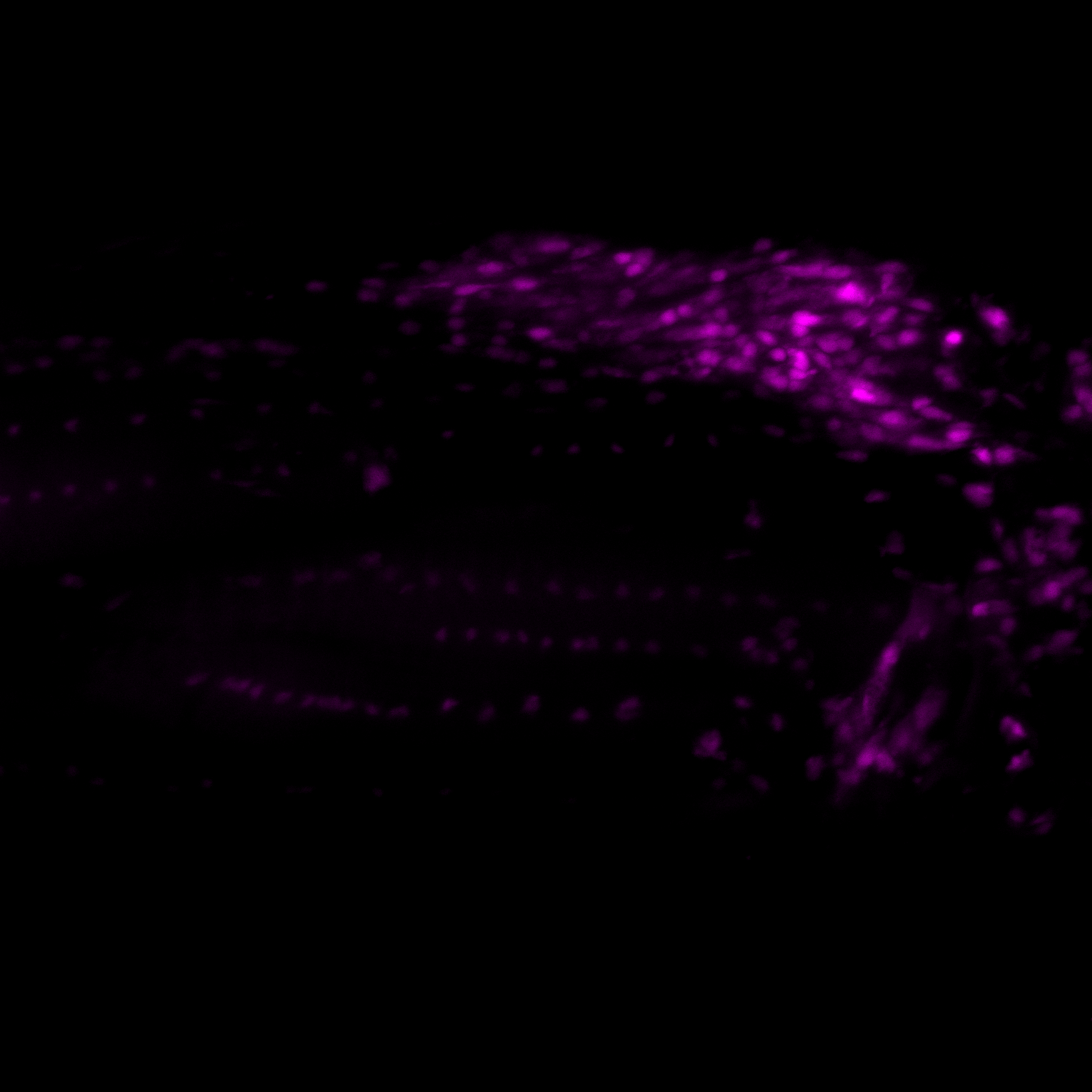

Supplement: Supplementary file 6 — Source data Fig. 3 [file 44321_2024_62_MOESM6_ESM.zip › Figure 3/Fig3D/Fig3D-W1-UAS-w-RNAi-PI.tif]

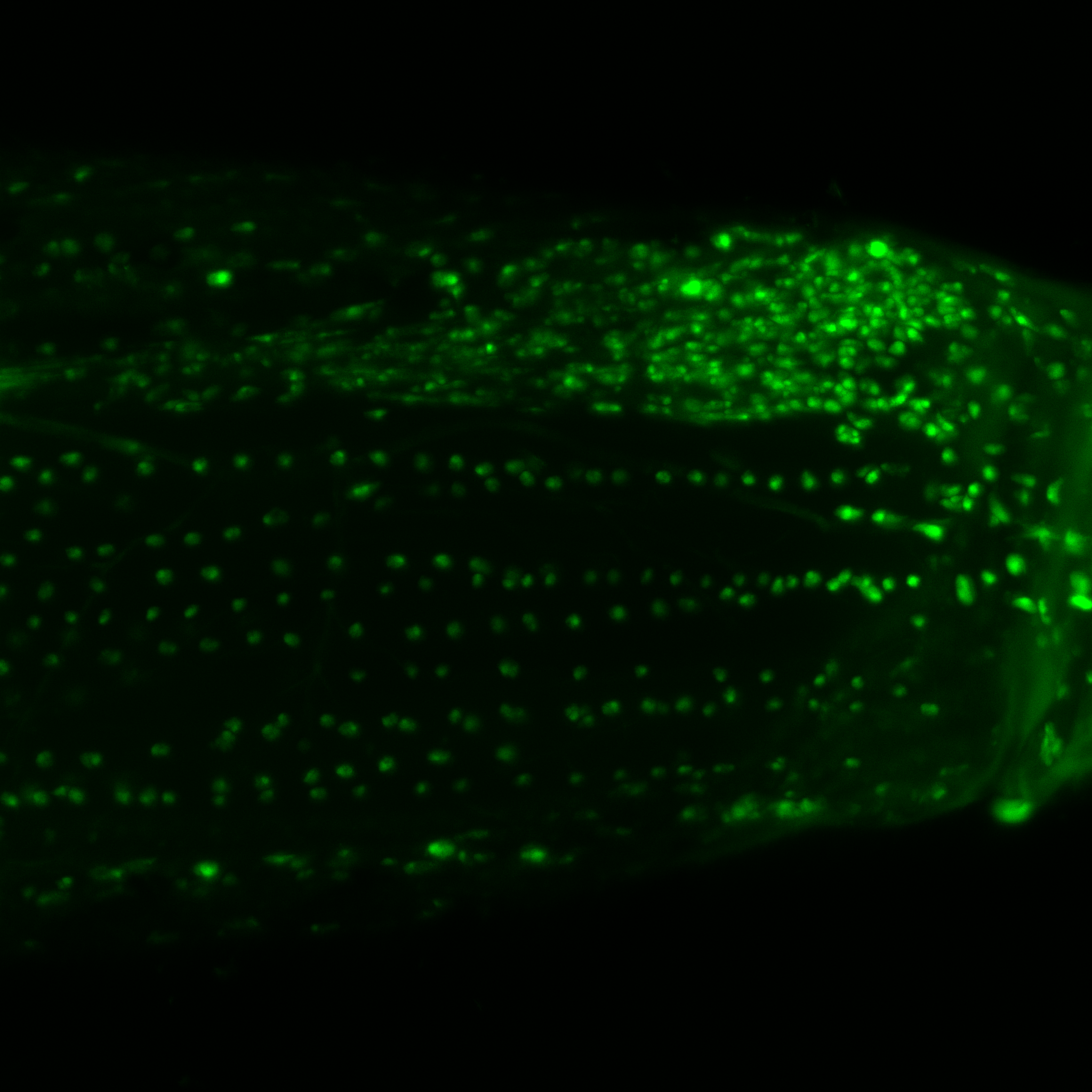

Supplement: Supplementary file 6 — Source data Fig. 3 [file 44321_2024_62_MOESM6_ESM.zip › Figure 3/Fig3D/Fig3D-W3-UAS-Baz-RNAi-DAPI.tif]

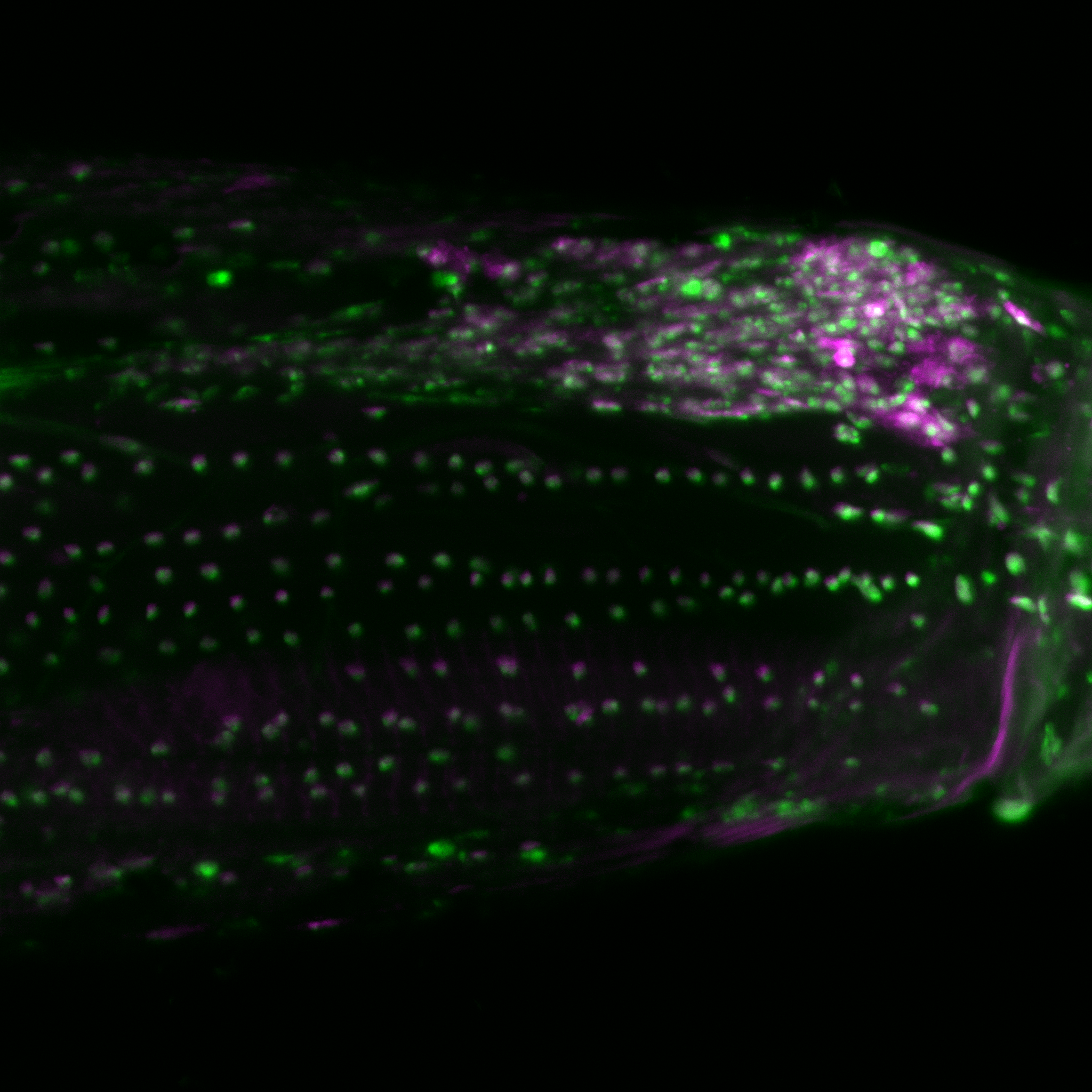

Supplement: Supplementary file 6 — Source data Fig. 3 [file 44321_2024_62_MOESM6_ESM.zip › Figure 3/Fig3D/Fig3D-W3-UAS-Baz-RNAi-Merge.tif]

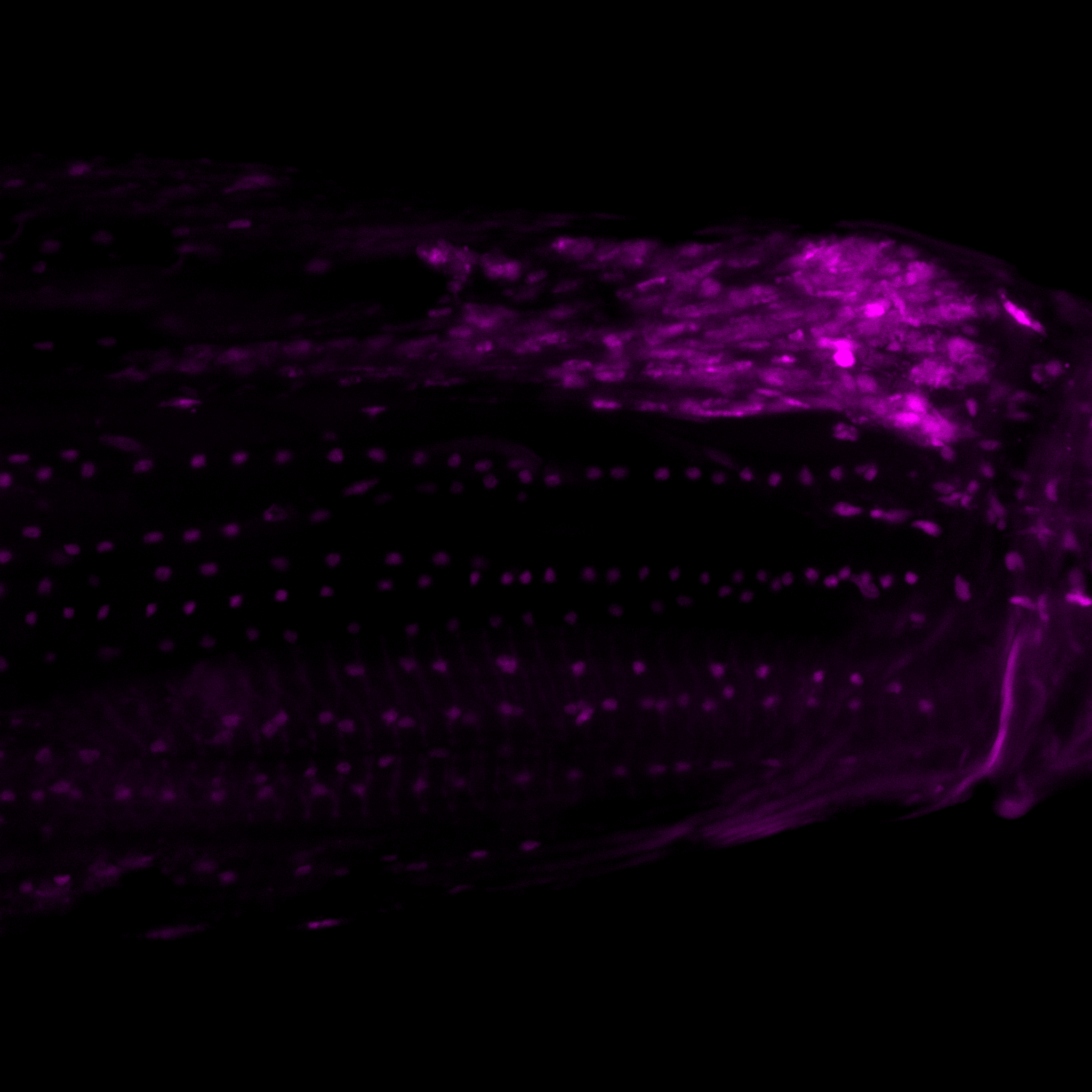

Supplement: Supplementary file 6 — Source data Fig. 3 [file 44321_2024_62_MOESM6_ESM.zip › Figure 3/Fig3D/Fig3D-W3-UAS-Baz-RNAi-PI.tif]

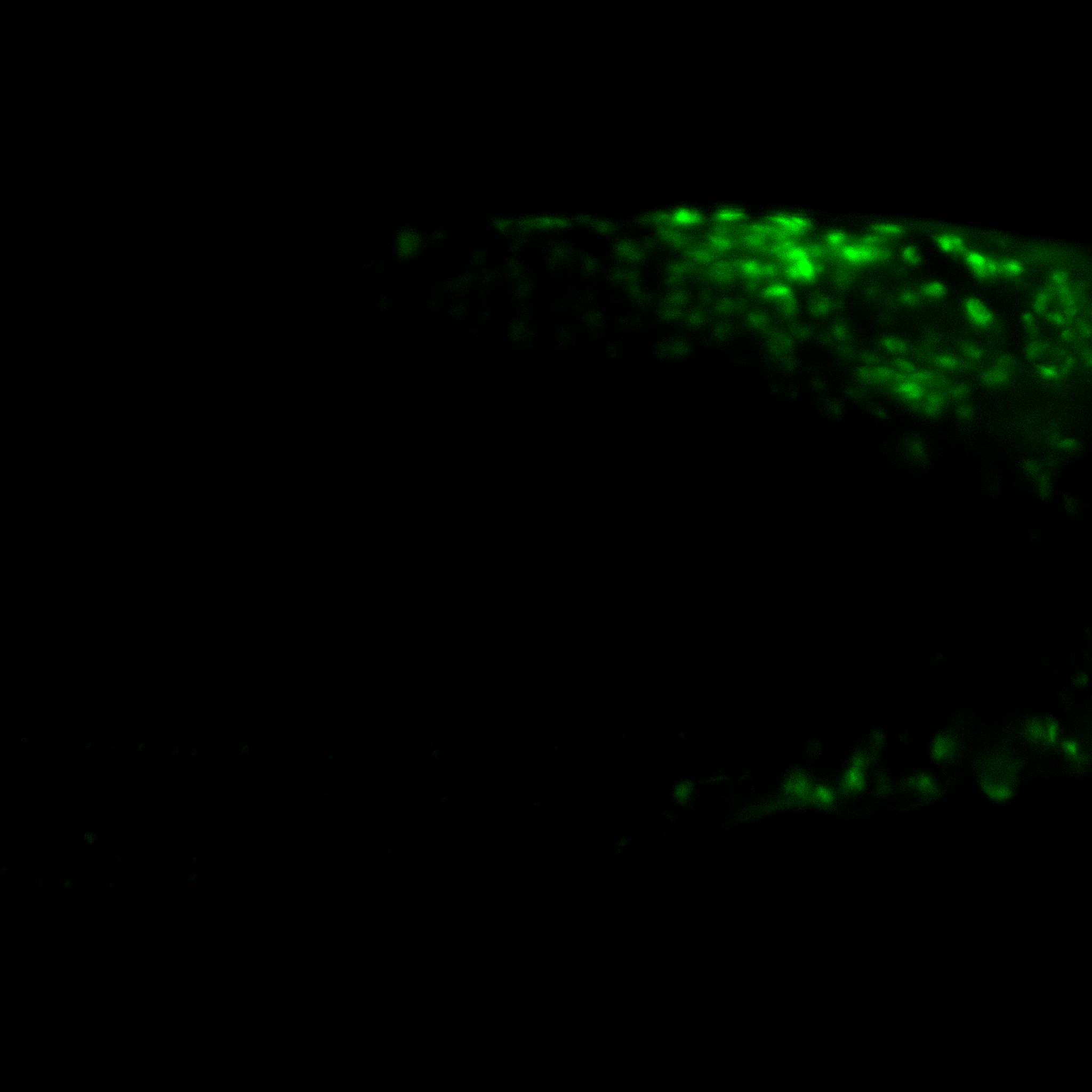

Supplement: Supplementary file 6 — Source data Fig. 3 [file 44321_2024_62_MOESM6_ESM.zip › Figure 3/Fig3D/Fig3D-W3-UAS-Insc-RNAi-DAPI.tif]

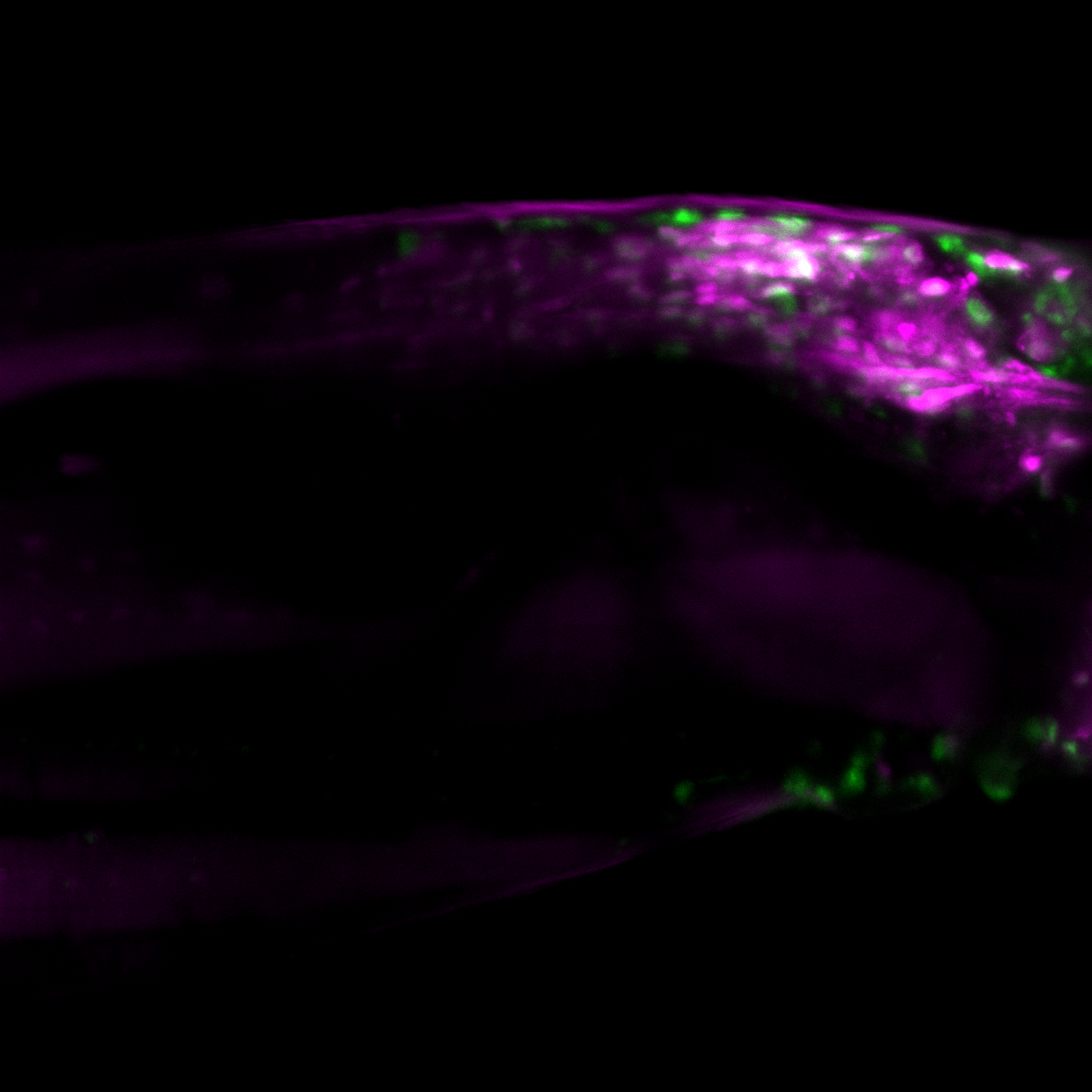

Supplement: Supplementary file 6 — Source data Fig. 3 [file 44321_2024_62_MOESM6_ESM.zip › Figure 3/Fig3D/Fig3D-W3-UAS-Insc-RNAi-Merge.tif]

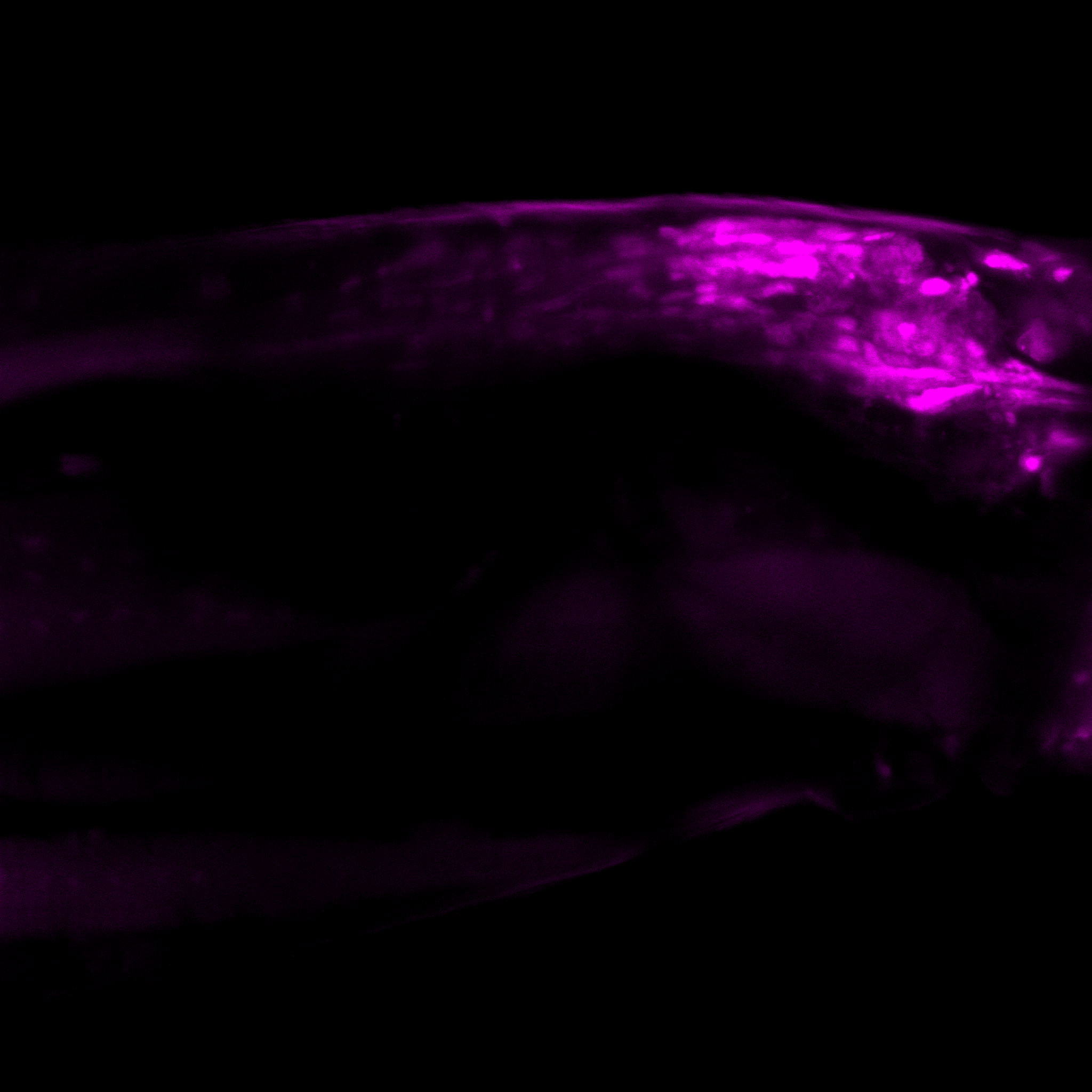

Supplement: Supplementary file 6 — Source data Fig. 3 [file 44321_2024_62_MOESM6_ESM.zip › Figure 3/Fig3D/Fig3D-W3-UAS-Insc-RNAi-PI.tif]

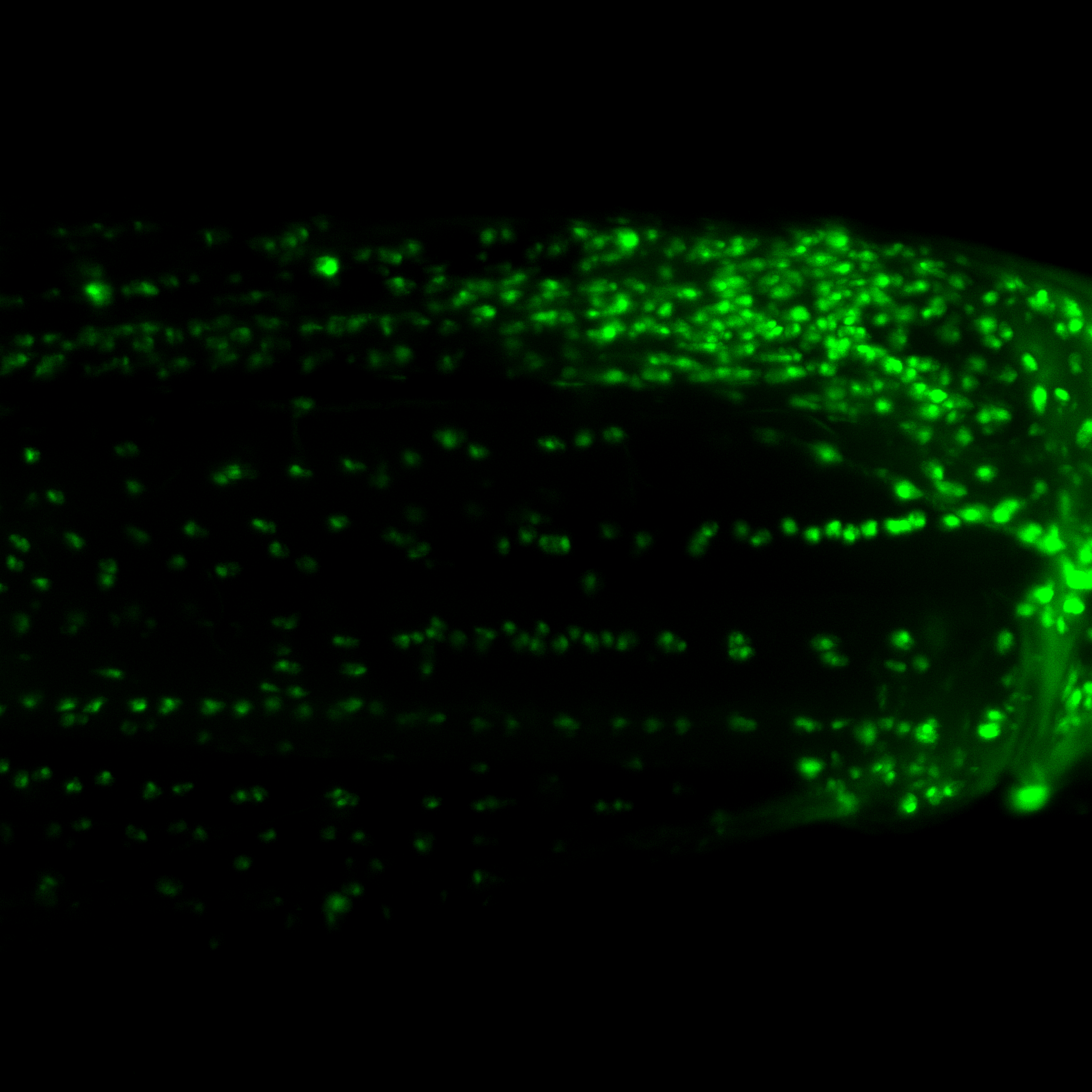

Supplement: Supplementary file 6 — Source data Fig. 3 [file 44321_2024_62_MOESM6_ESM.zip › Figure 3/Fig3D/Fig3D-W3-UAS-Pins-RNAi-DAPI.tif]

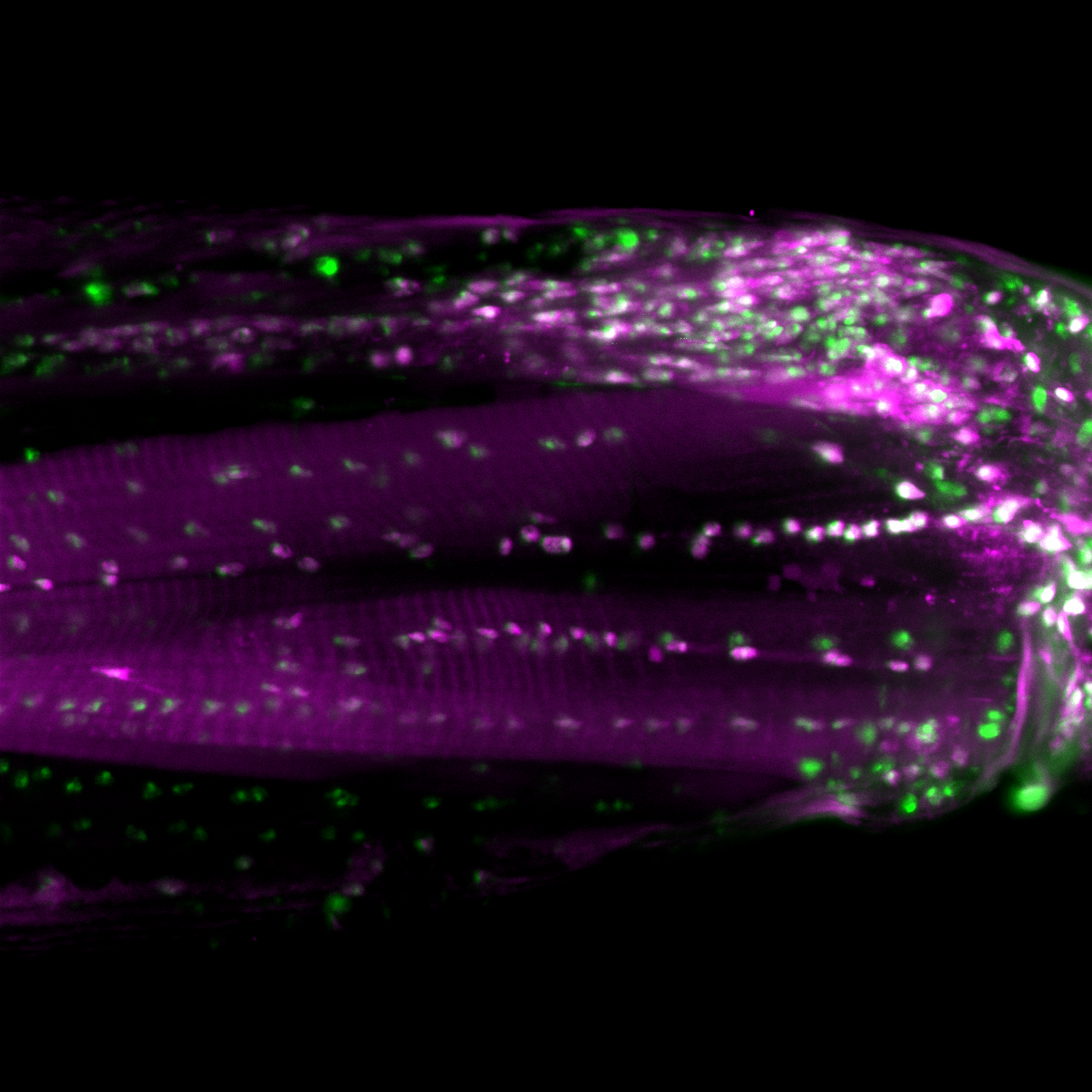

Supplement: Supplementary file 6 — Source data Fig. 3 [file 44321_2024_62_MOESM6_ESM.zip › Figure 3/Fig3D/Fig3D-W3-UAS-Pins-RNAi-Merge.tif]

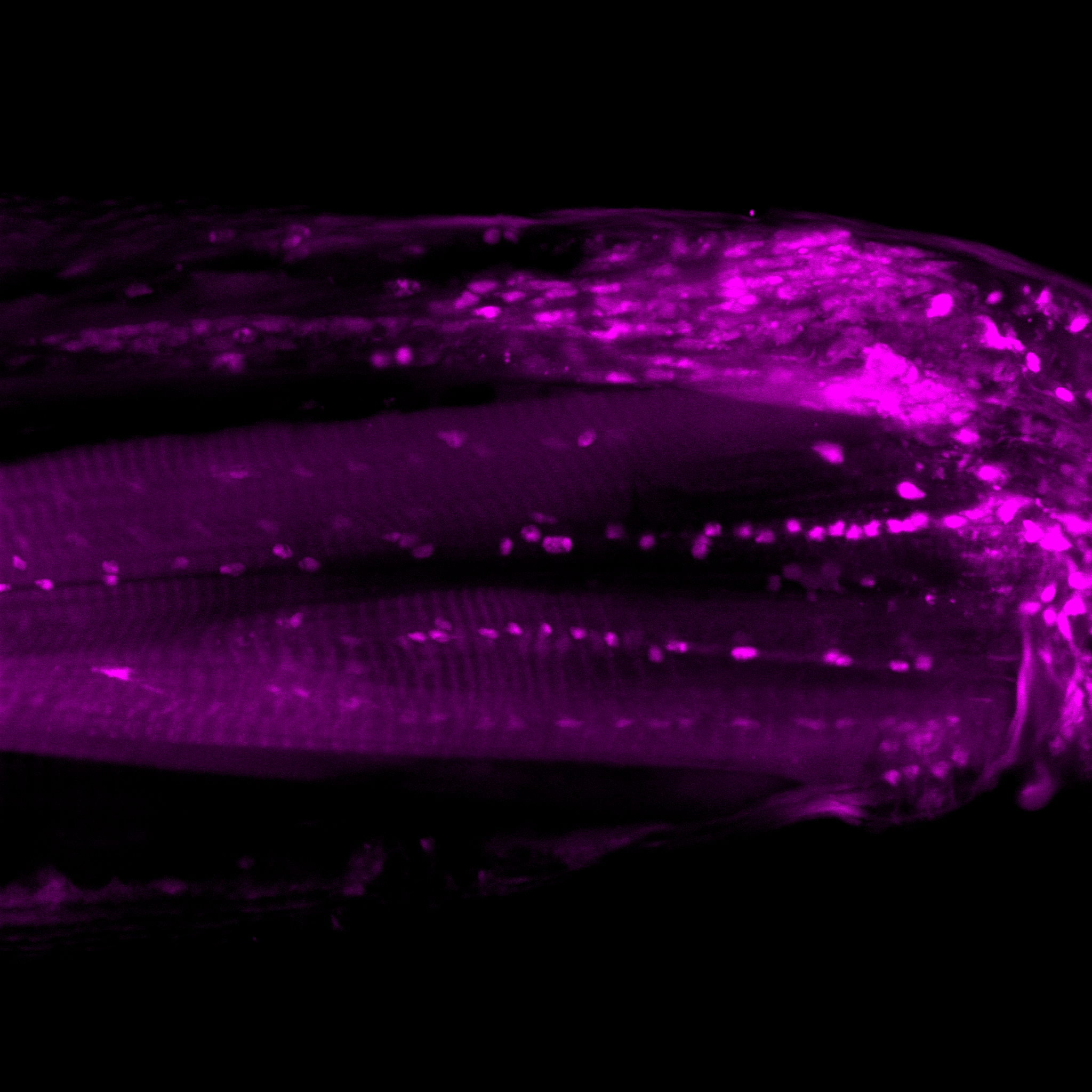

Supplement: Supplementary file 6 — Source data Fig. 3 [file 44321_2024_62_MOESM6_ESM.zip › Figure 3/Fig3D/Fig3D-W3-UAS-Pins-RNAi-PI.tif]

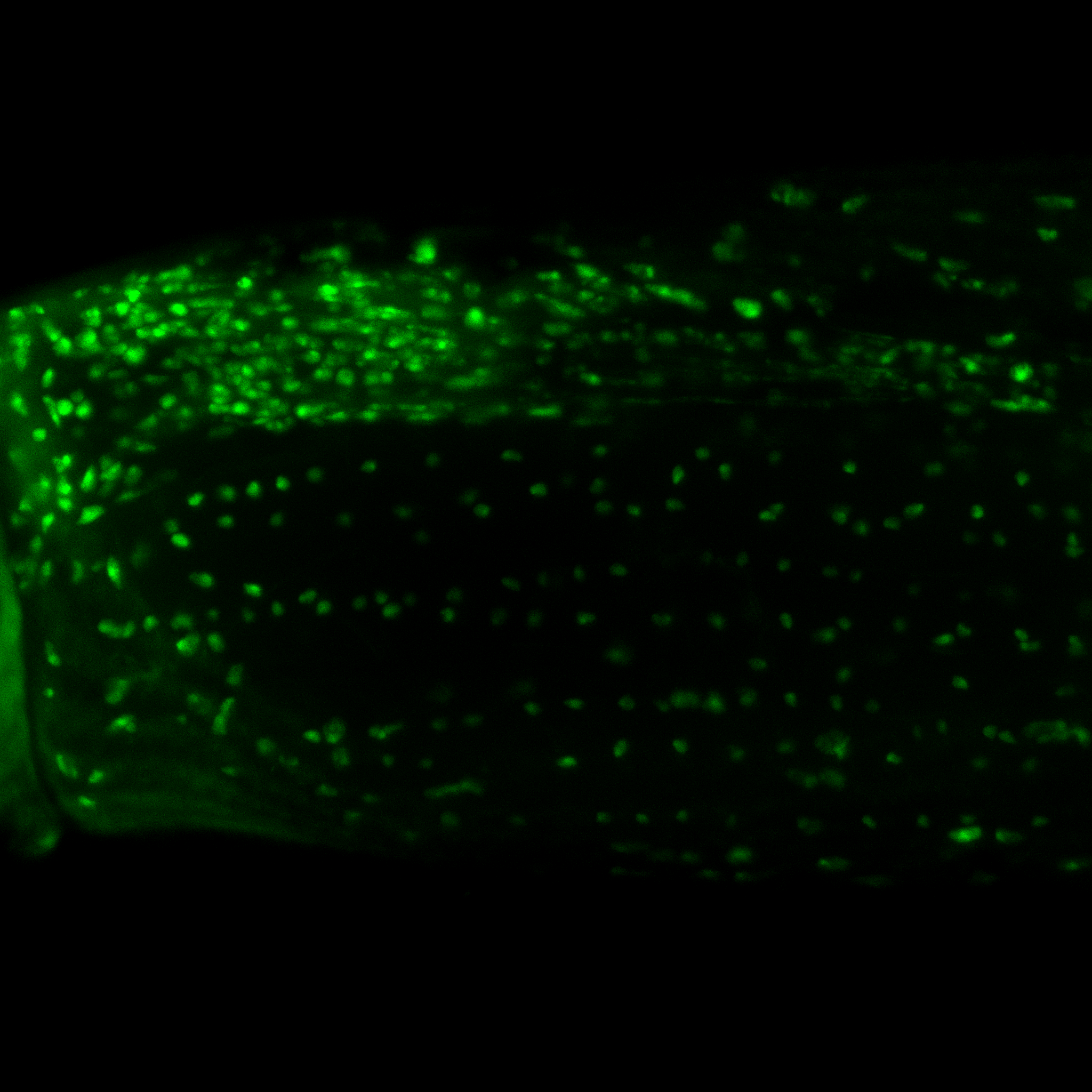

Supplement: Supplementary file 6 — Source data Fig. 3 [file 44321_2024_62_MOESM6_ESM.zip › Figure 3/Fig3D/Fig3D-W3-UAS-w-RNAi-DAPI.tif]

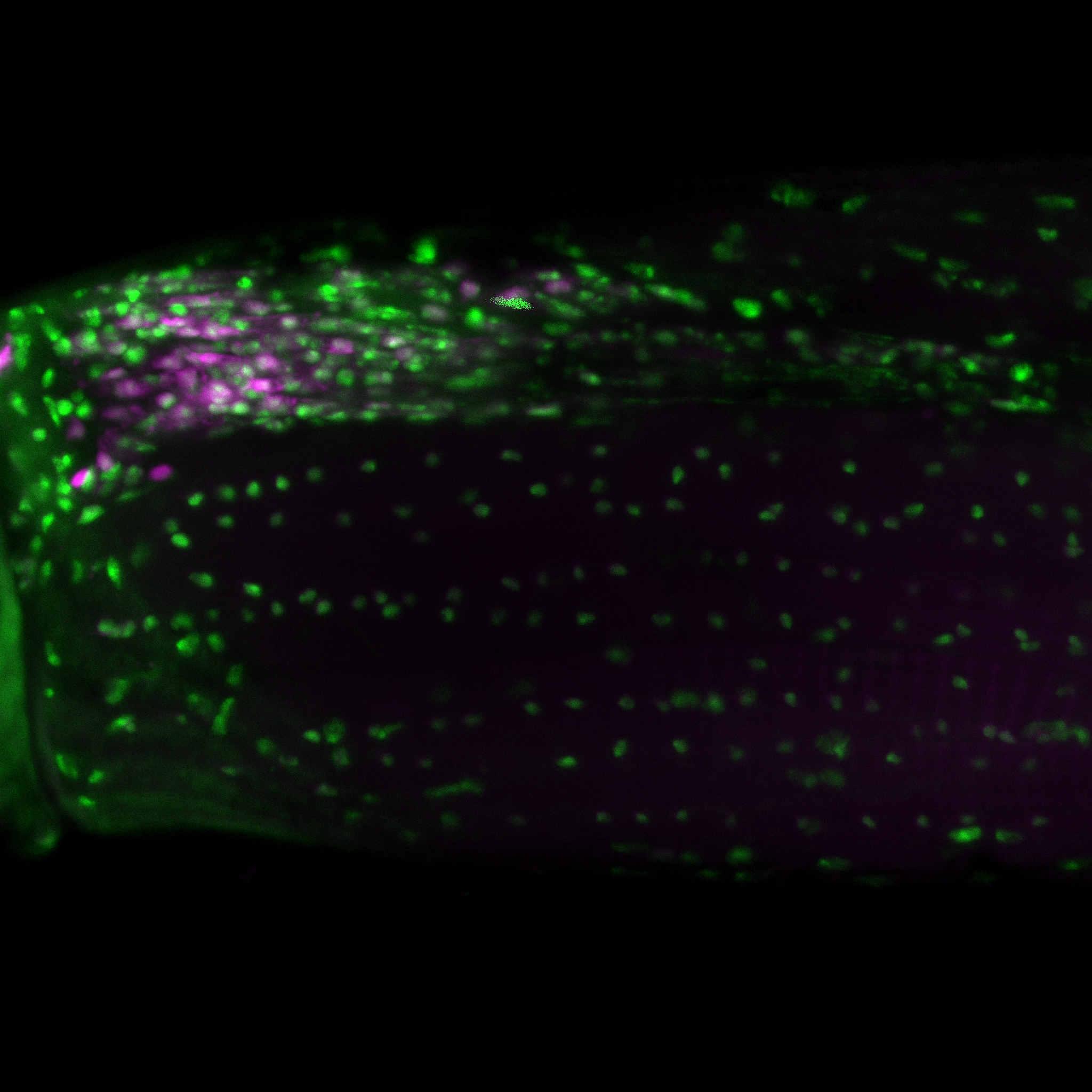

Supplement: Supplementary file 6 — Source data Fig. 3 [file 44321_2024_62_MOESM6_ESM.zip › Figure 3/Fig3D/Fig3D-W3-UAS-w-RNAi-Merge.tif]

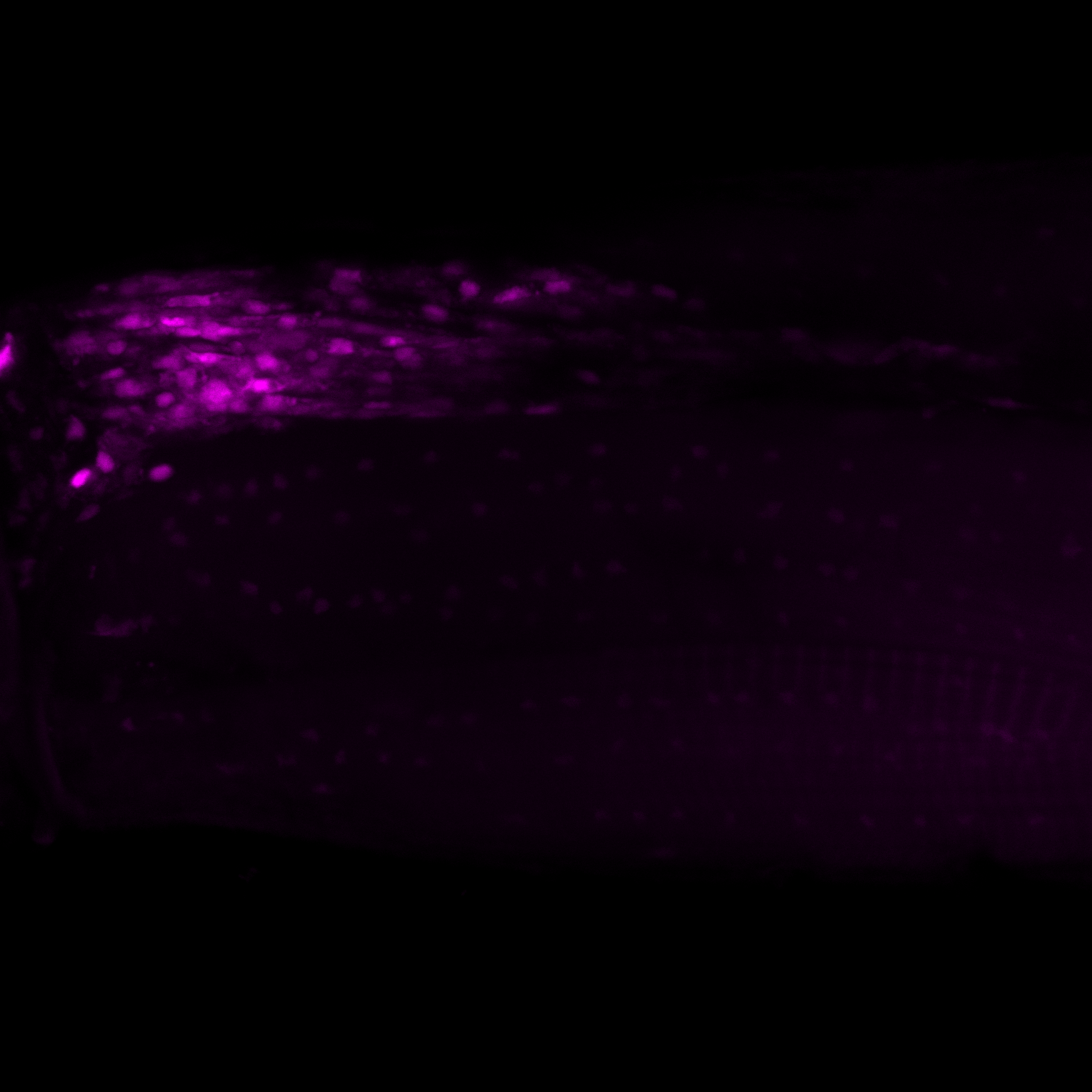

Supplement: Supplementary file 6 — Source data Fig. 3 [file 44321_2024_62_MOESM6_ESM.zip › Figure 3/Fig3D/Fig3D-W3-UAS-w-RNAi-PI.tif]

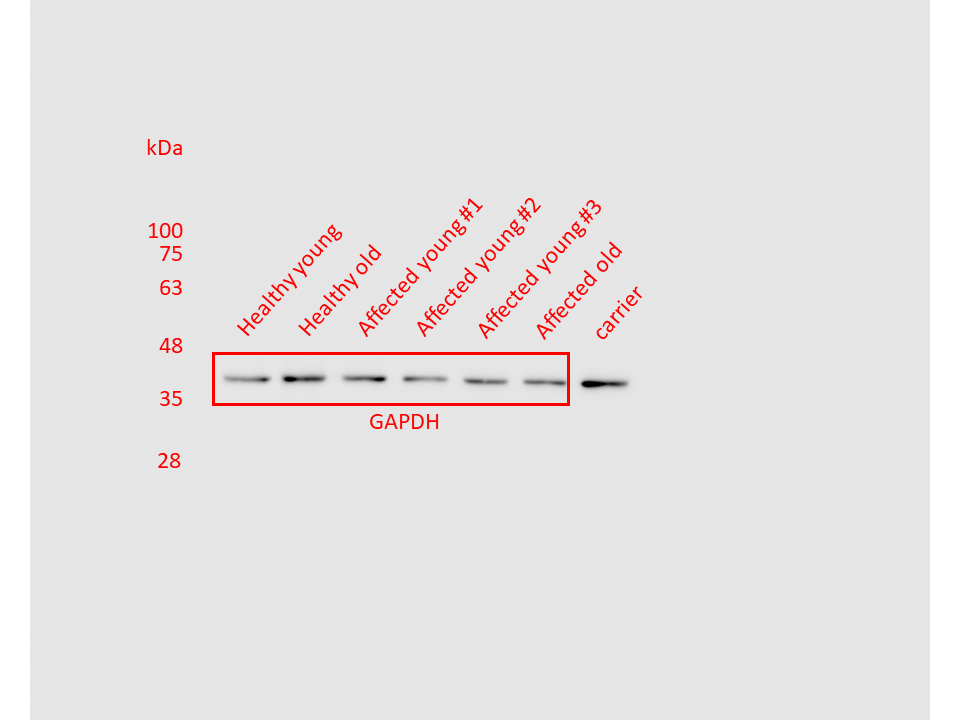

Supplement: Supplementary file 7 — Source data Fig. 4 [file 44321_2024_62_MOESM7_ESM.zip › Figure 4/Fig4B/Fig4B-GAPDH.TIF]

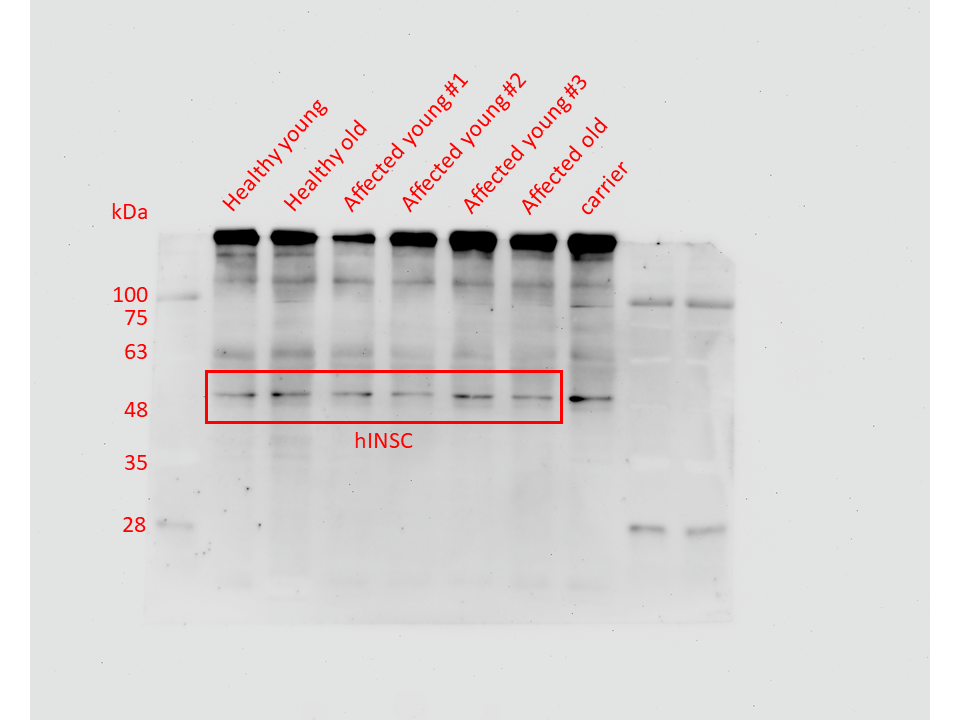

Supplement: Supplementary file 7 — Source data Fig. 4 [file 44321_2024_62_MOESM7_ESM.zip › Figure 4/Fig4B/Fig4B-hINSC.TIF]

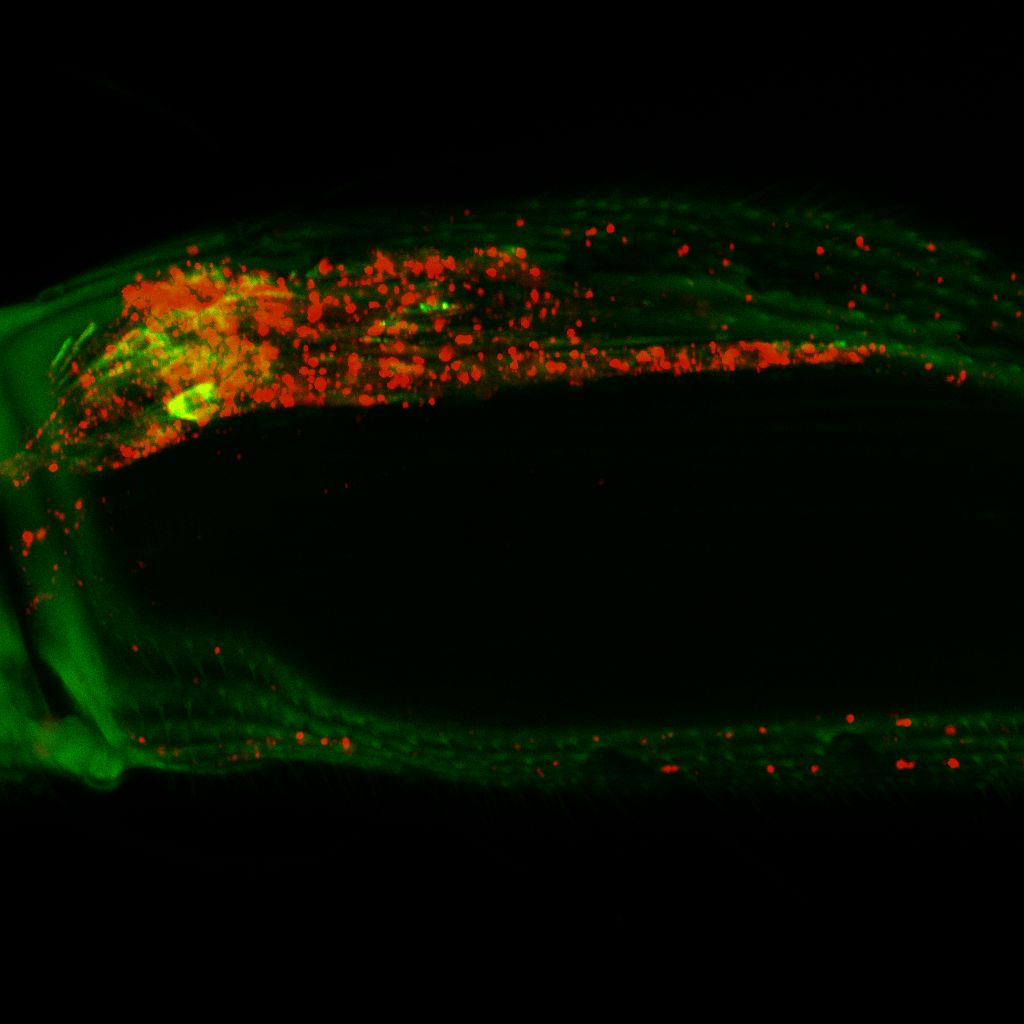

Supplement: Supplementary file 7 — Source data Fig. 4 [file 44321_2024_62_MOESM7_ESM.zip › Figure 4/Fig4C/Fig4C-W1-UAS-hINSC-M70R-Merge.tif]

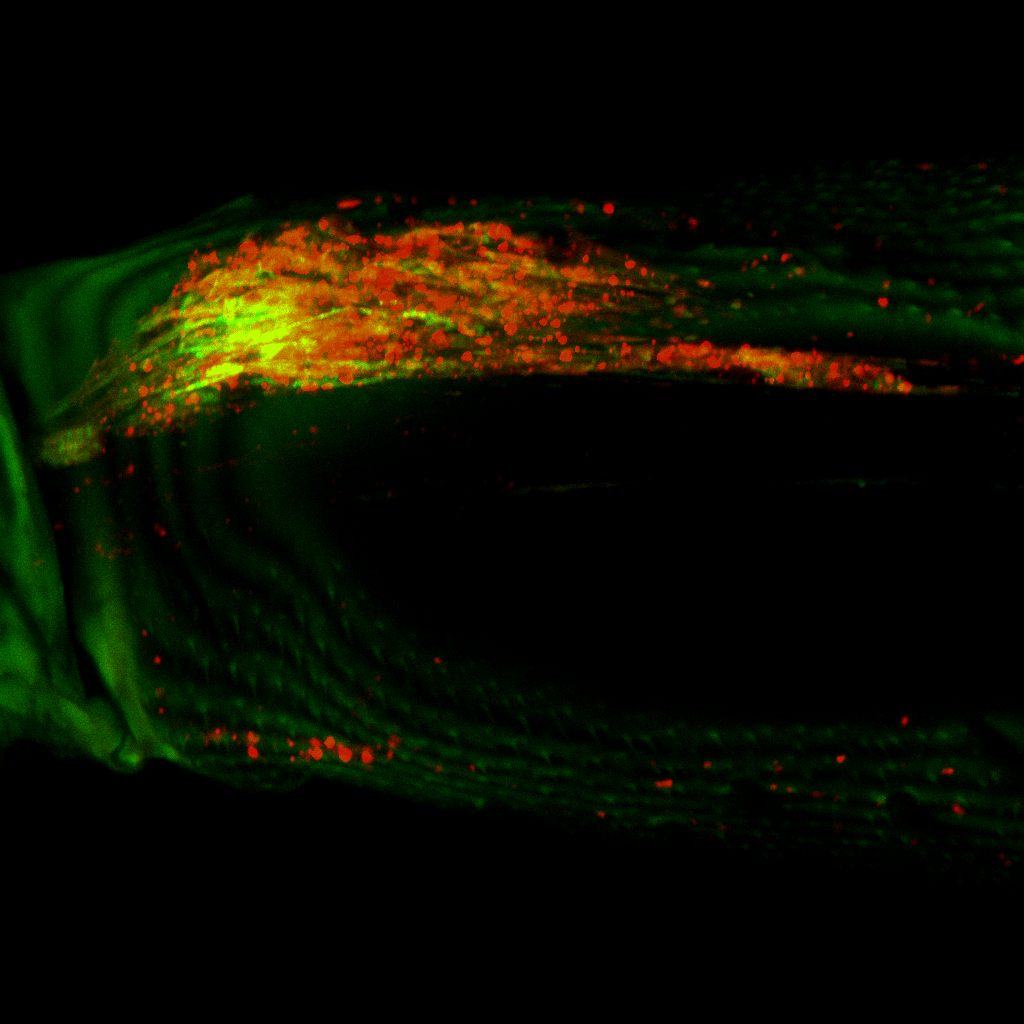

Supplement: Supplementary file 7 — Source data Fig. 4 [file 44321_2024_62_MOESM7_ESM.zip › Figure 4/Fig4C/Fig4C-W1-UAS-hINSC-WT-Merge.tif]

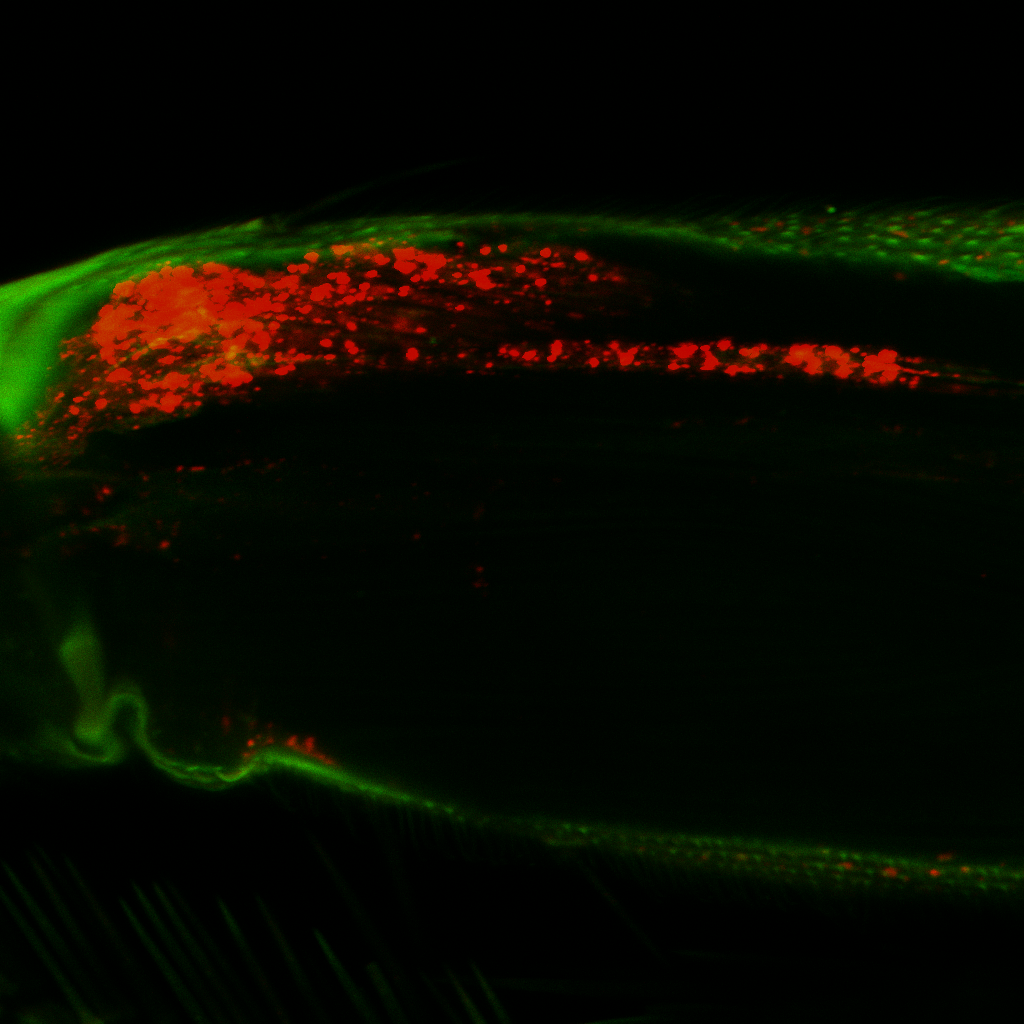

Supplement: Supplementary file 7 — Source data Fig. 4 [file 44321_2024_62_MOESM7_ESM.zip › Figure 4/Fig4C/Fig4C-W3-UAS-hINSC-M70R-Merge.tif]

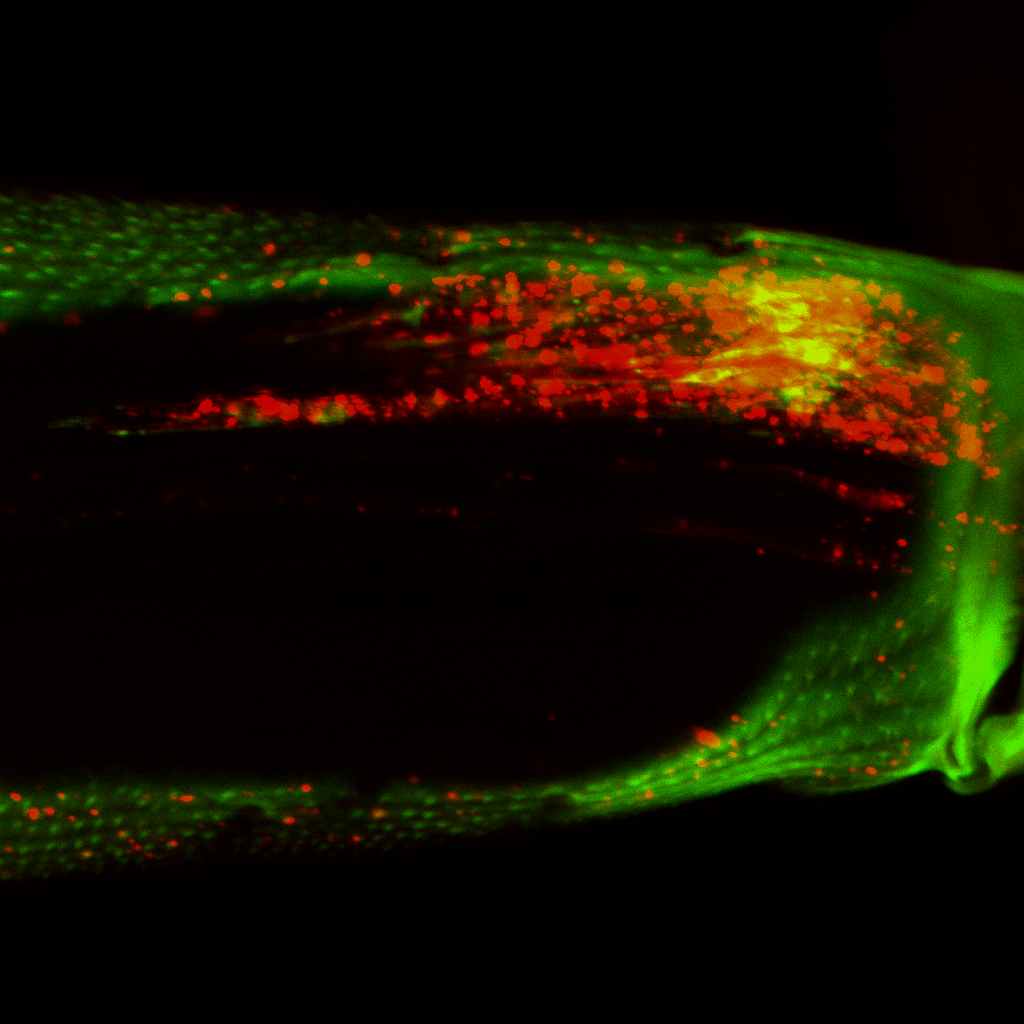

Supplement: Supplementary file 7 — Source data Fig. 4 [file 44321_2024_62_MOESM7_ESM.zip › Figure 4/Fig4C/Fig4C-W3-UAS-hINSC-WT-Merge.tif]

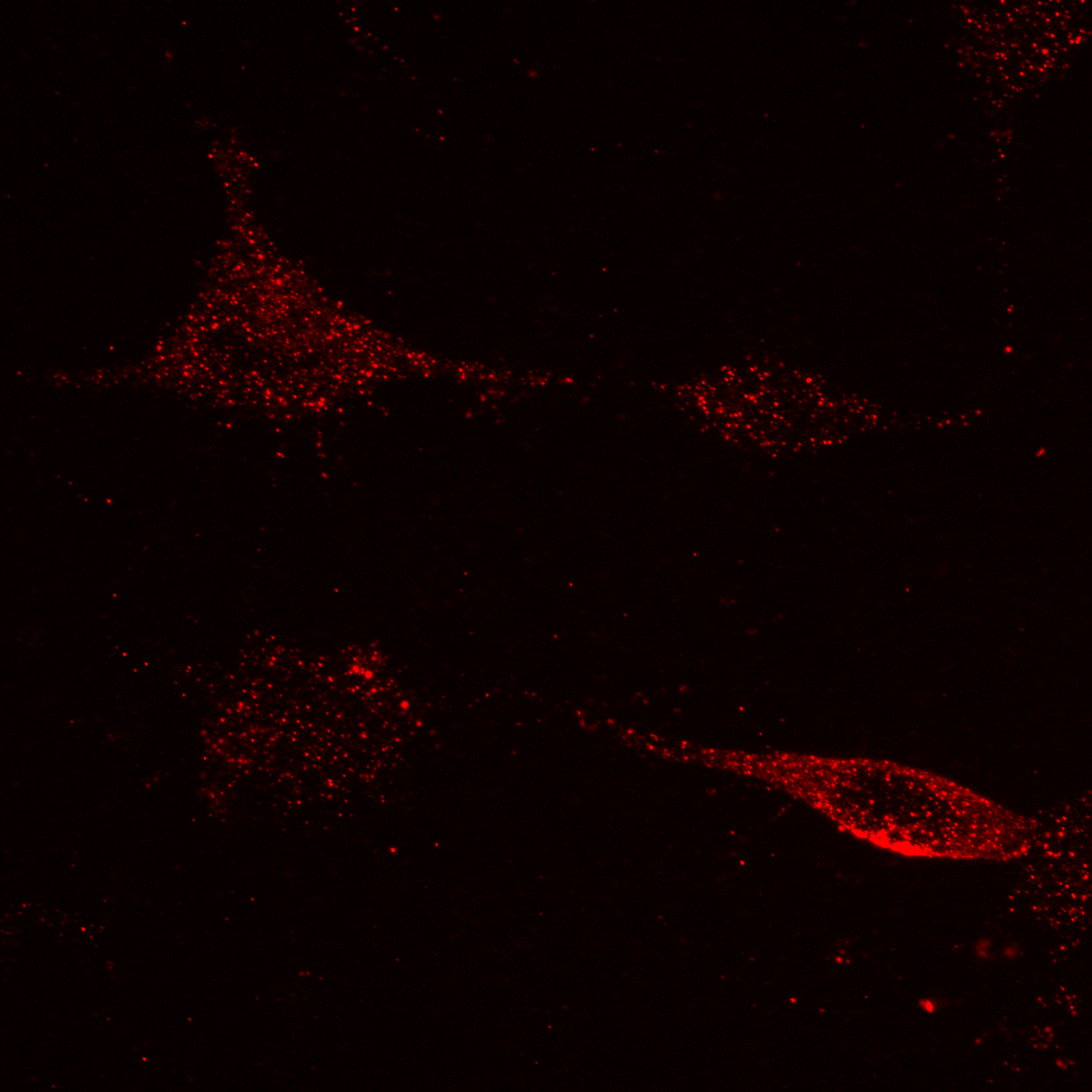

Supplement: Supplementary file 7 — Source data Fig. 4 [file 44321_2024_62_MOESM7_ESM.zip › Figure 4/Fig4G/Fig4G-hINSC-M70R&LGN-hINSC (red).tif]

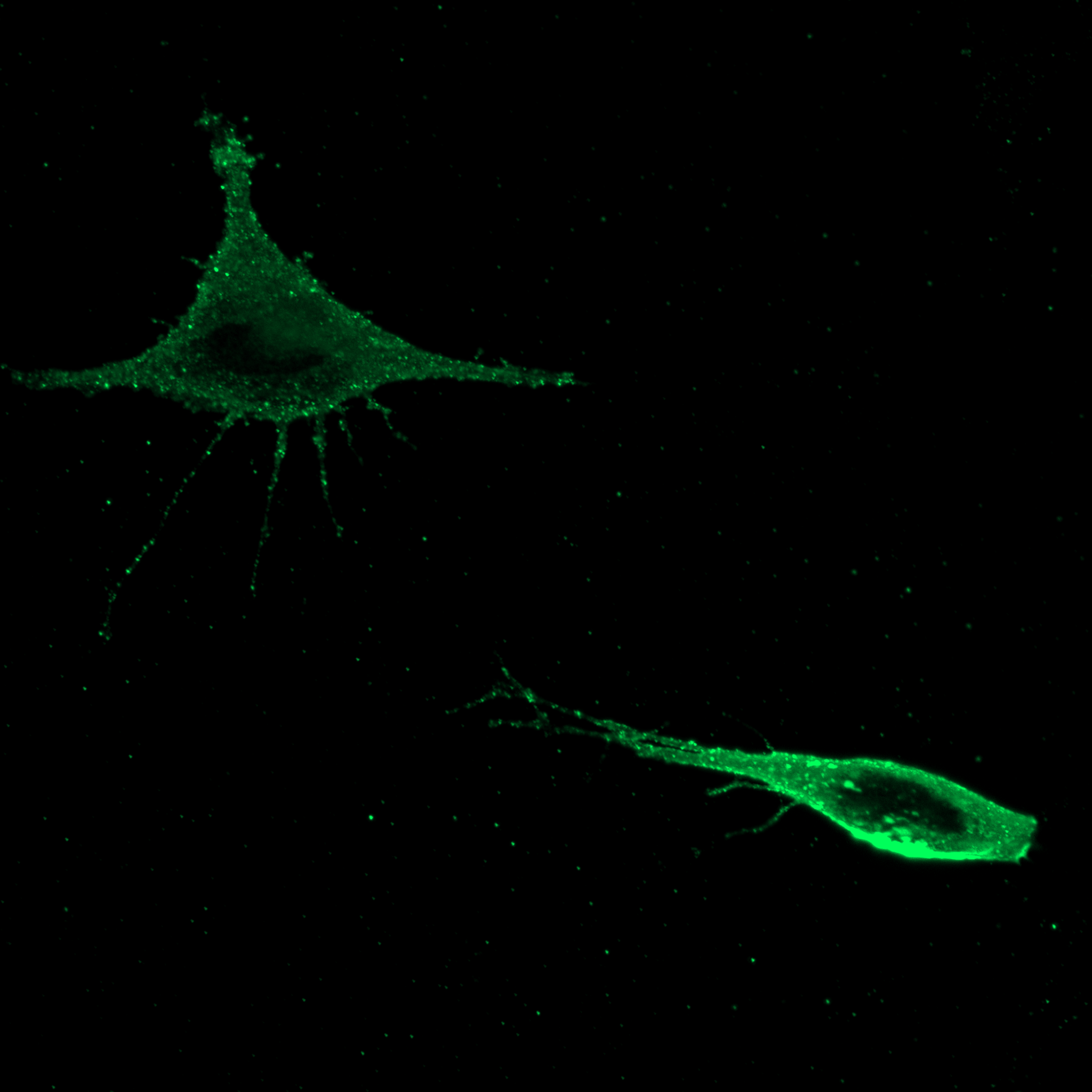

Supplement: Supplementary file 7 — Source data Fig. 4 [file 44321_2024_62_MOESM7_ESM.zip › Figure 4/Fig4G/Fig4G-hINSC-M70R&LGN-LGN (green).tif]

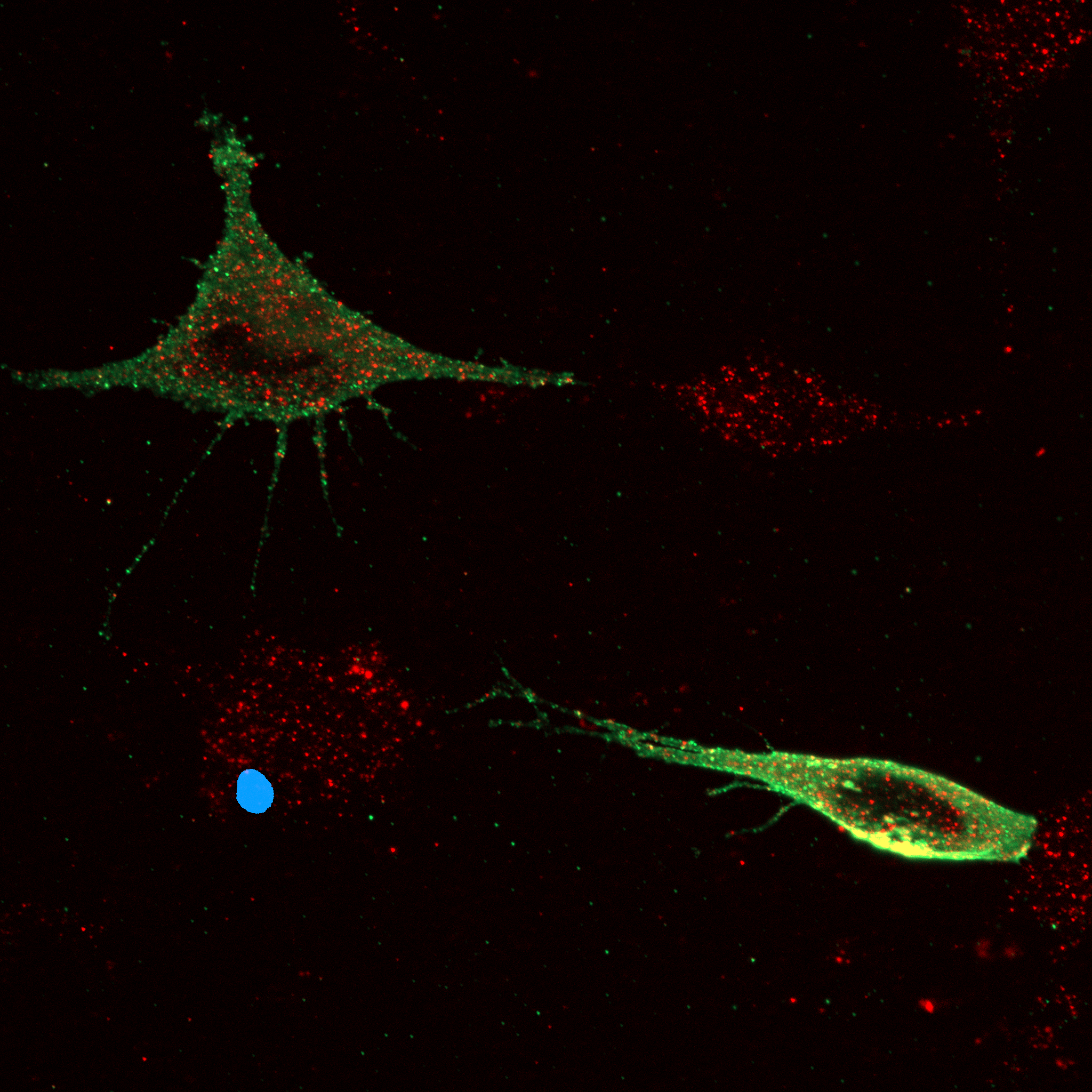

Supplement: Supplementary file 7 — Source data Fig. 4 [file 44321_2024_62_MOESM7_ESM.zip › Figure 4/Fig4G/Fig4G-hINSC-M70R&LGN-Merge.tif]

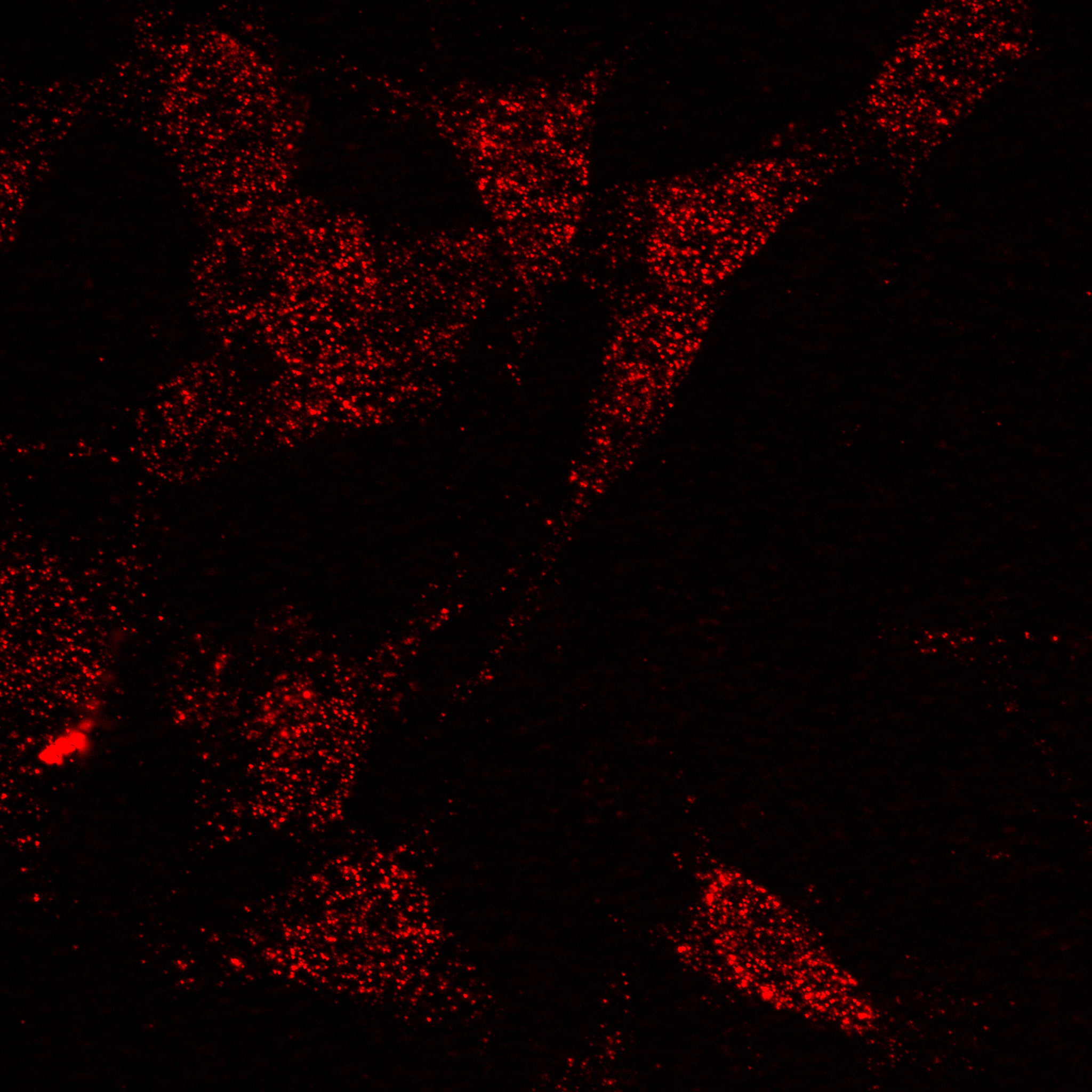

Supplement: Supplementary file 7 — Source data Fig. 4 [file 44321_2024_62_MOESM7_ESM.zip › Figure 4/Fig4G/Fig4G-hINSC-WT&LGN-hINSC (red).tif]

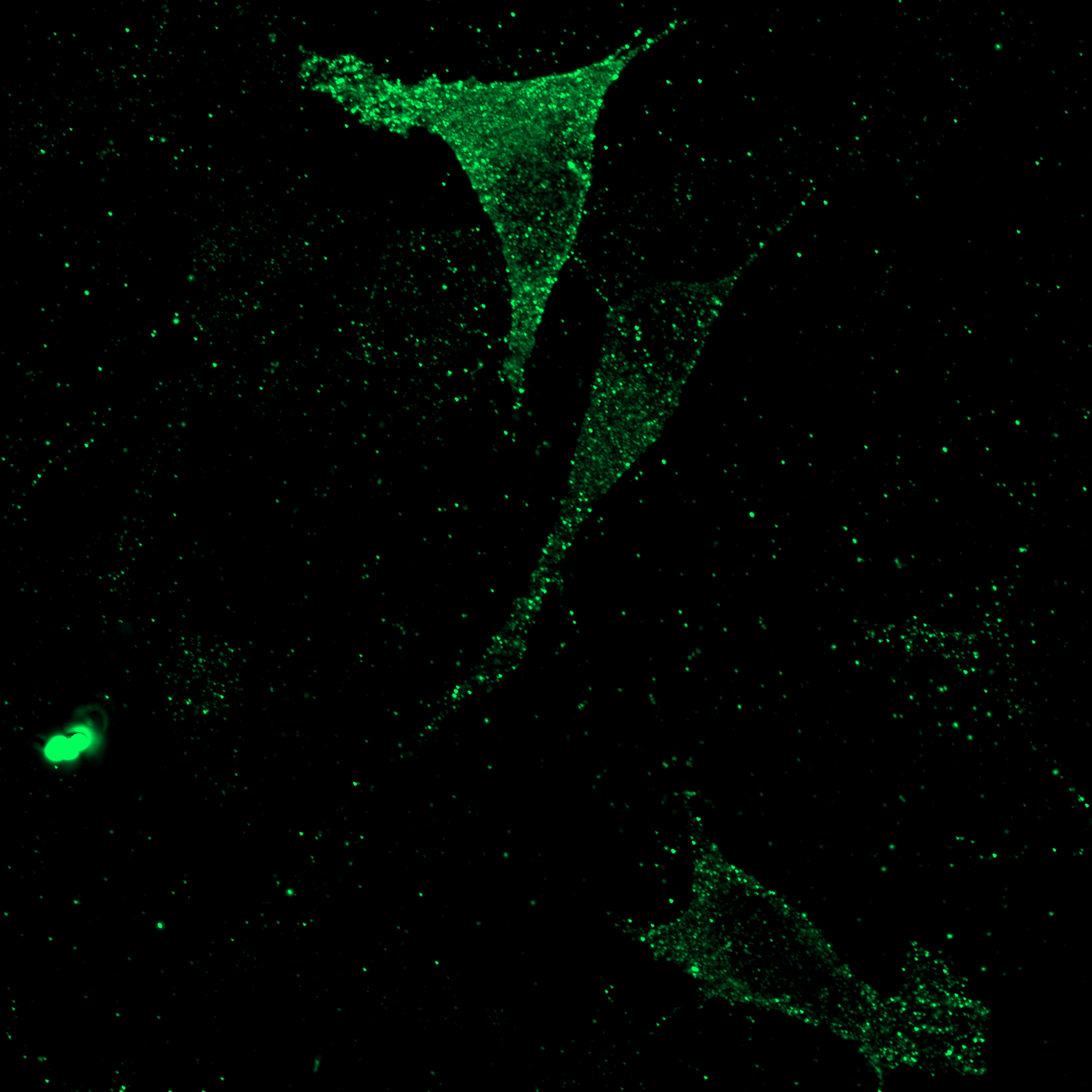

Supplement: Supplementary file 7 — Source data Fig. 4 [file 44321_2024_62_MOESM7_ESM.zip › Figure 4/Fig4G/Fig4G-hINSC-WT&LGN-LGN (green).tif]

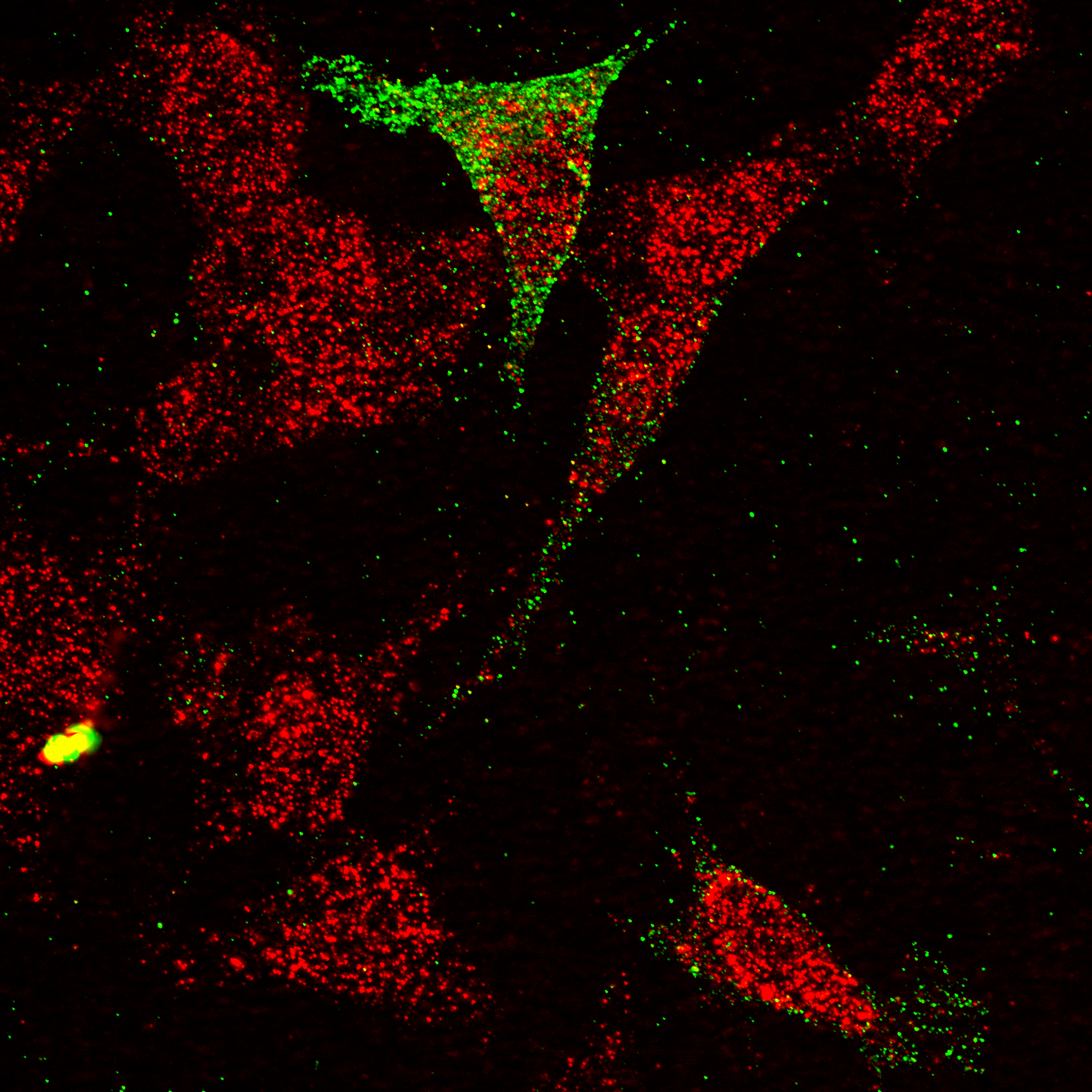

Supplement: Supplementary file 7 — Source data Fig. 4 [file 44321_2024_62_MOESM7_ESM.zip › Figure 4/Fig4G/Fig4G-hINSC-WT&LGN-Merge.tif]

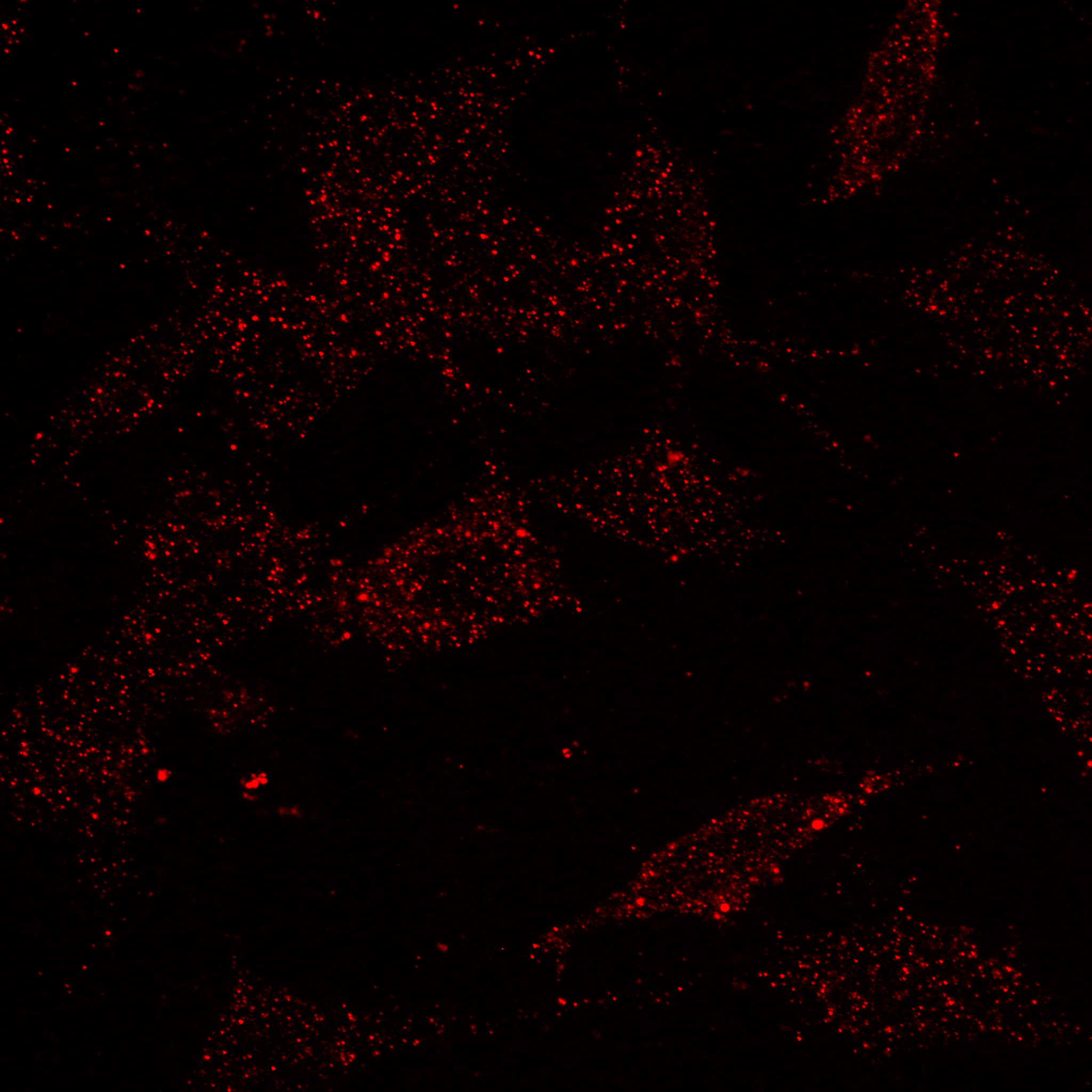

Supplement: Supplementary file 7 — Source data Fig. 4 [file 44321_2024_62_MOESM7_ESM.zip › Figure 4/Fig4I/Fig4I-hINSC-M70R&PAR3-hINSC (red).tif]

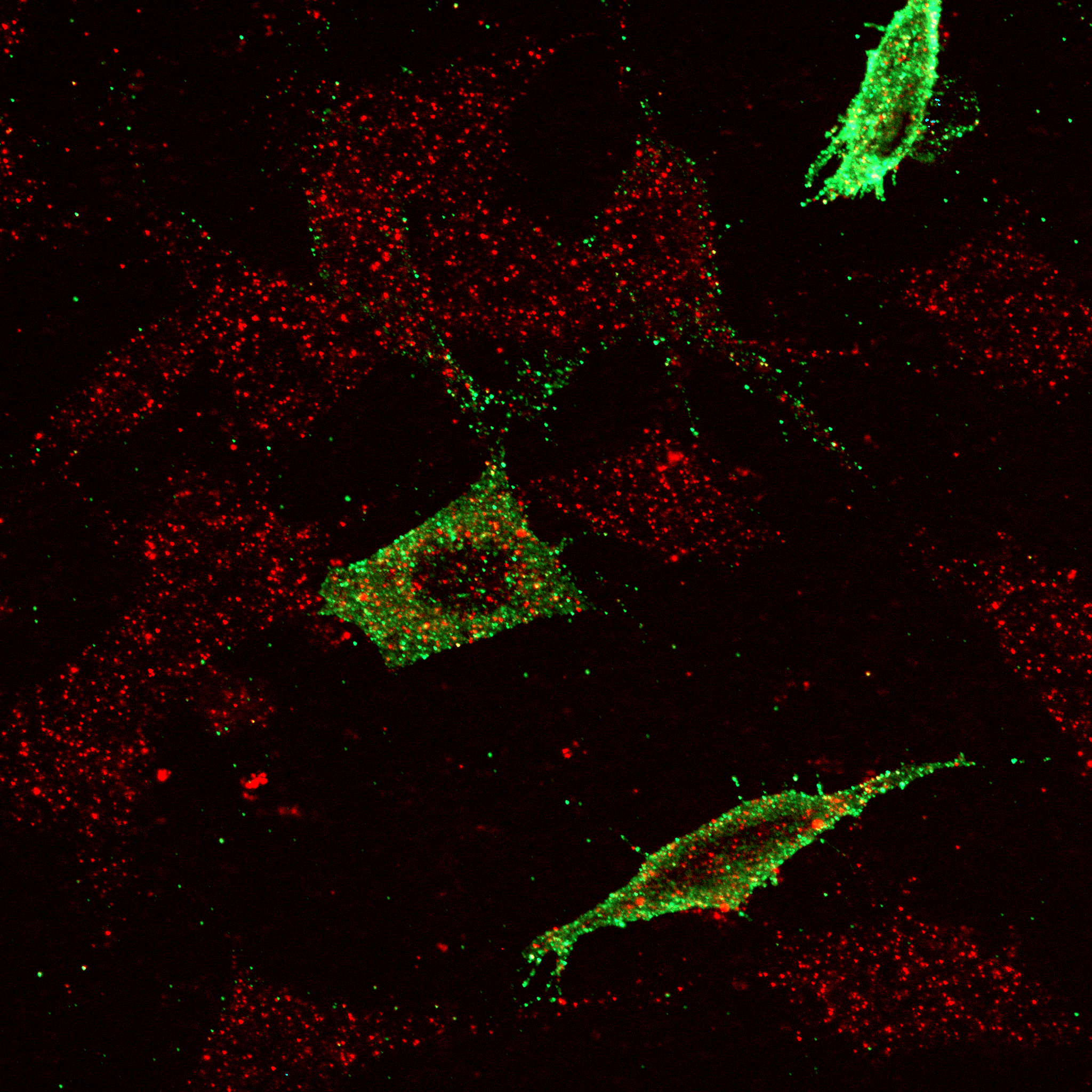

Supplement: Supplementary file 7 — Source data Fig. 4 [file 44321_2024_62_MOESM7_ESM.zip › Figure 4/Fig4I/Fig4I-hINSC-M70R&PAR3-Merge.tif]

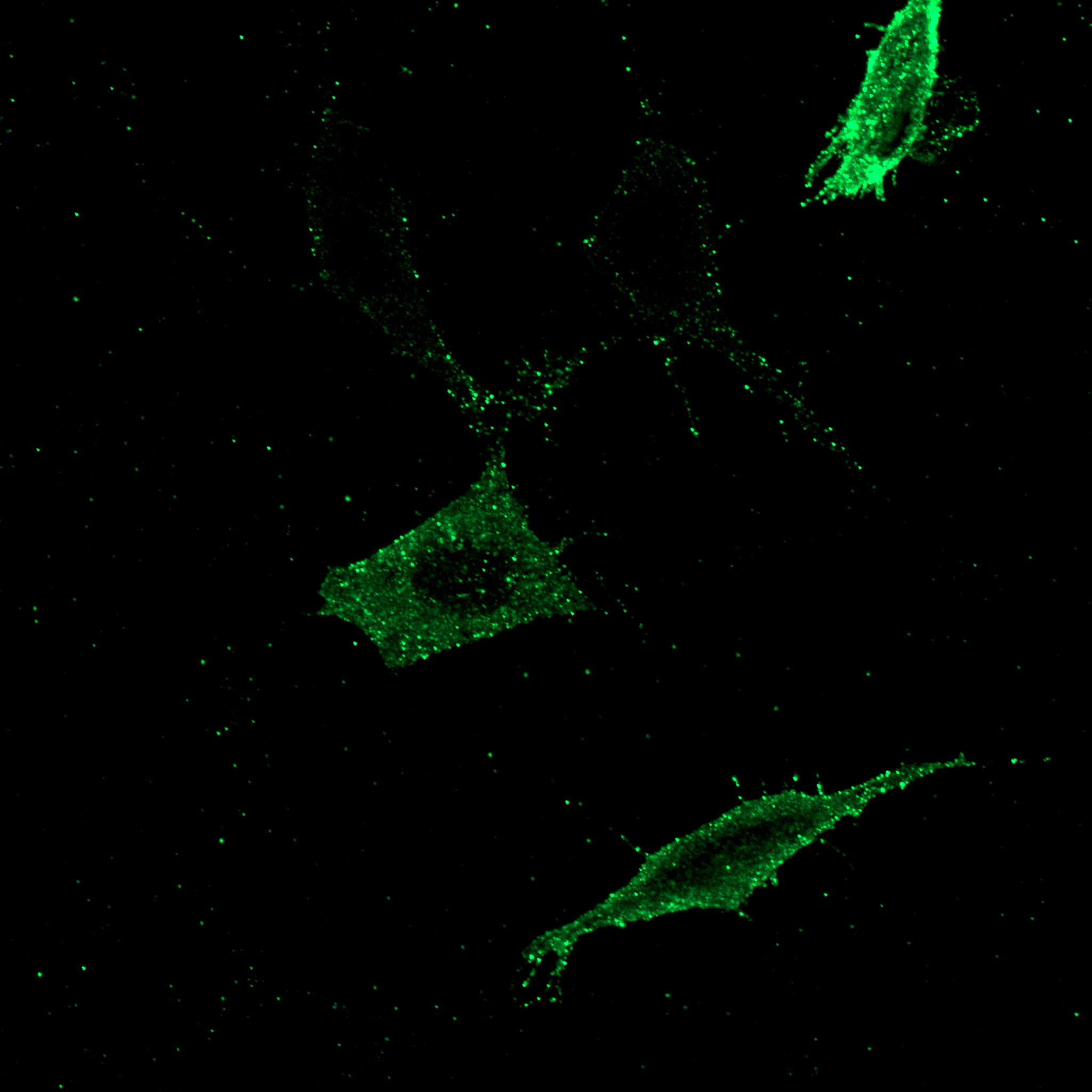

Supplement: Supplementary file 7 — Source data Fig. 4 [file 44321_2024_62_MOESM7_ESM.zip › Figure 4/Fig4I/Fig4I-hINSC-M70R&PAR3-PAR3 (green).tif]

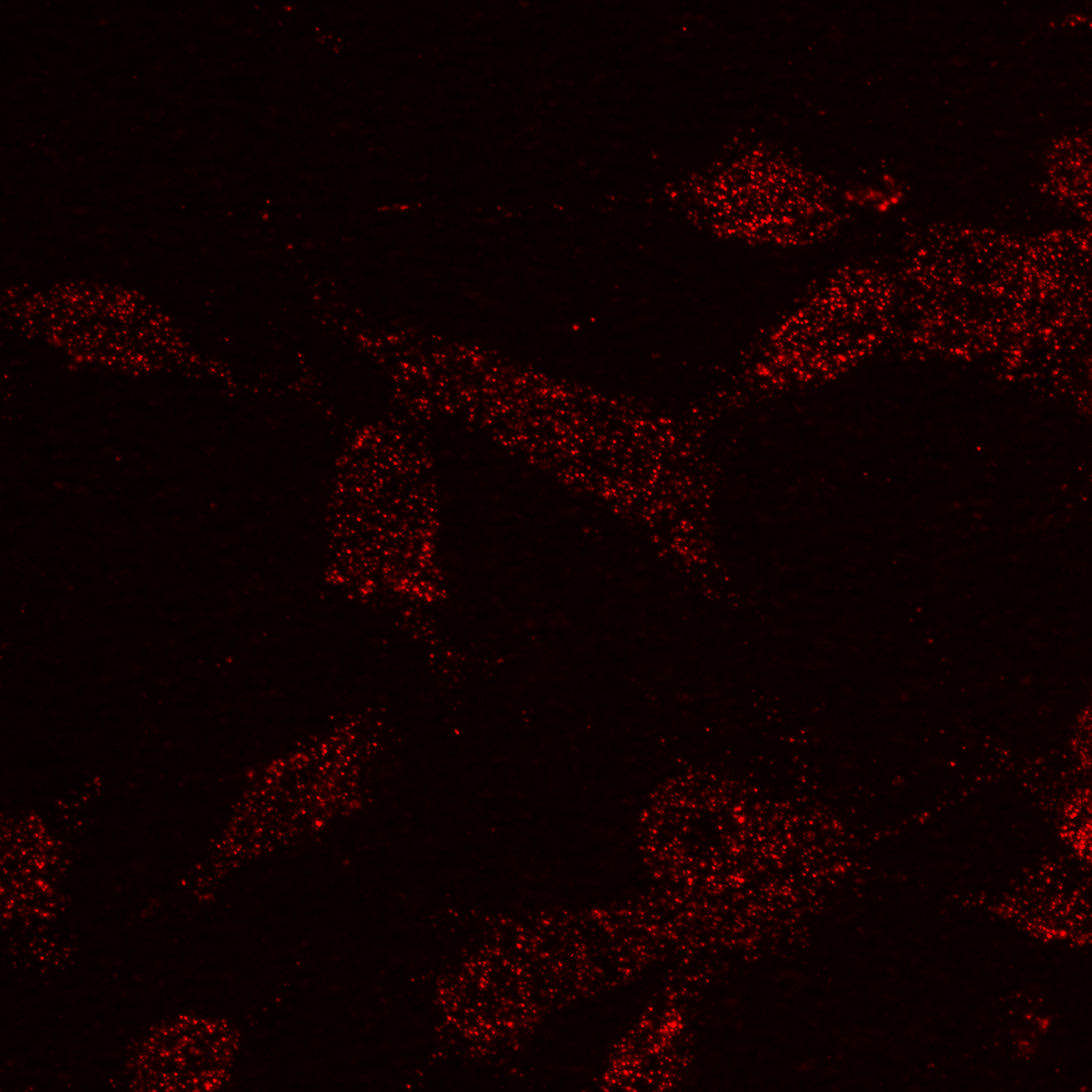

Supplement: Supplementary file 7 — Source data Fig. 4 [file 44321_2024_62_MOESM7_ESM.zip › Figure 4/Fig4I/Fig4I-hINSC-WT&PAR3-hINSC (red).tif]

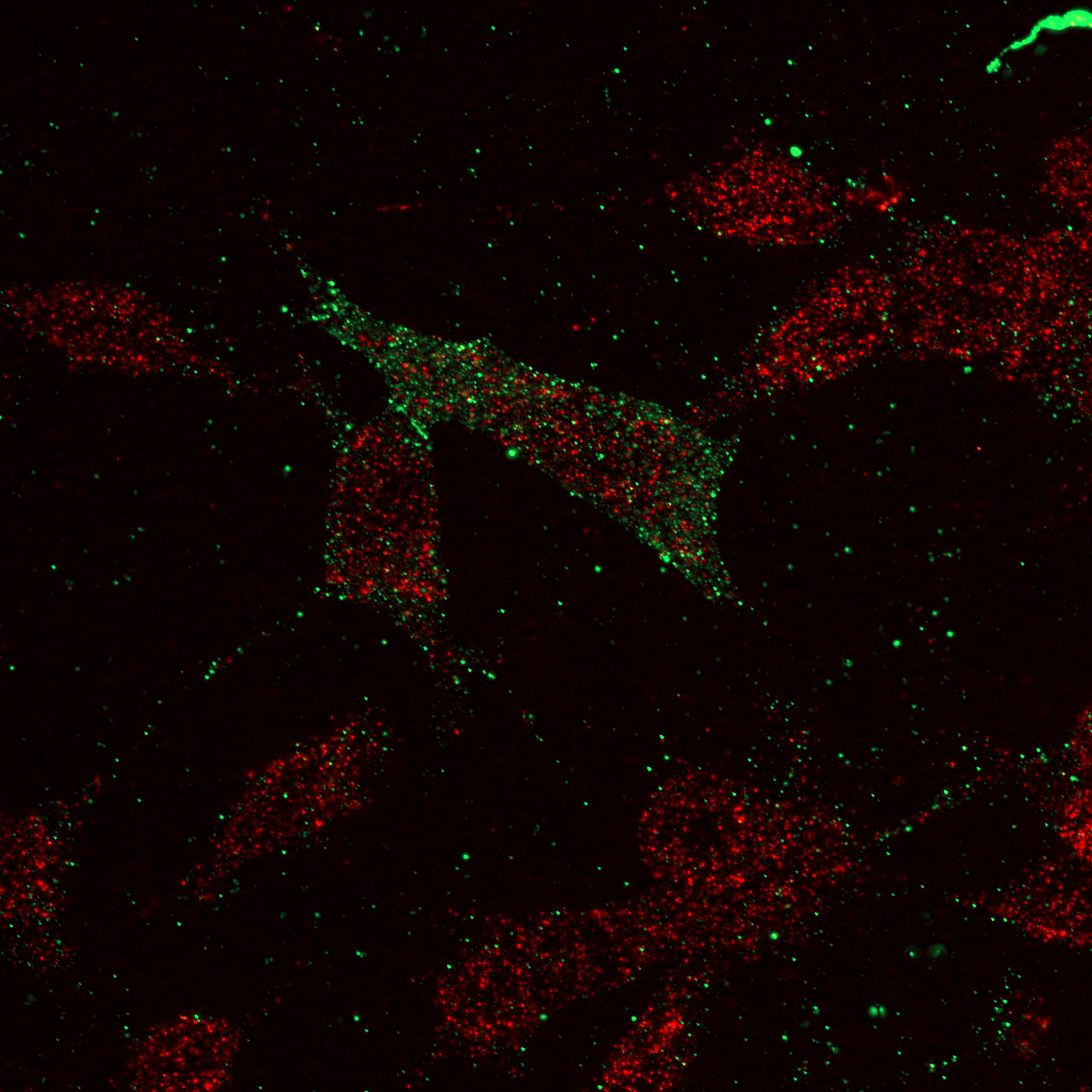

Supplement: Supplementary file 7 — Source data Fig. 4 [file 44321_2024_62_MOESM7_ESM.zip › Figure 4/Fig4I/Fig4I-hINSC-WT&PAR3-Merge.tif]

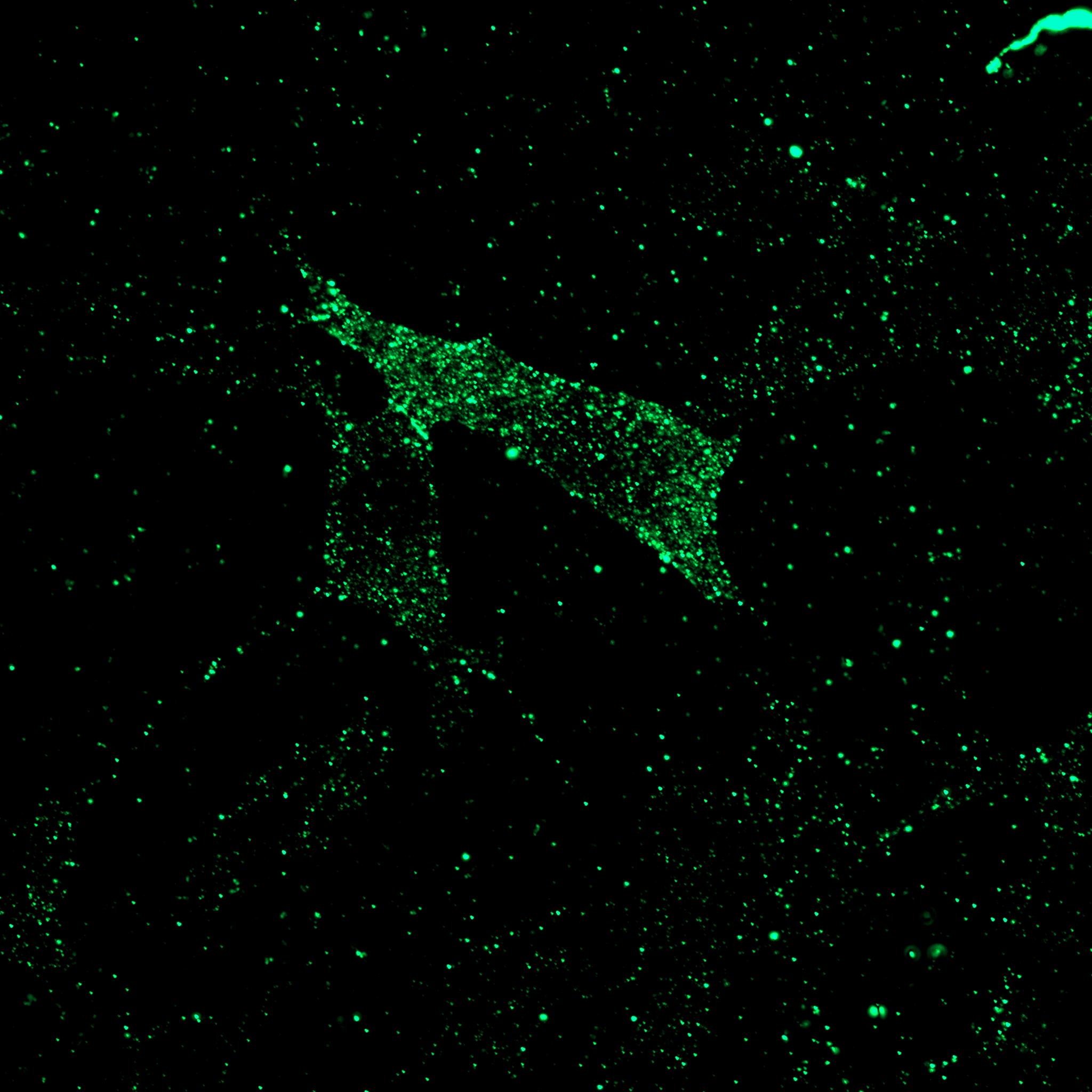

Supplement: Supplementary file 7 — Source data Fig. 4 [file 44321_2024_62_MOESM7_ESM.zip › Figure 4/Fig4I/Fig4I-hINSC-WT&PAR3-PAR3 (green).tif]

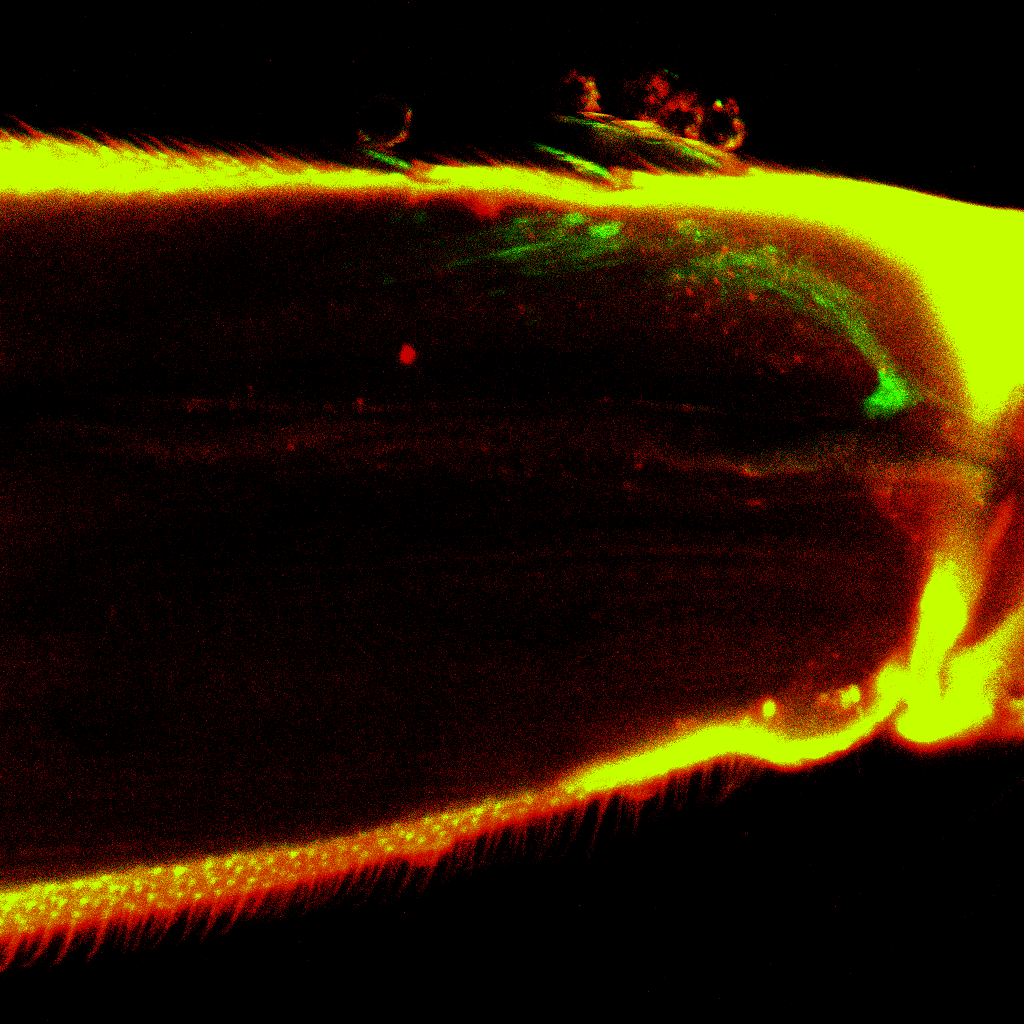

Supplement: Supplementary file 7 — Source data Fig. 4 [file 44321_2024_62_MOESM7_ESM.zip › Figure 4/Fig4K/Fig4K-UAS-hINSC-M70R.tif]

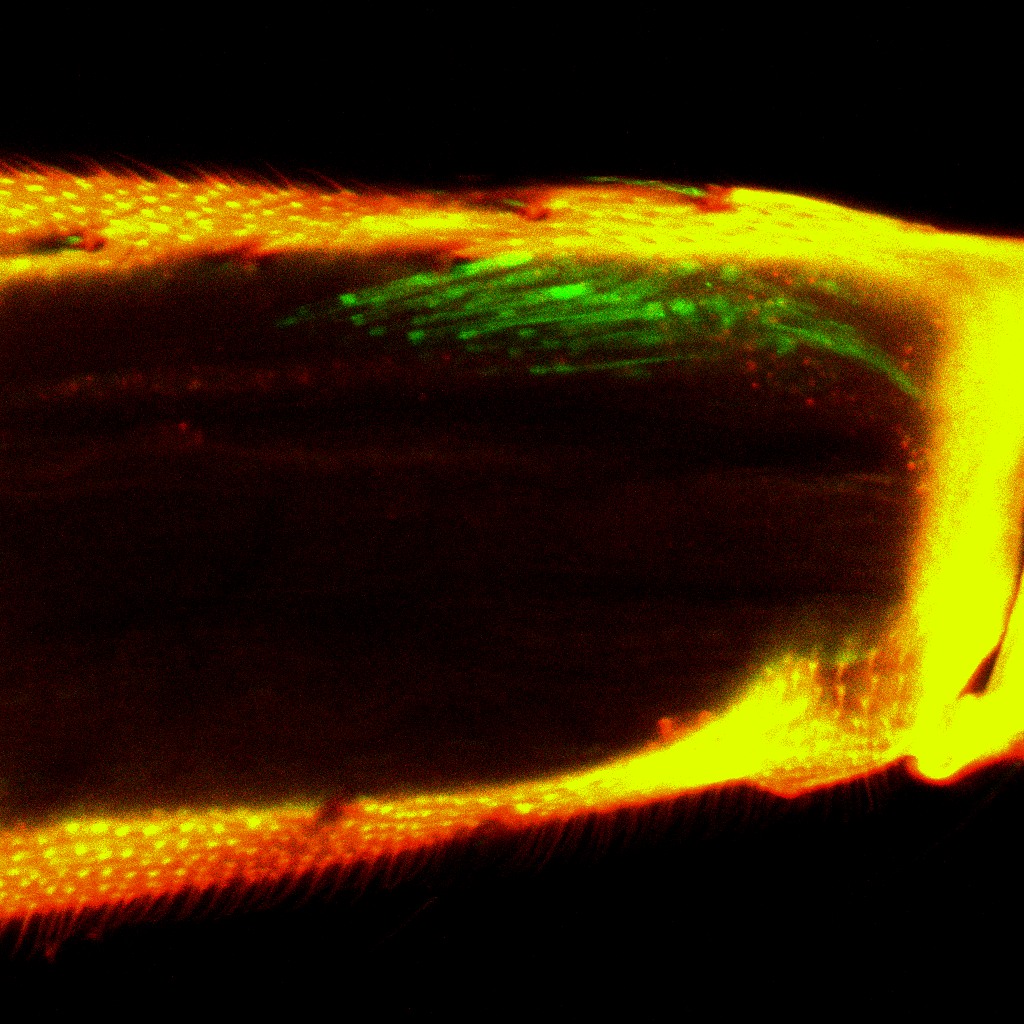

Supplement: Supplementary file 7 — Source data Fig. 4 [file 44321_2024_62_MOESM7_ESM.zip › Figure 4/Fig4K/Fig4K-UAS-hINSC-WT.tif]

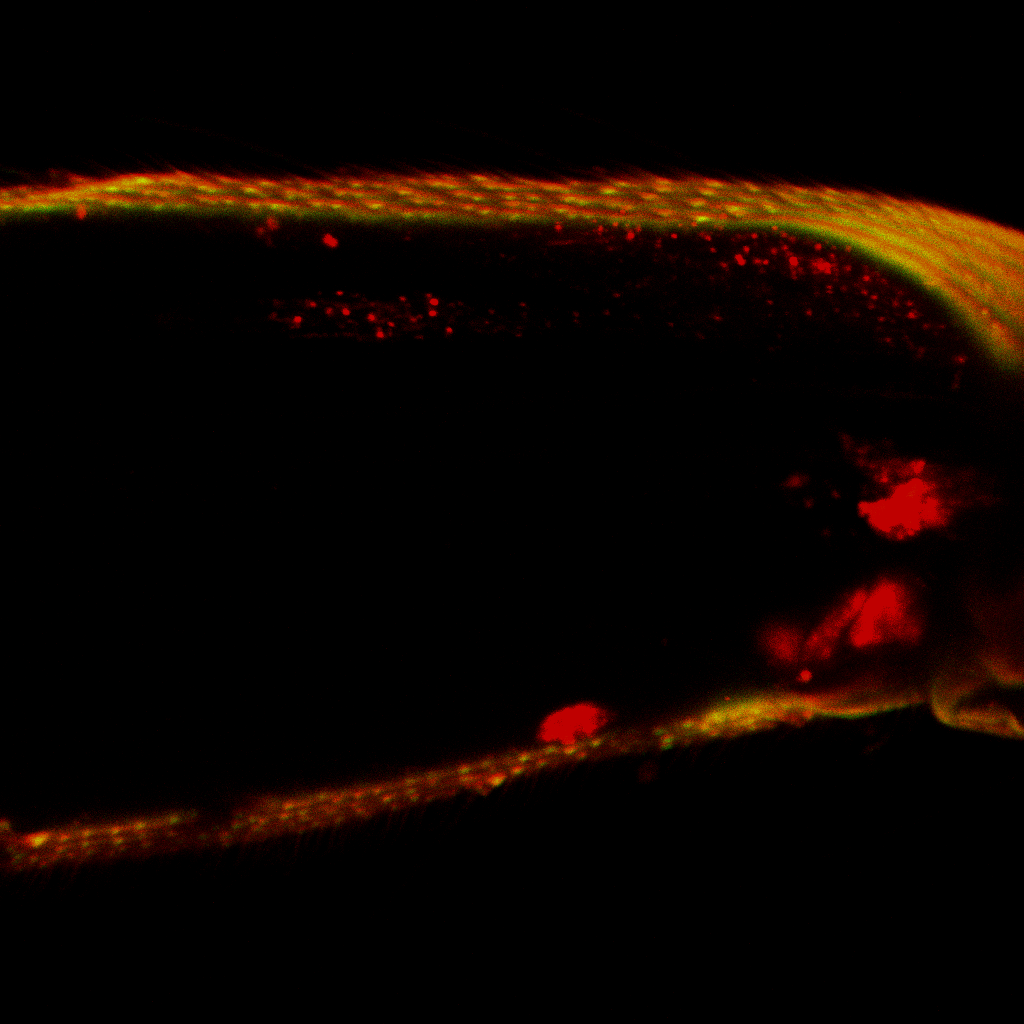

Supplement: Supplementary file 8 — Source data Fig. 5 [file 44321_2024_62_MOESM8_ESM.zip › Figure 5/Fig5A/Fig5A-UAS-dInsc-RNAi&UAS-hINSC-M70R-Merge.tif]

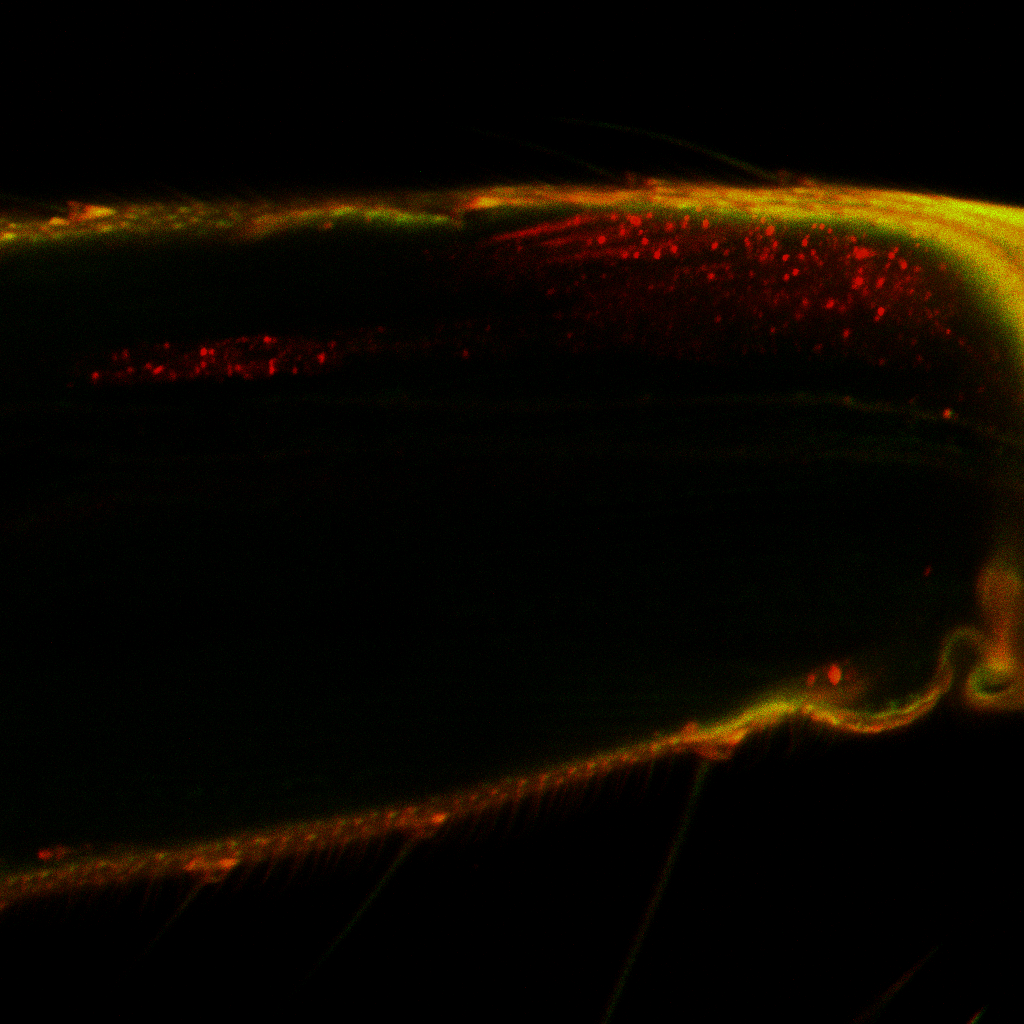

Supplement: Supplementary file 8 — Source data Fig. 5 [file 44321_2024_62_MOESM8_ESM.zip › Figure 5/Fig5A/Fig5A-UAS-dInsc-RNAi&UAS-hINSC-WT-Merge.tif]

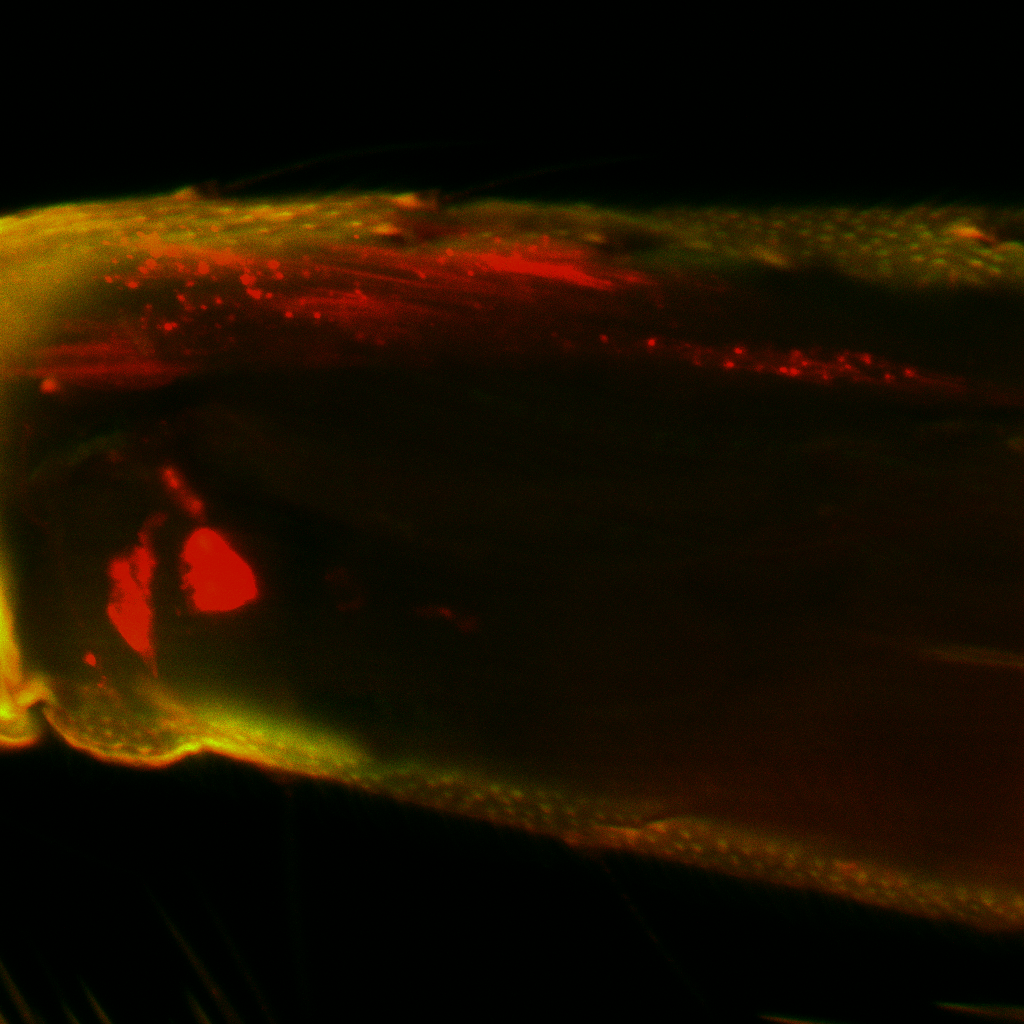

Supplement: Supplementary file 8 — Source data Fig. 5 [file 44321_2024_62_MOESM8_ESM.zip › Figure 5/Fig5A/Fig5A-UAS-dInsc-RNAi-Merge.tif]

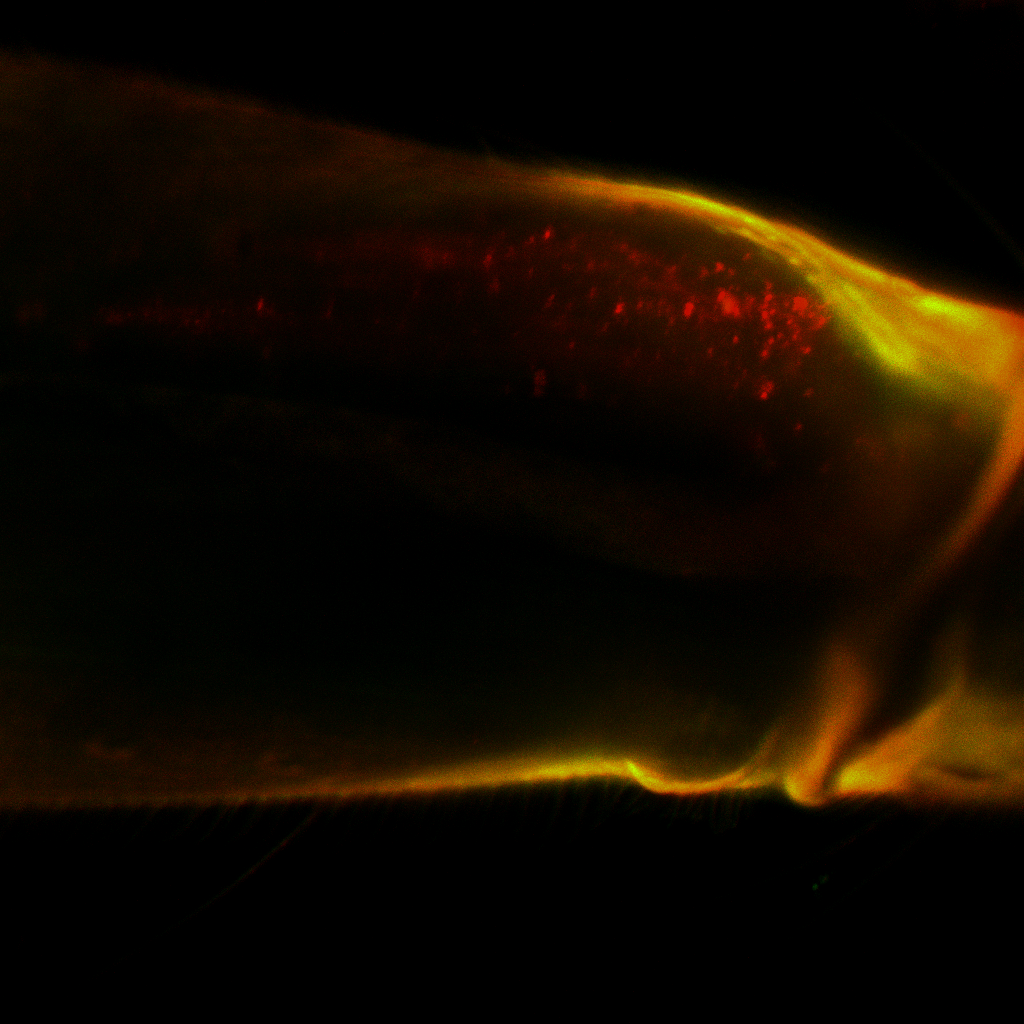

Supplement: Supplementary file 8 — Source data Fig. 5 [file 44321_2024_62_MOESM8_ESM.zip › Figure 5/Fig5A/Fig5A-UAS-w-RNAi-Merge.tif]

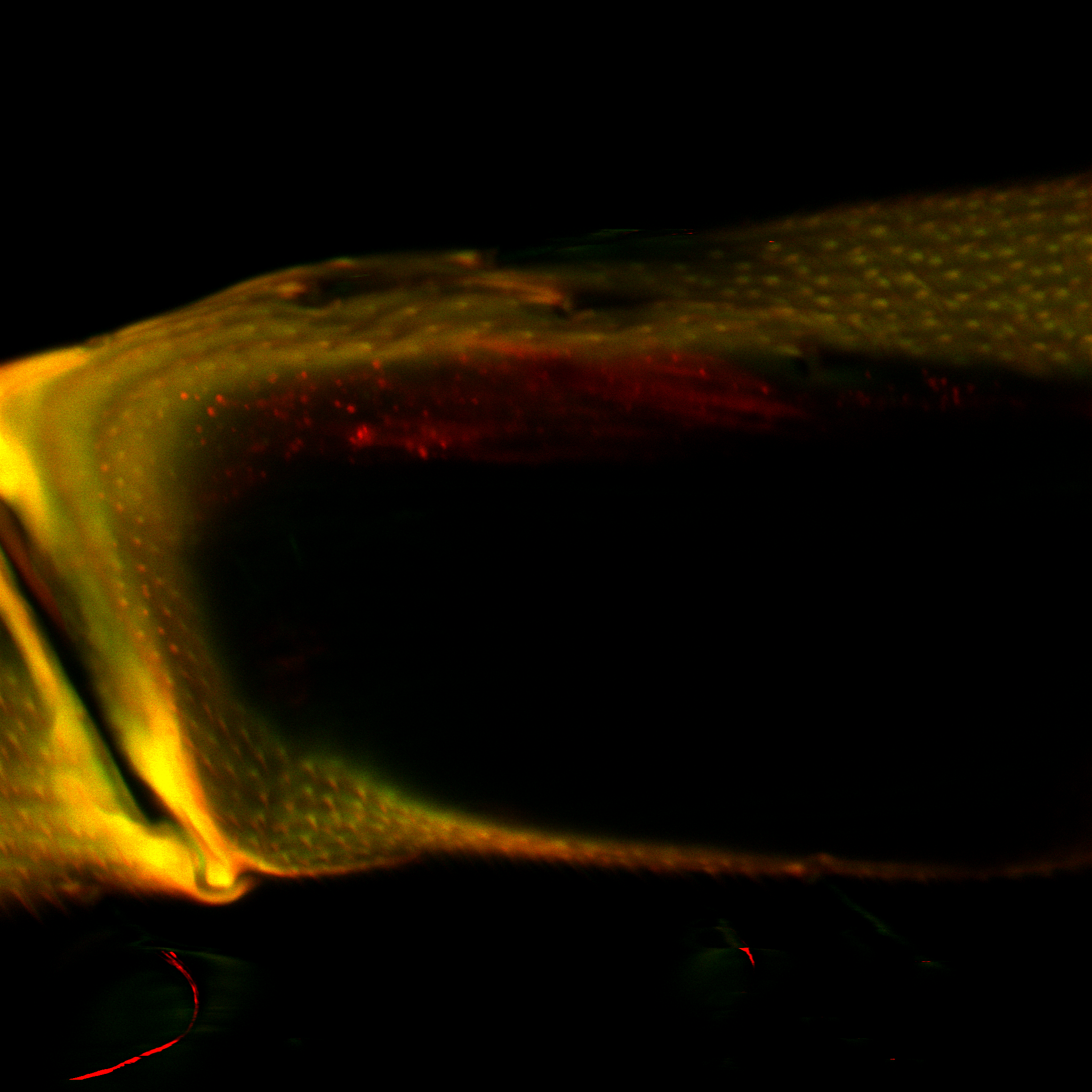

Supplement: Supplementary file 9 — Source data Fig. 6 [file 44321_2024_62_MOESM9_ESM.zip › Figure 6/Fig6A/Fig6A-KM-DMSO-Merge.tif]

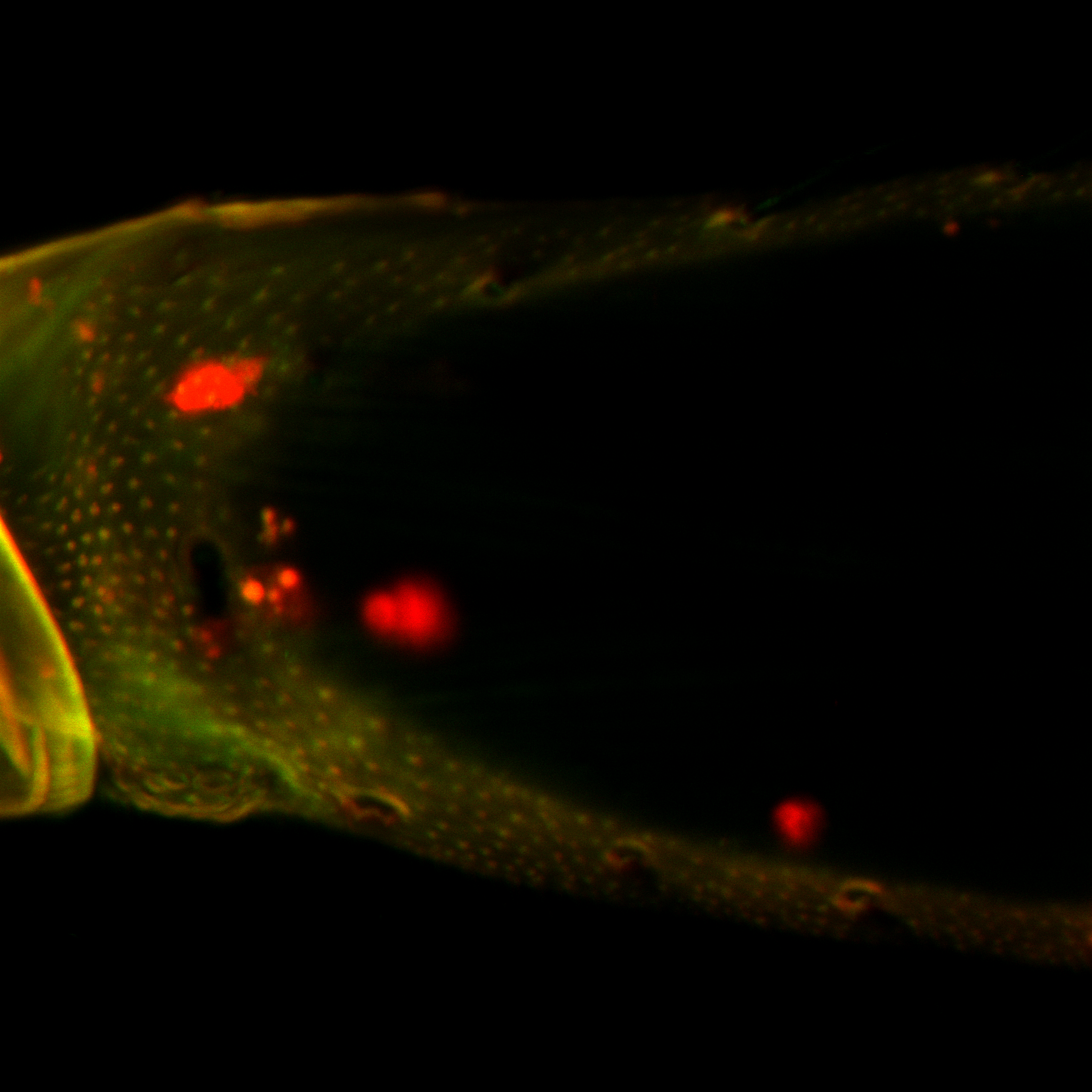

Supplement: Supplementary file 9 — Source data Fig. 6 [file 44321_2024_62_MOESM9_ESM.zip › Figure 6/Fig6A/Fig6A-KM-DMSO-Taxol.tif]

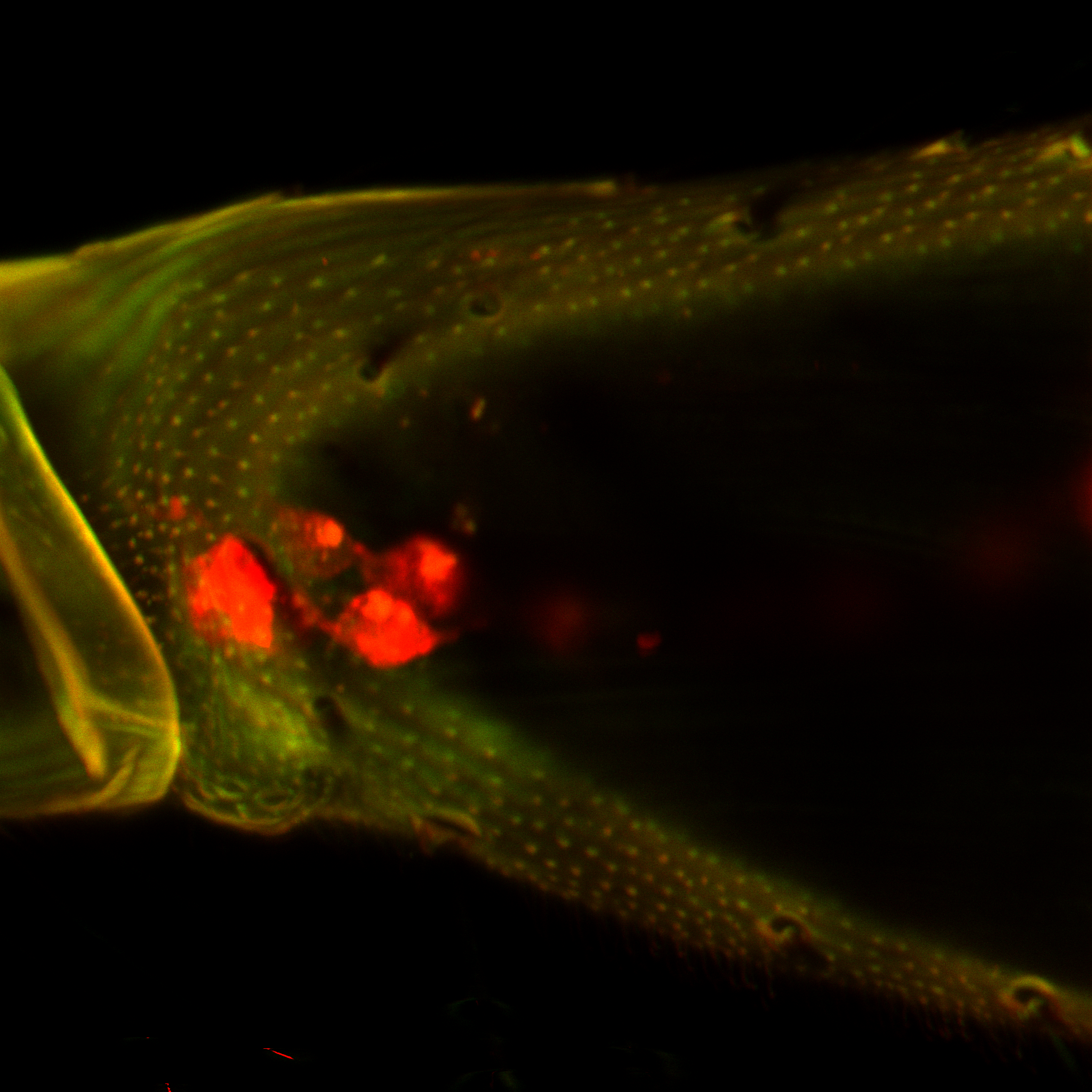

Supplement: Supplementary file 9 — Source data Fig. 6 [file 44321_2024_62_MOESM9_ESM.zip › Figure 6/Fig6A/Fig6A-KR-DMSO-Merge.tif]

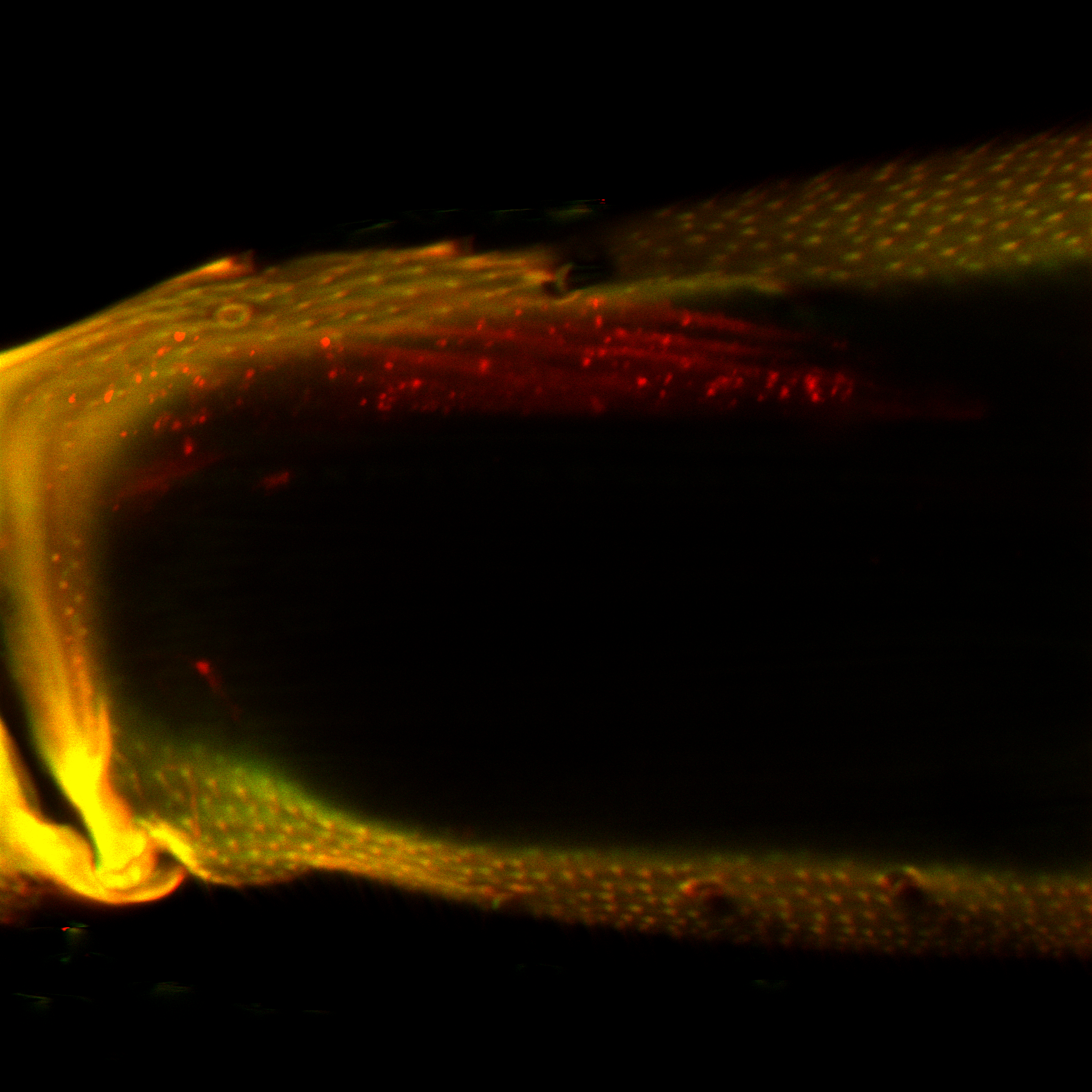

Supplement: Supplementary file 9 — Source data Fig. 6 [file 44321_2024_62_MOESM9_ESM.zip › Figure 6/Fig6A/Fig6A-KR-Taxol-Merge.tif]

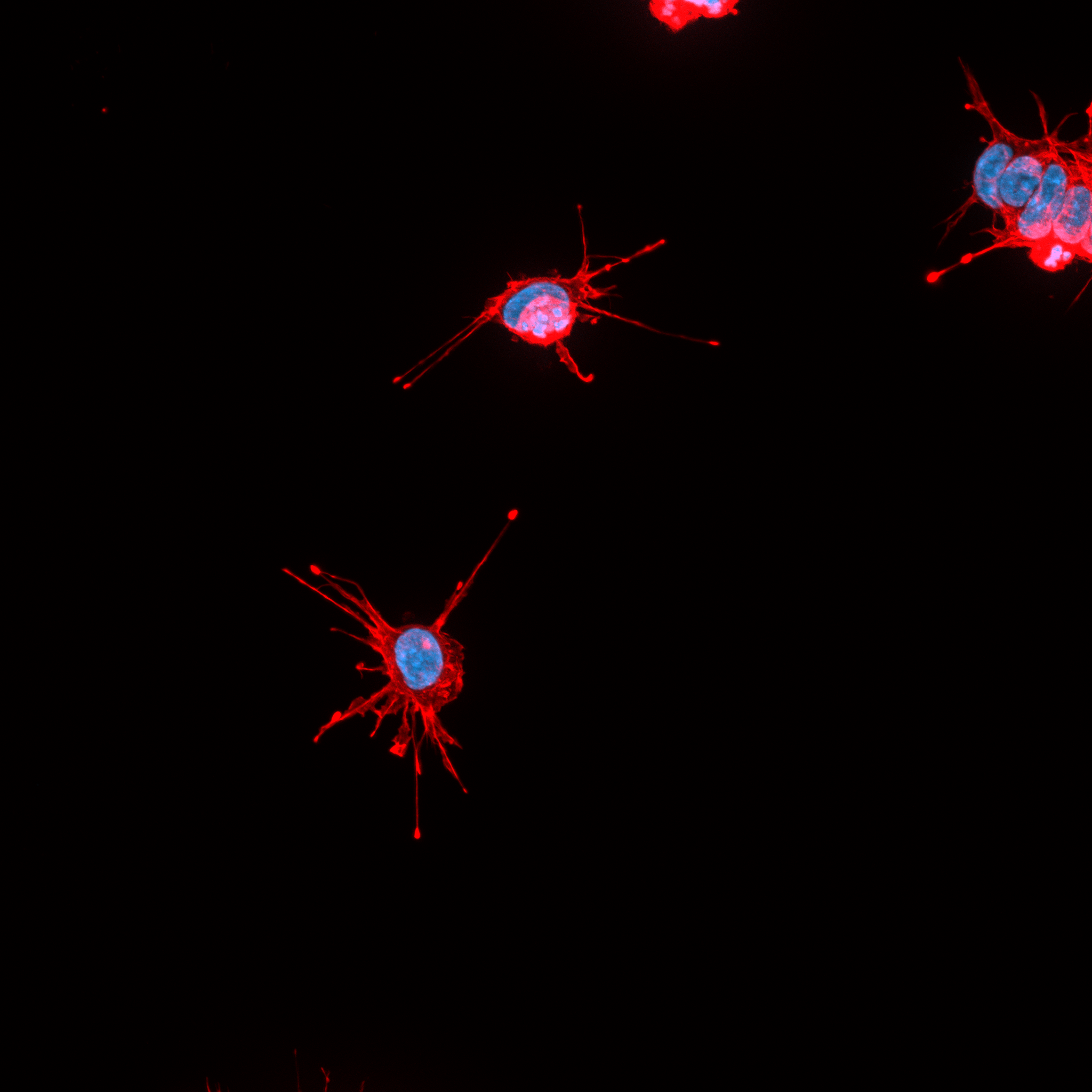

Supplement: Supplementary file 9 — Source data Fig. 6 [file 44321_2024_62_MOESM9_ESM.zip › Figure 6/Fig6E/Fig6E-INSC shRNA-500nM Taxol-Merge.tif]

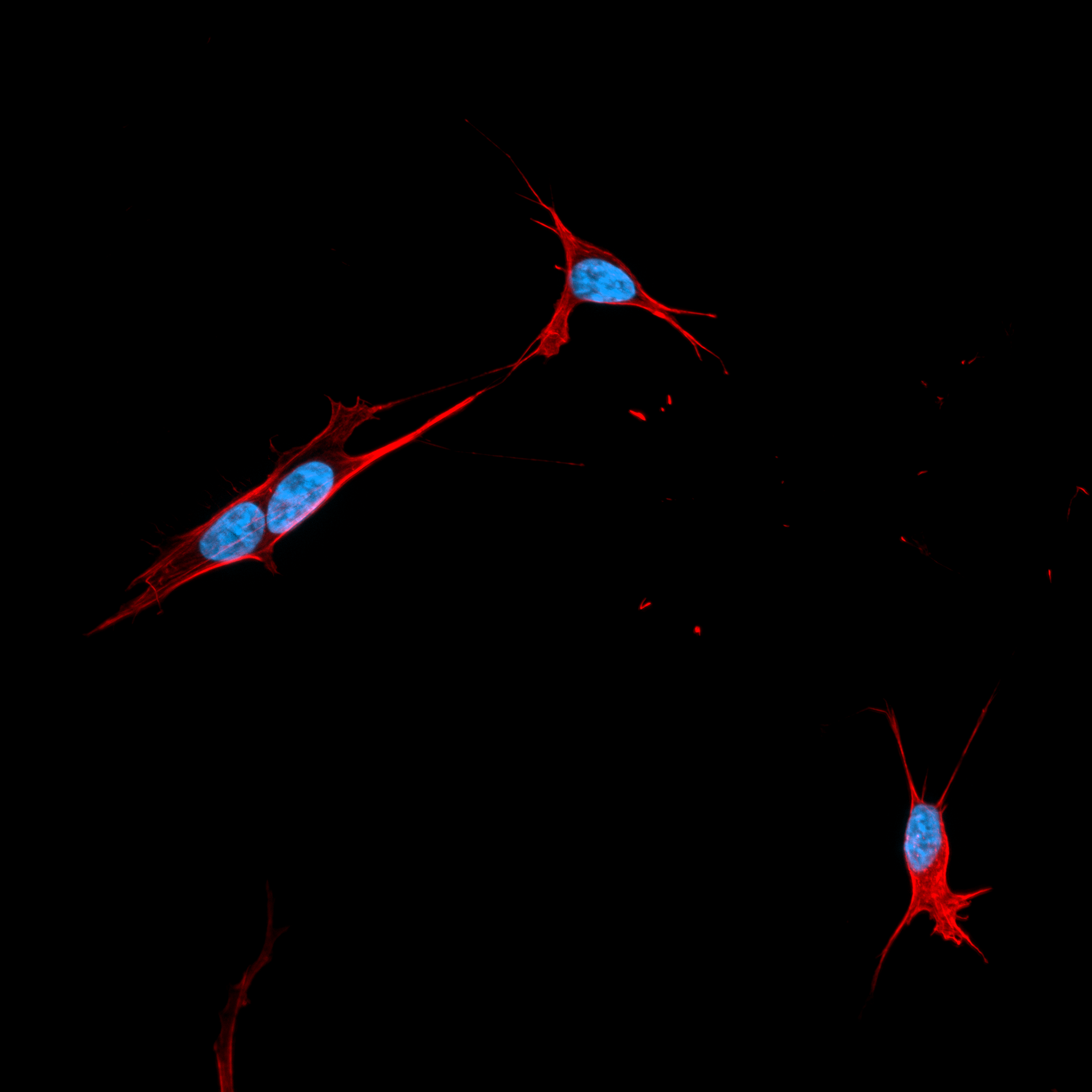

Supplement: Supplementary file 9 — Source data Fig. 6 [file 44321_2024_62_MOESM9_ESM.zip › Figure 6/Fig6E/Fig6E-INSC shRNA-50nM Taxol-Merge.tif]

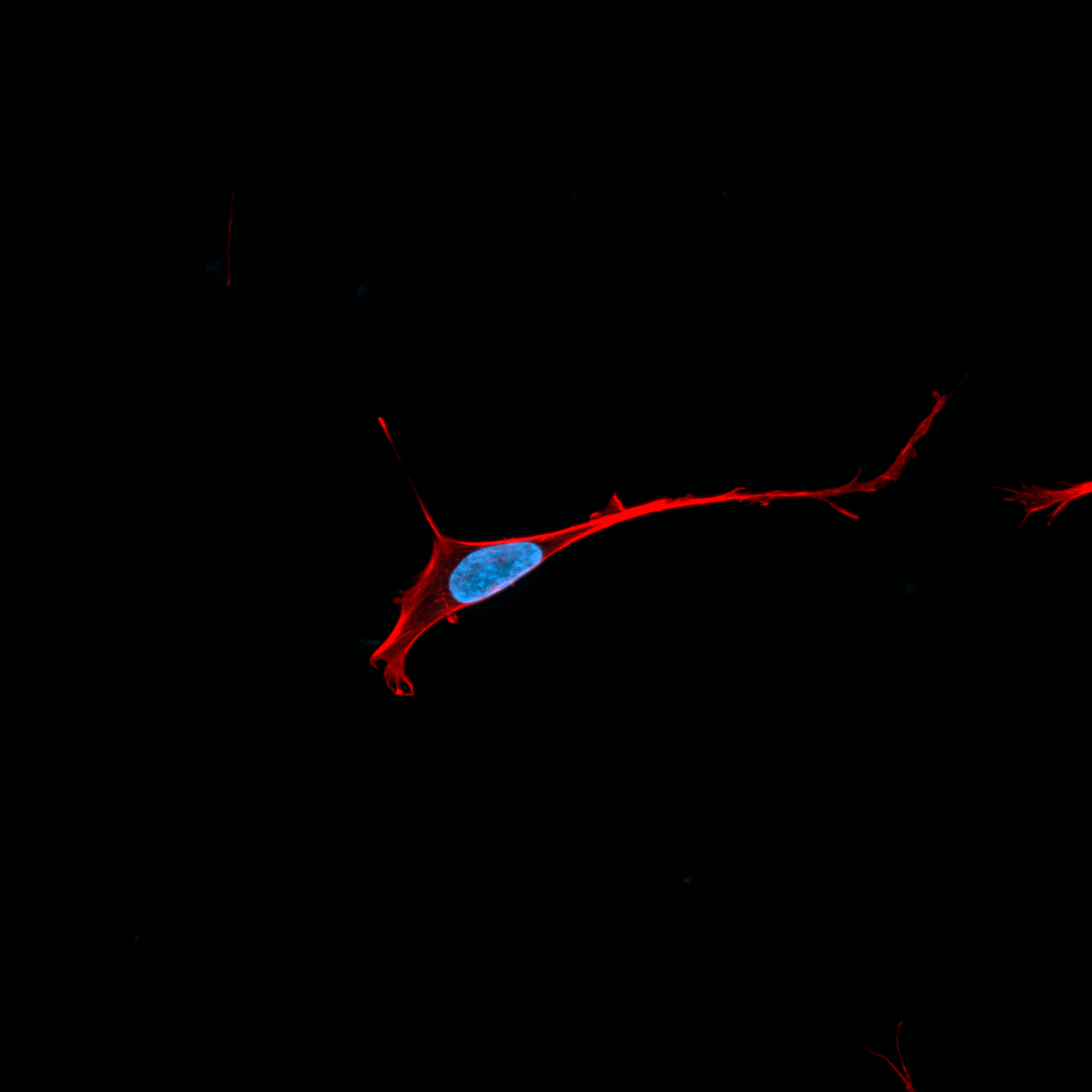

Supplement: Supplementary file 9 — Source data Fig. 6 [file 44321_2024_62_MOESM9_ESM.zip › Figure 6/Fig6E/Fig6E-INSC shRNA-5nM Taxol-Merge.tif]

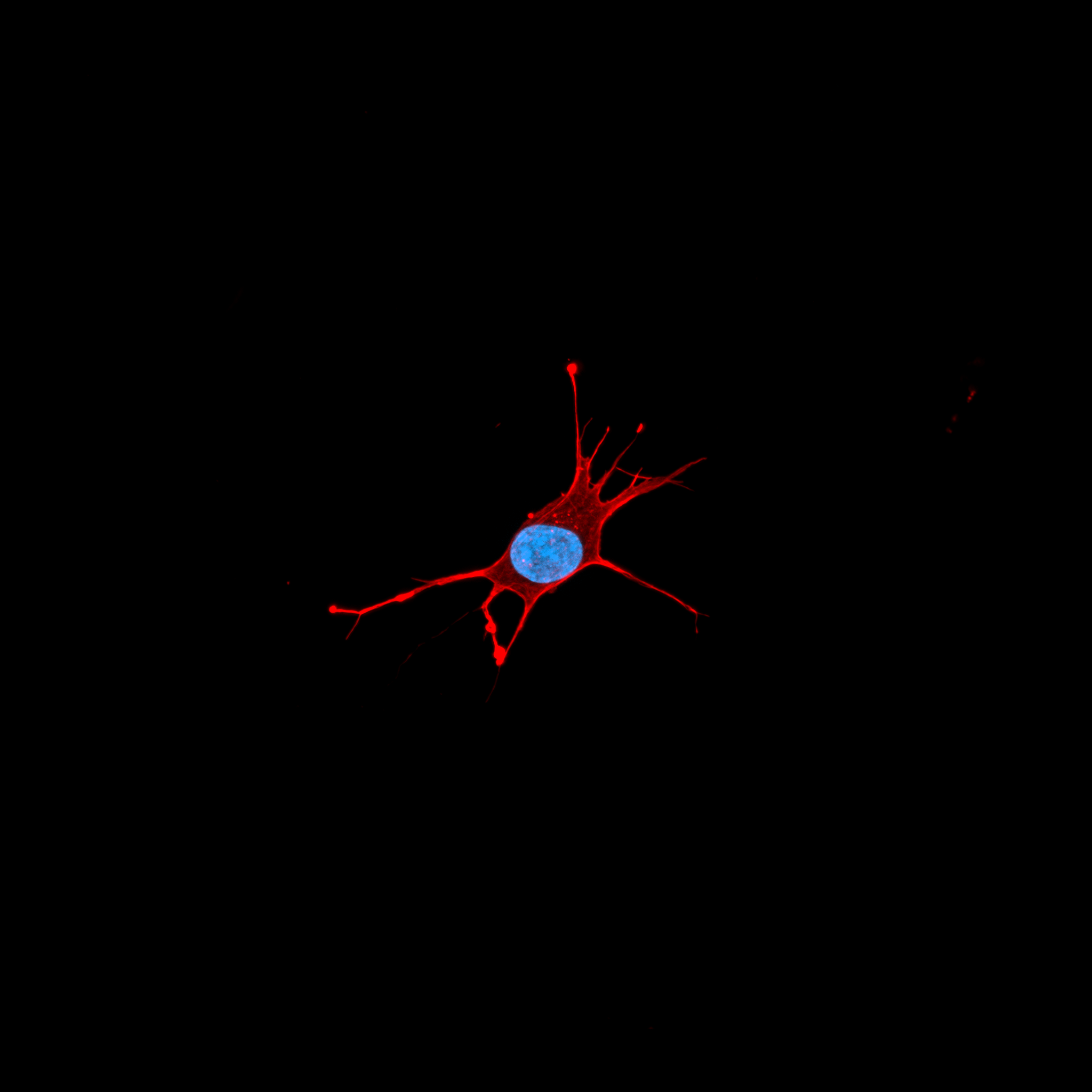

Supplement: Supplementary file 9 — Source data Fig. 6 [file 44321_2024_62_MOESM9_ESM.zip › Figure 6/Fig6E/Fig6E-INSC shRNA-DMSO-Merge.tif]

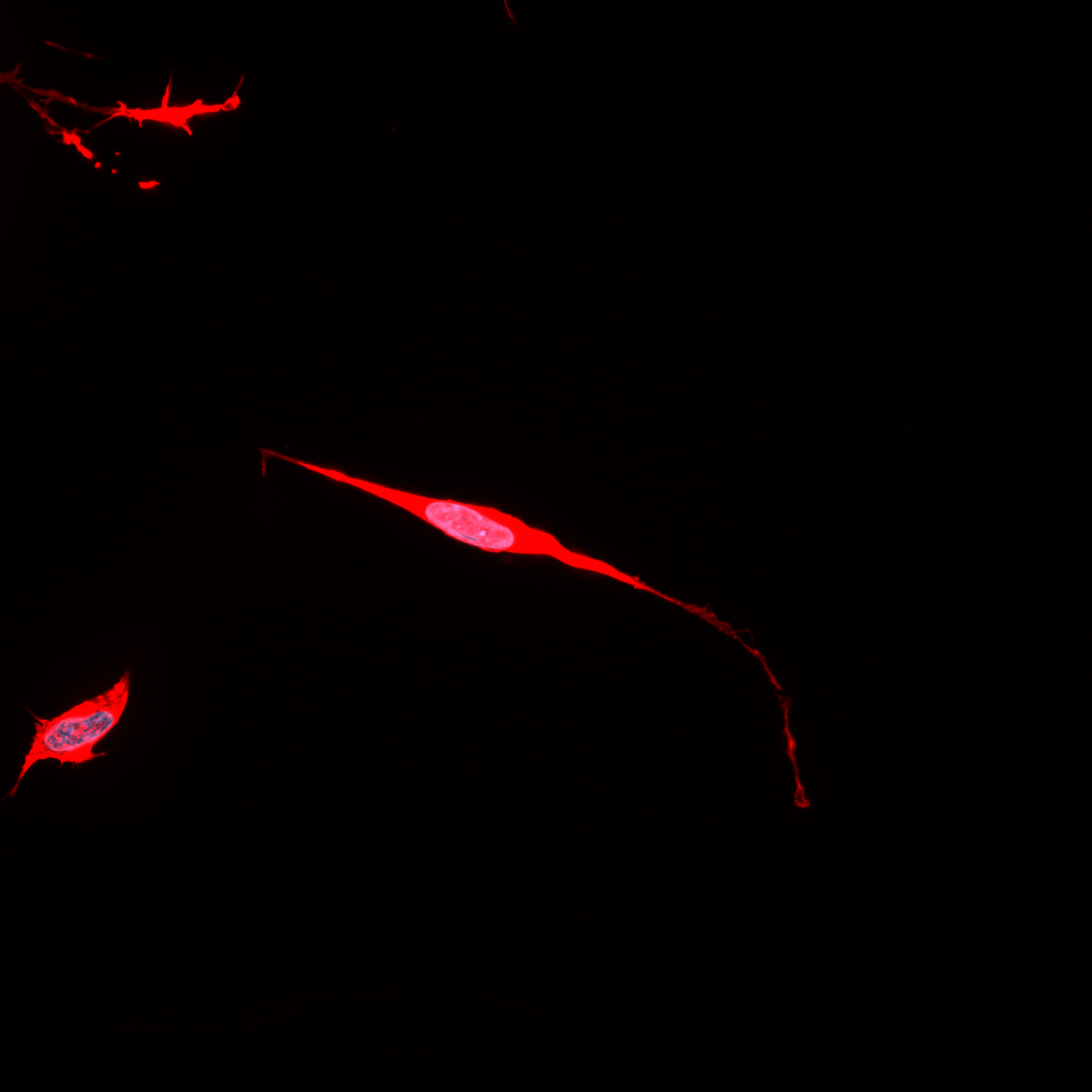

Supplement: Supplementary file 9 — Source data Fig. 6 [file 44321_2024_62_MOESM9_ESM.zip › Figure 6/Fig6E/Fig6E-scramble shRNA-DMSO-Merge.tif]

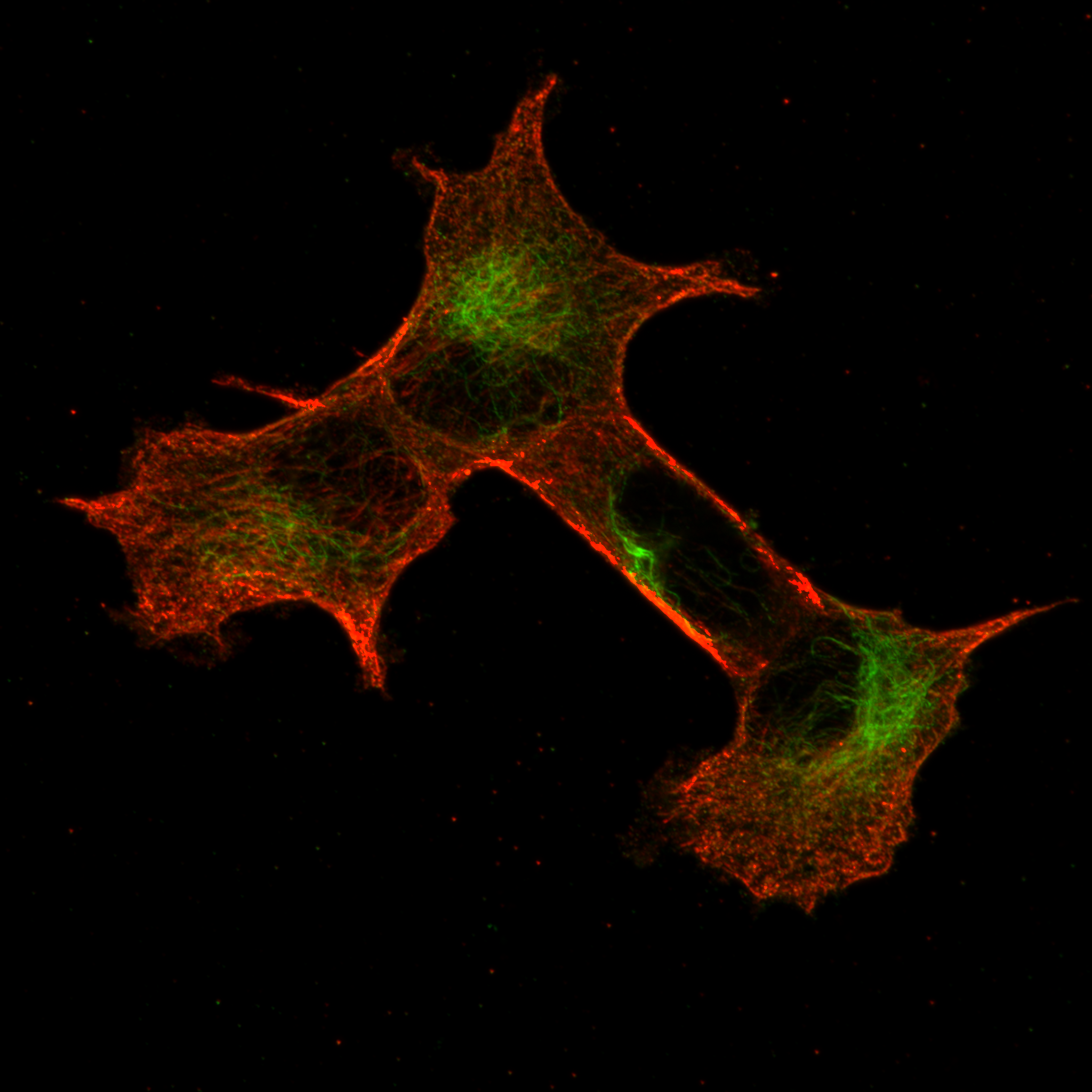

Supplement: Supplementary file 9 — Source data Fig. 6 [file 44321_2024_62_MOESM9_ESM.zip › Figure 6/Fig6G/Fig6G-INSC shRNA-DMSO-a-tubulin&acetyl-tubulin.tif]

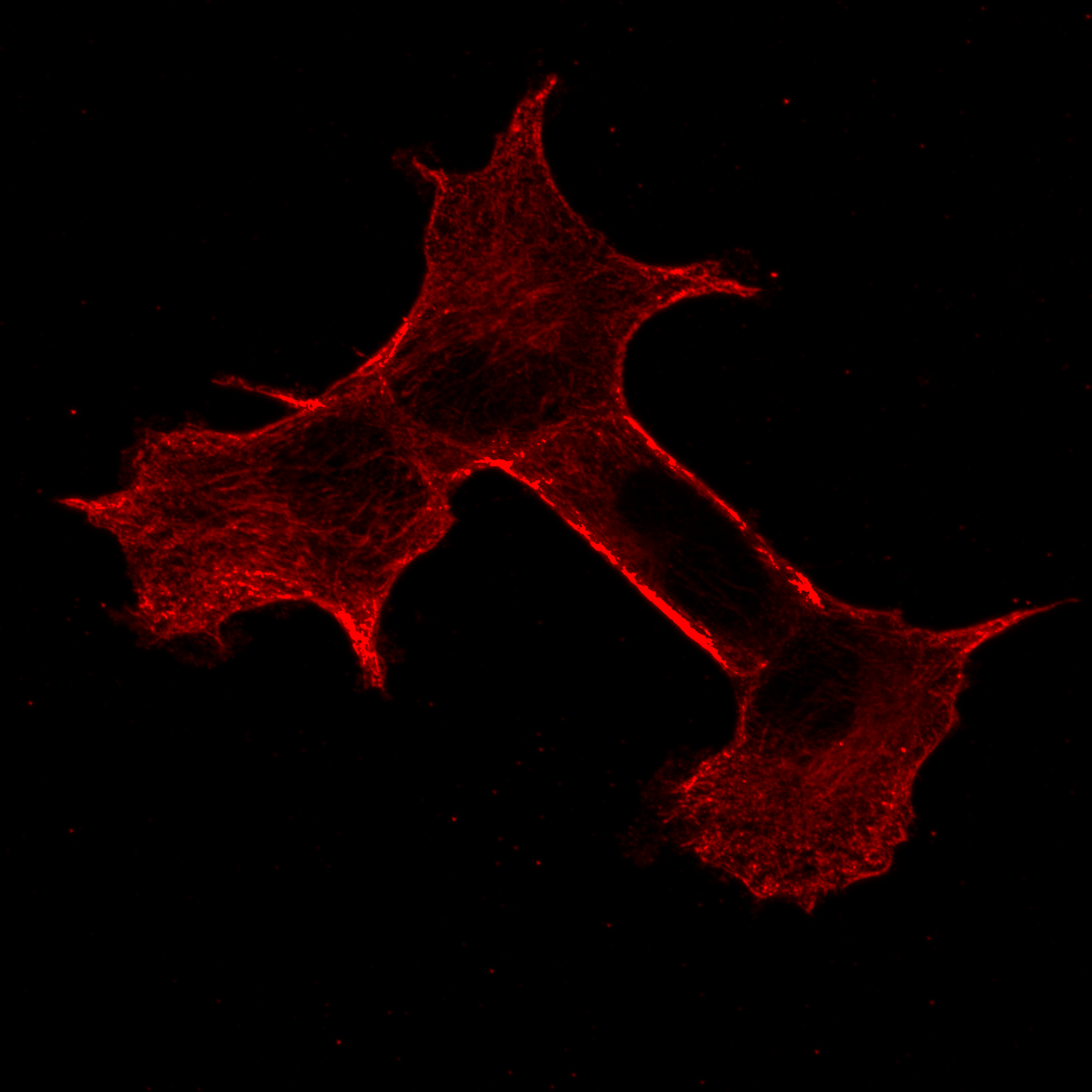

Supplement: Supplementary file 9 — Source data Fig. 6 [file 44321_2024_62_MOESM9_ESM.zip › Figure 6/Fig6G/Fig6G-INSC shRNA-DMSO-a-tubulin.tif]

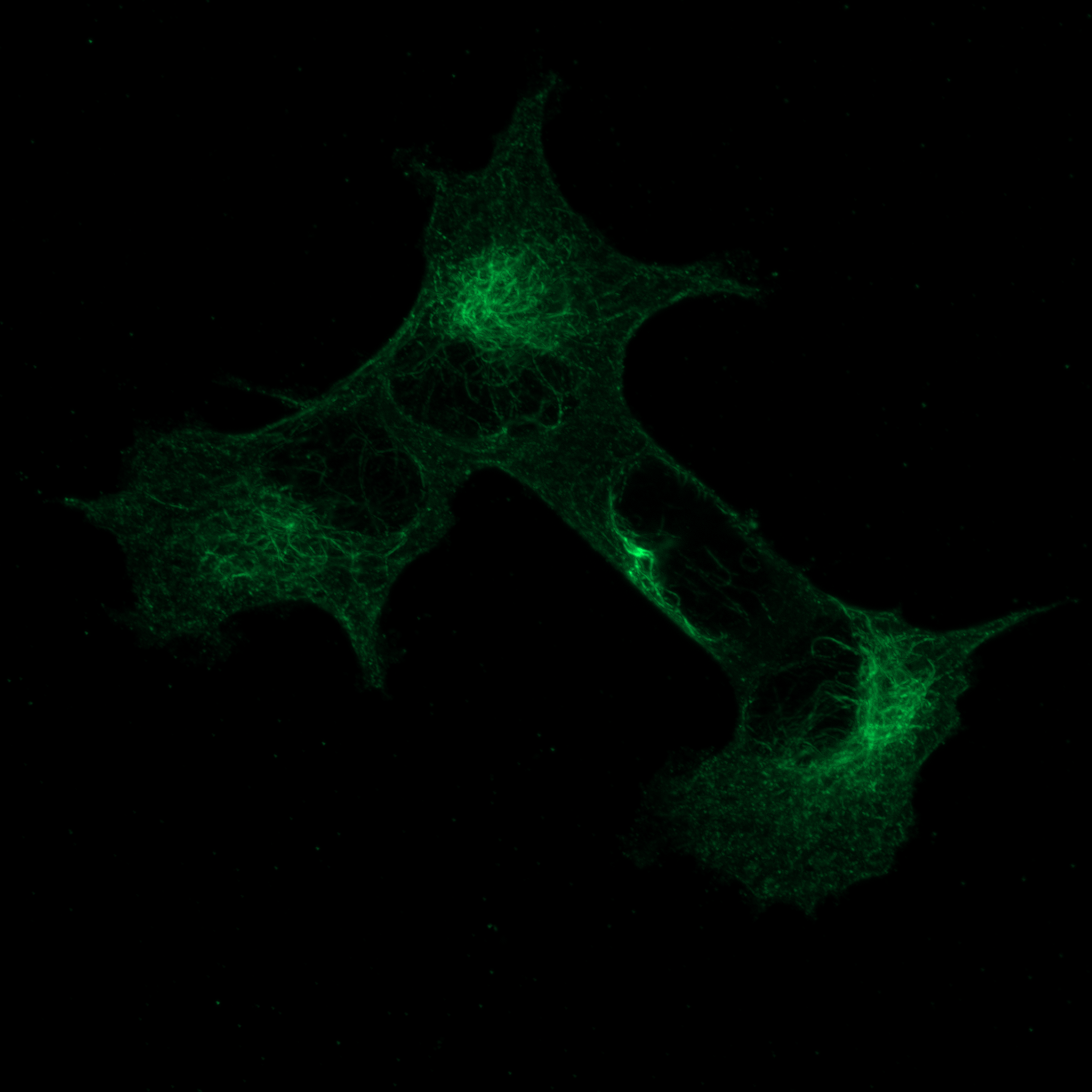

Supplement: Supplementary file 9 — Source data Fig. 6 [file 44321_2024_62_MOESM9_ESM.zip › Figure 6/Fig6G/Fig6G-INSC shRNA-DMSO-acetyl-tubulin.tif]

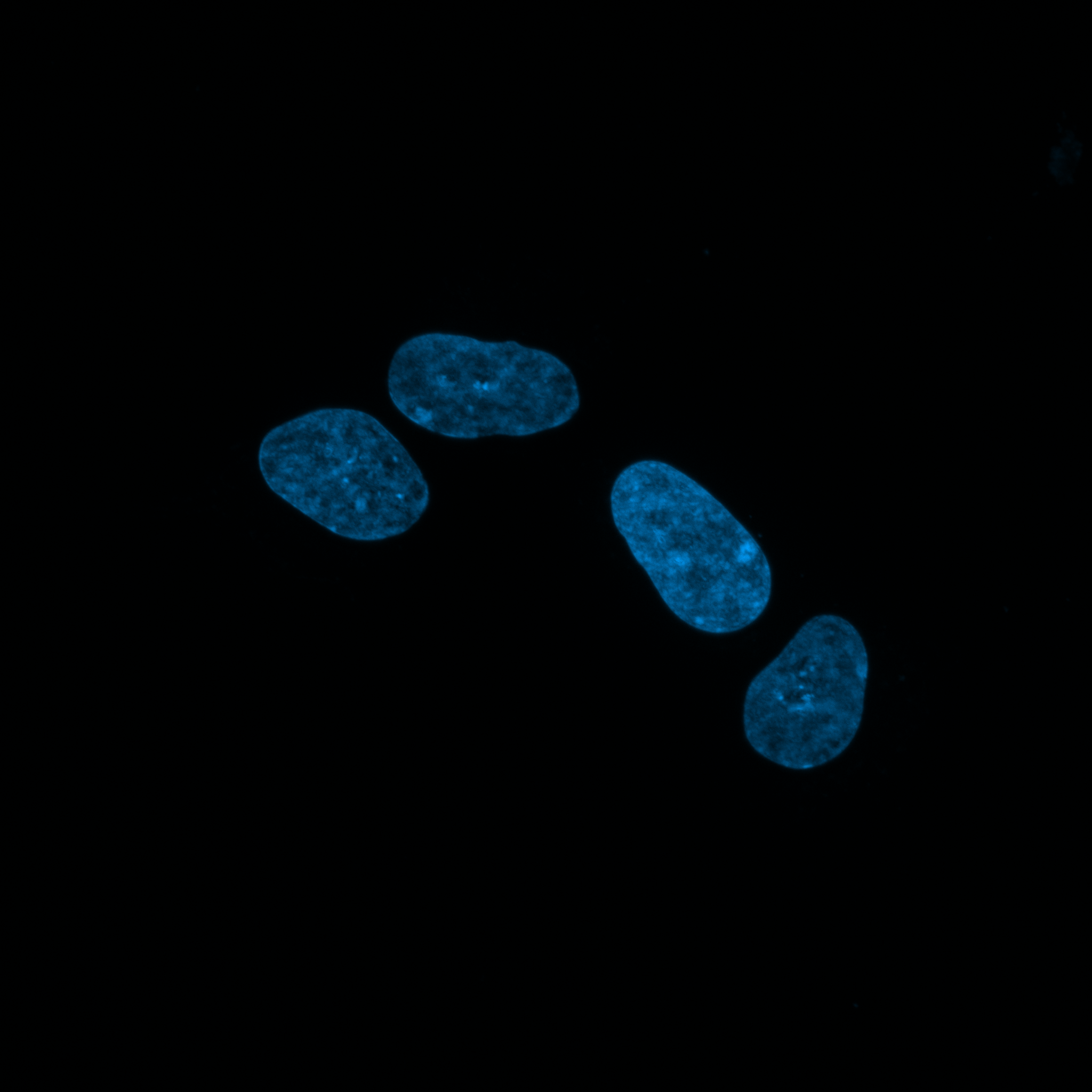

Supplement: Supplementary file 9 — Source data Fig. 6 [file 44321_2024_62_MOESM9_ESM.zip › Figure 6/Fig6G/Fig6G-INSC shRNA-DMSO-DAPI.tif]

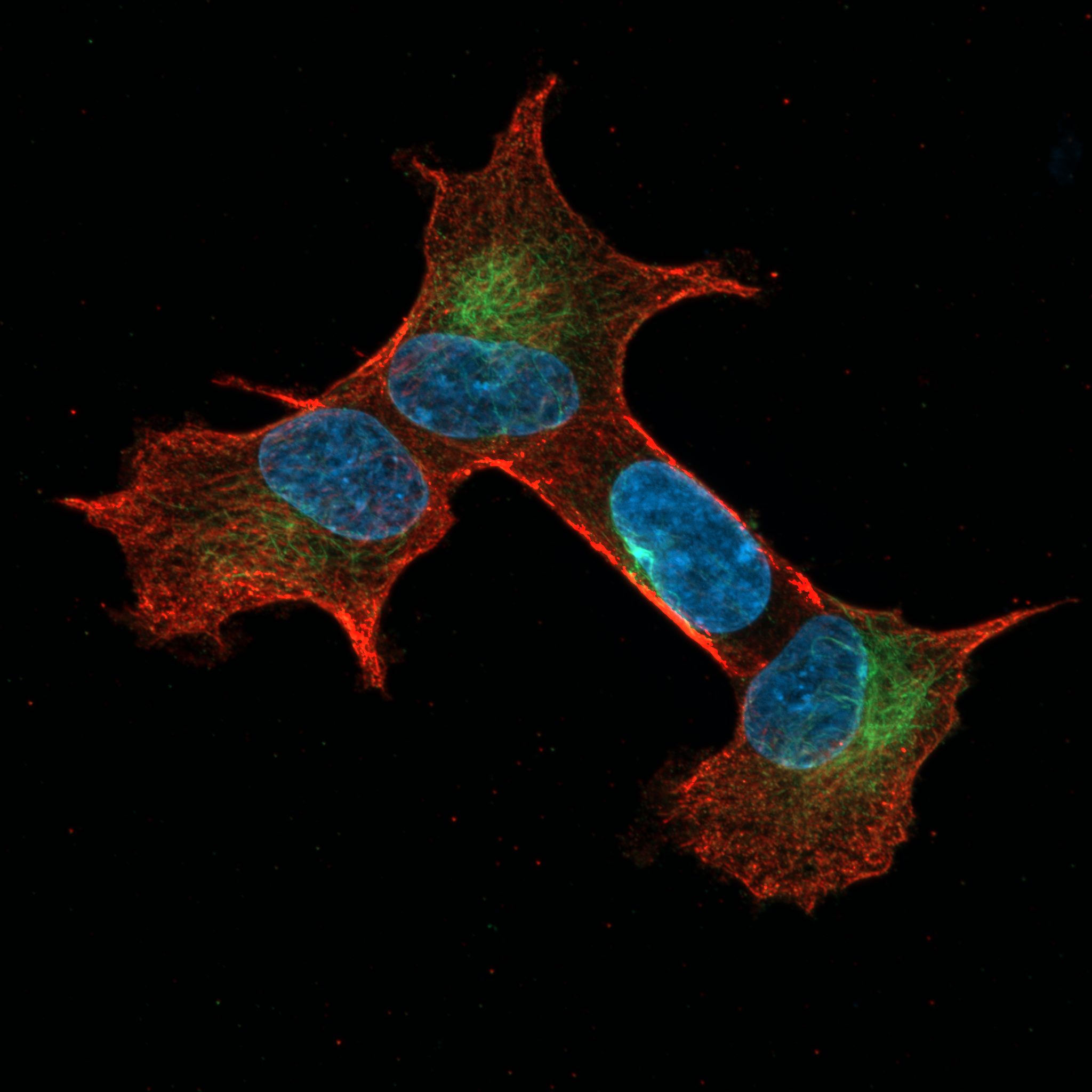

Supplement: Supplementary file 9 — Source data Fig. 6 [file 44321_2024_62_MOESM9_ESM.zip › Figure 6/Fig6G/Fig6G-INSC shRNA-DMSO-Merge.tif]

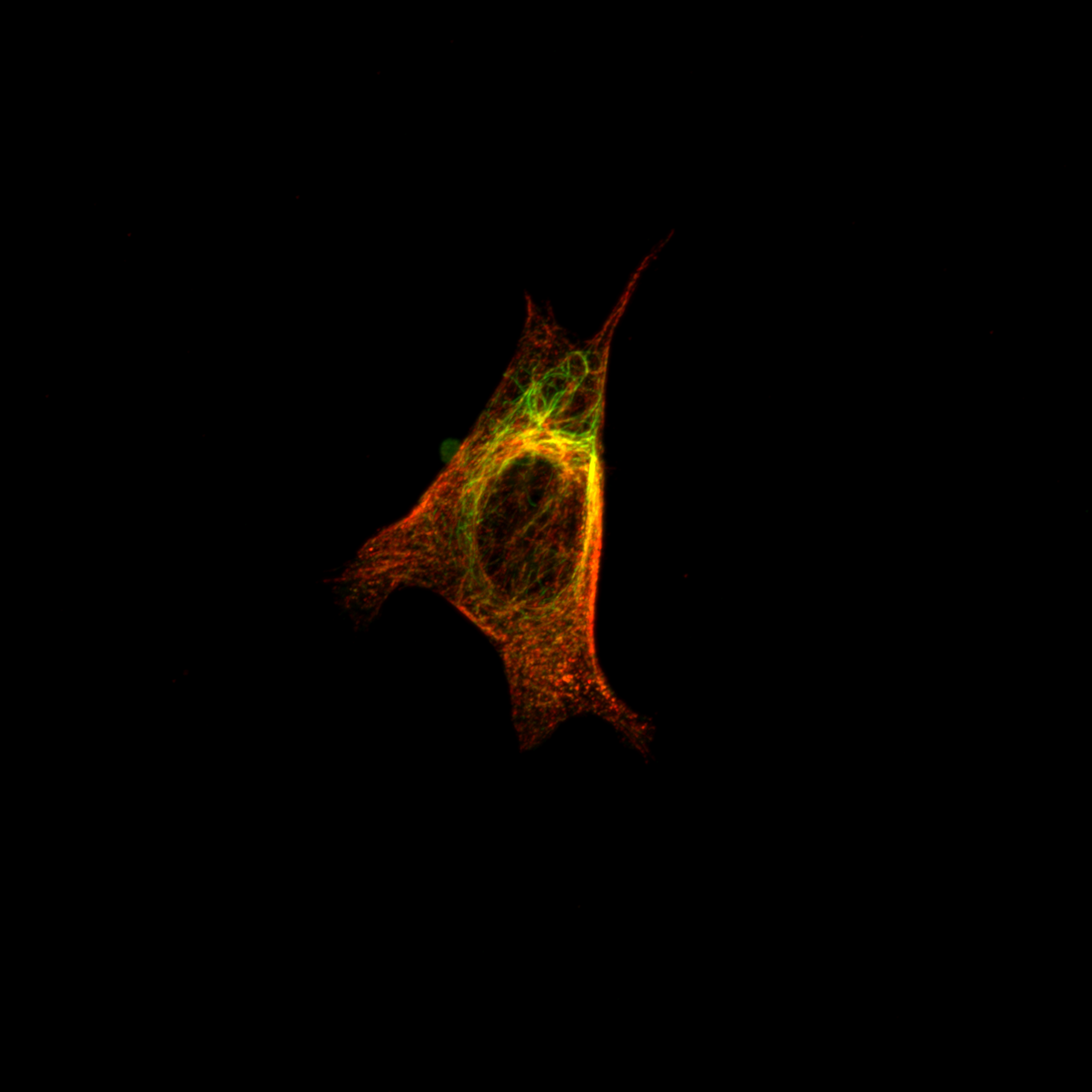

Supplement: Supplementary file 9 — Source data Fig. 6 [file 44321_2024_62_MOESM9_ESM.zip › Figure 6/Fig6G/Fig6G-scramble shRNA-DMSO-a-tubulin&acetyl-tubulin.tif]

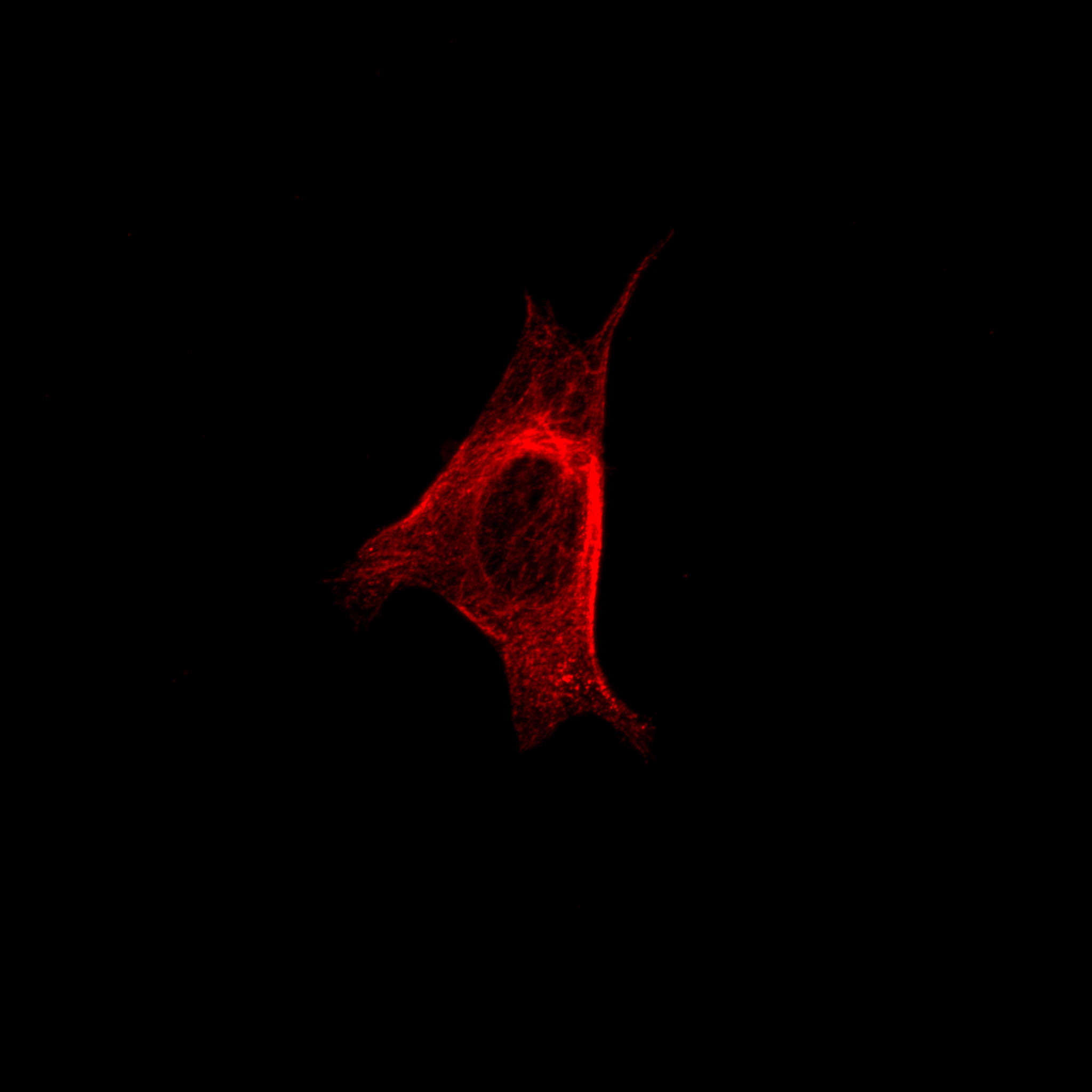

Supplement: Supplementary file 9 — Source data Fig. 6 [file 44321_2024_62_MOESM9_ESM.zip › Figure 6/Fig6G/Fig6G-scramble shRNA-DMSO-a-tubulin.tif]

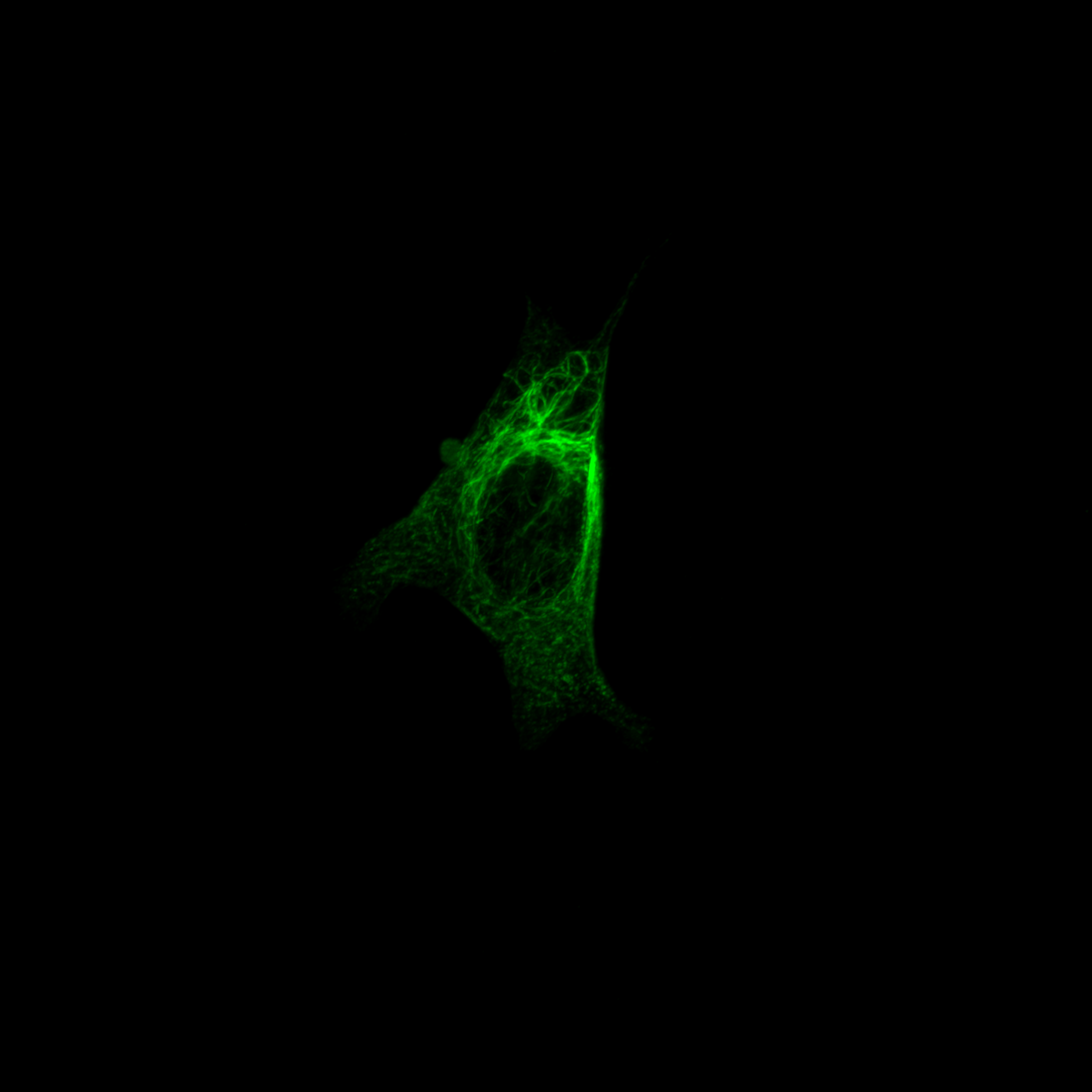

Supplement: Supplementary file 9 — Source data Fig. 6 [file 44321_2024_62_MOESM9_ESM.zip › Figure 6/Fig6G/Fig6G-scramble shRNA-DMSO-acetyl-tubulin.tif]

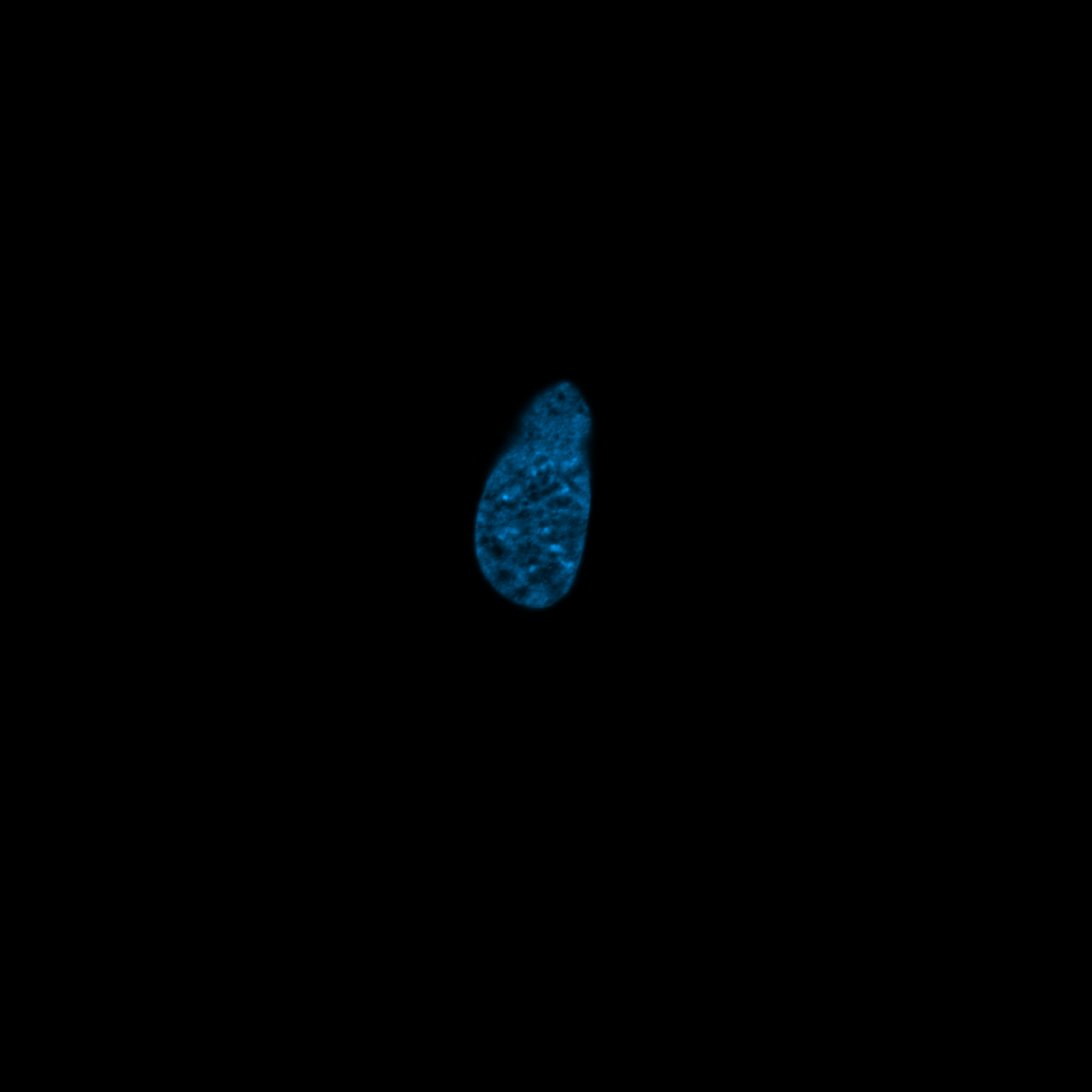

Supplement: Supplementary file 9 — Source data Fig. 6 [file 44321_2024_62_MOESM9_ESM.zip › Figure 6/Fig6G/Fig6G-scramble shRNA-DMSO-DAPI.tif]

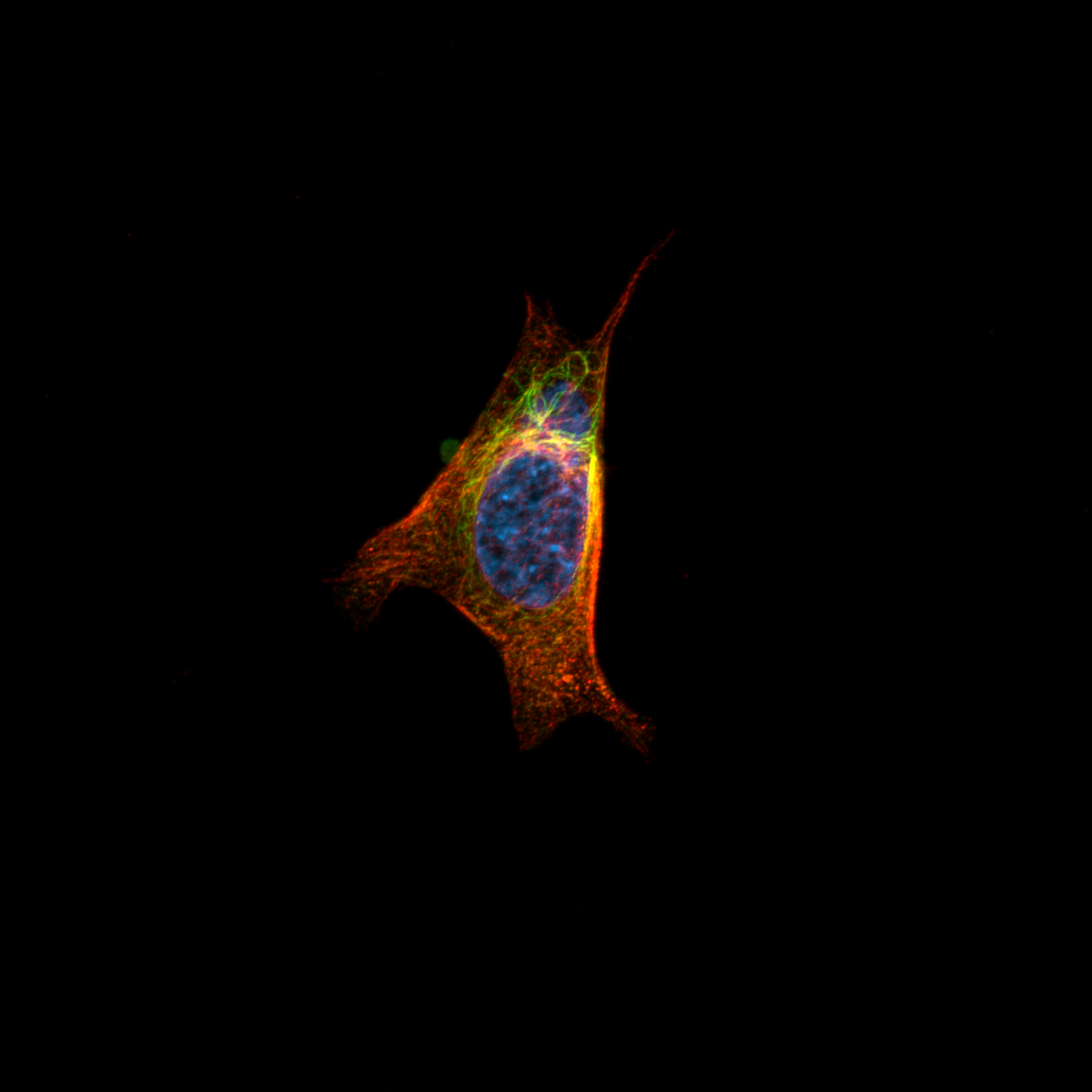

Supplement: Supplementary file 9 — Source data Fig. 6 [file 44321_2024_62_MOESM9_ESM.zip › Figure 6/Fig6G/Fig6G-scramble shRNA-DMSO-Merge.tif]

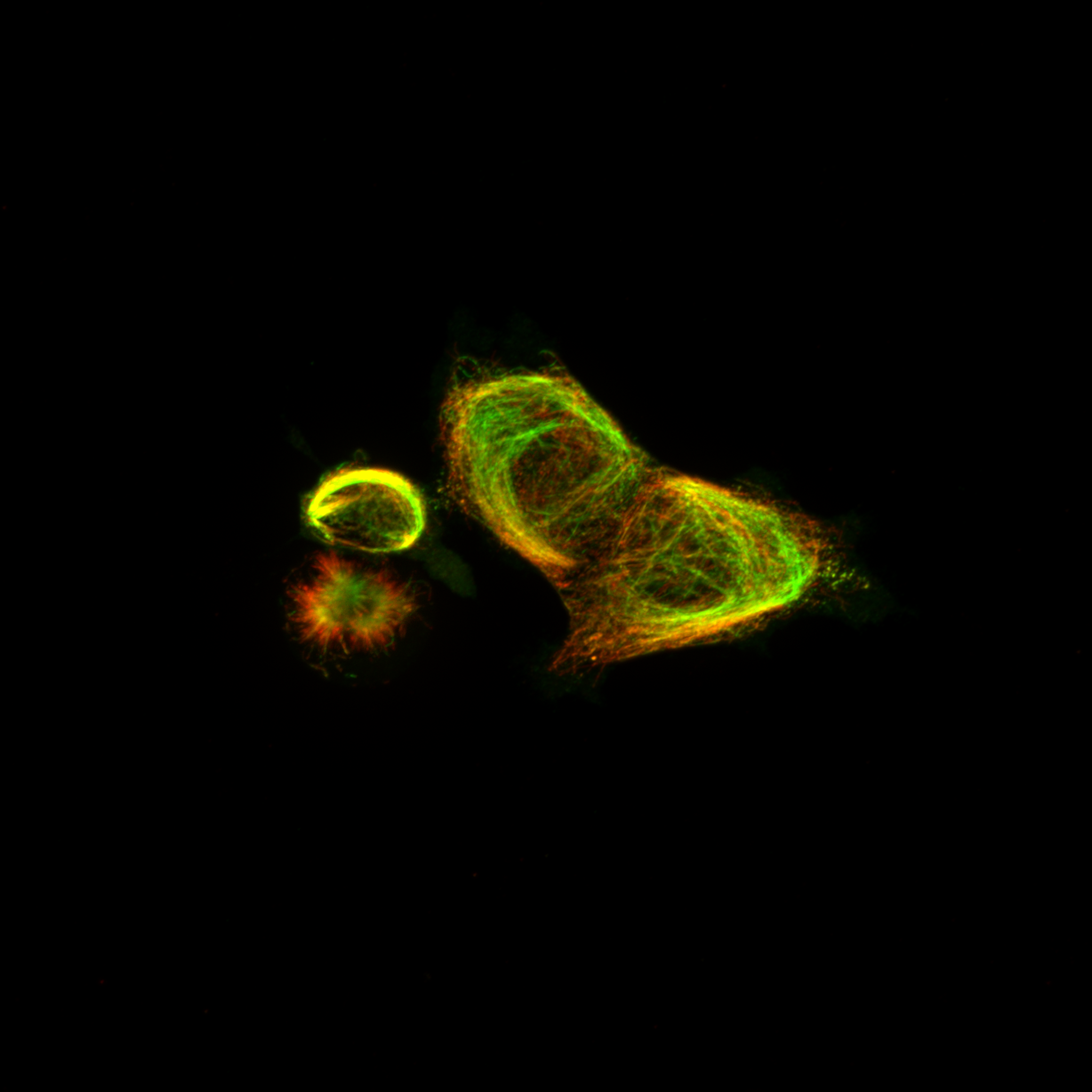

Supplement: Supplementary file 9 — Source data Fig. 6 [file 44321_2024_62_MOESM9_ESM.zip › Figure 6/Fig6I/Fig6I-INSC shRNA-Taxol-a-tubulin&acetyl-tubulin.tif]

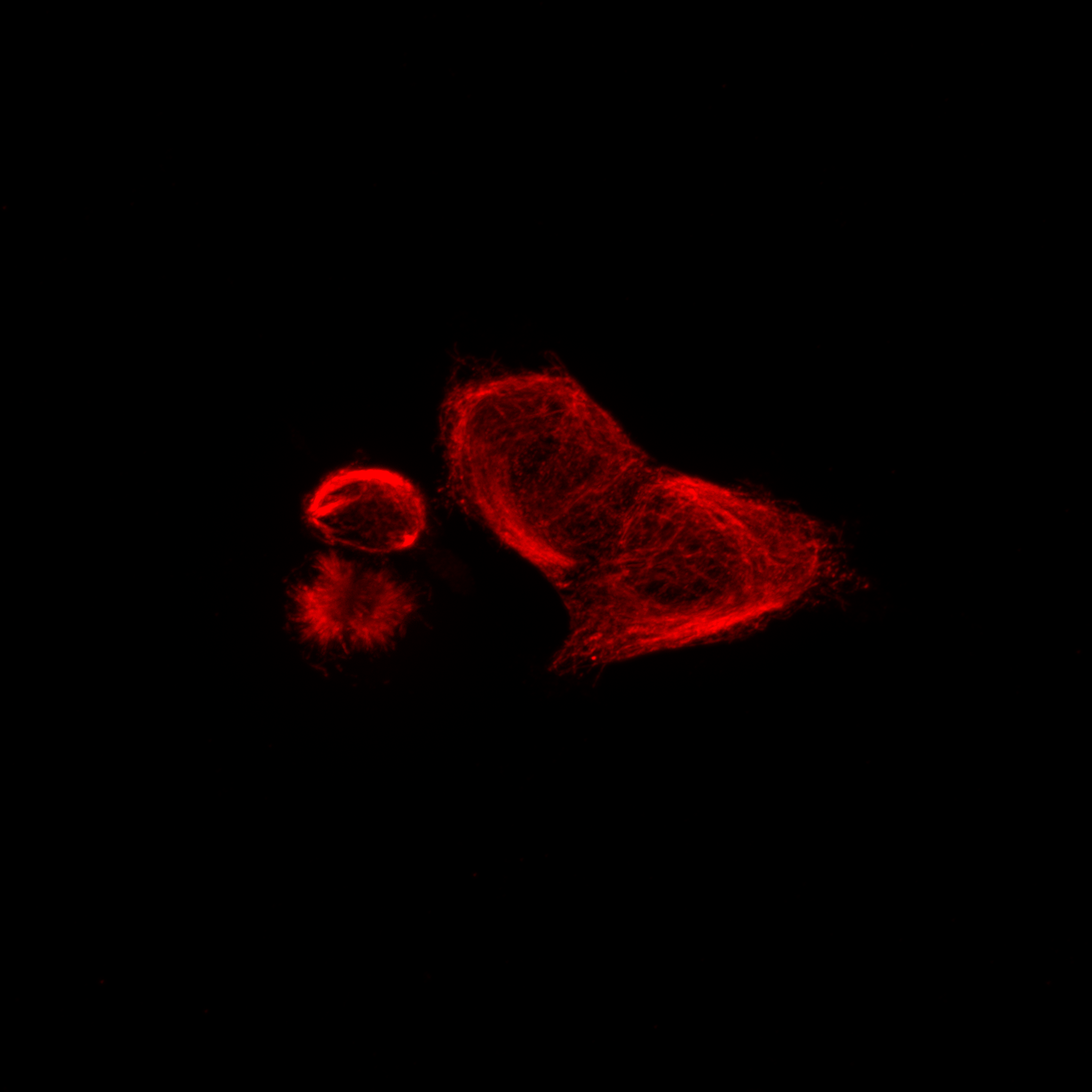

Supplement: Supplementary file 9 — Source data Fig. 6 [file 44321_2024_62_MOESM9_ESM.zip › Figure 6/Fig6I/Fig6I-INSC shRNA-Taxol-a-tubulin.tif]

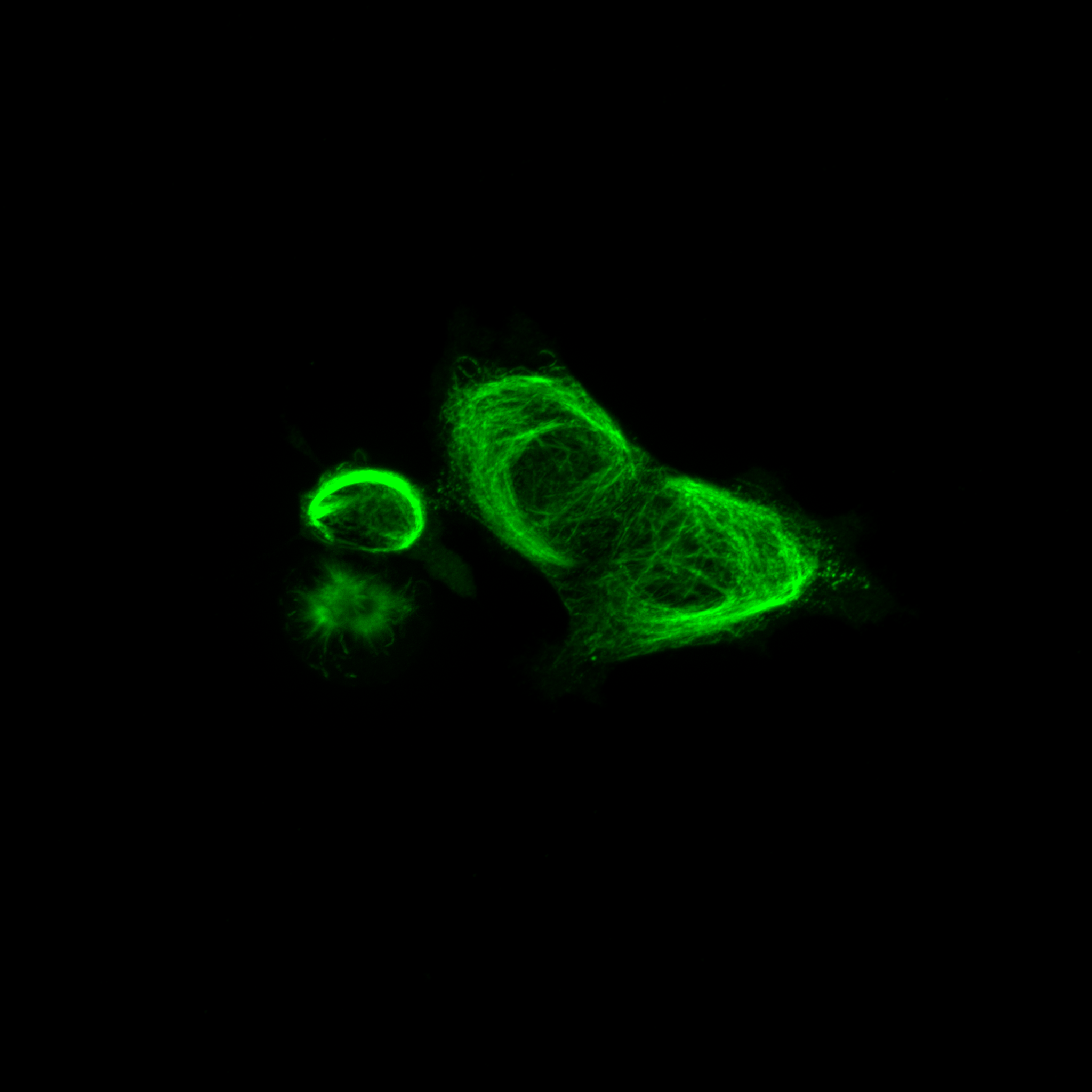

Supplement: Supplementary file 9 — Source data Fig. 6 [file 44321_2024_62_MOESM9_ESM.zip › Figure 6/Fig6I/Fig6I-INSC shRNA-Taxol-acetyl-tubulin.tif]

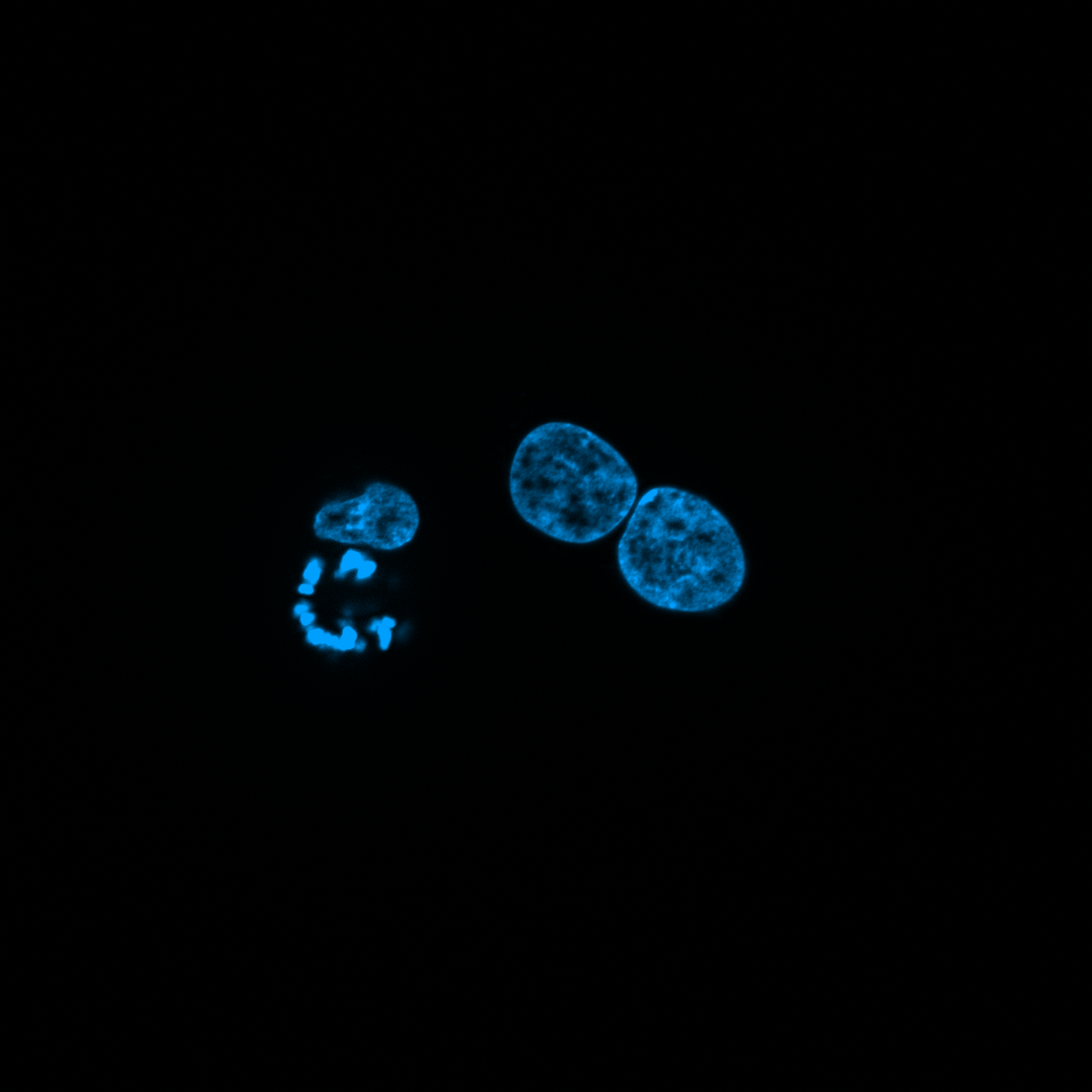

Supplement: Supplementary file 9 — Source data Fig. 6 [file 44321_2024_62_MOESM9_ESM.zip › Figure 6/Fig6I/Fig6I-INSC shRNA-Taxol-DAPI.tif]

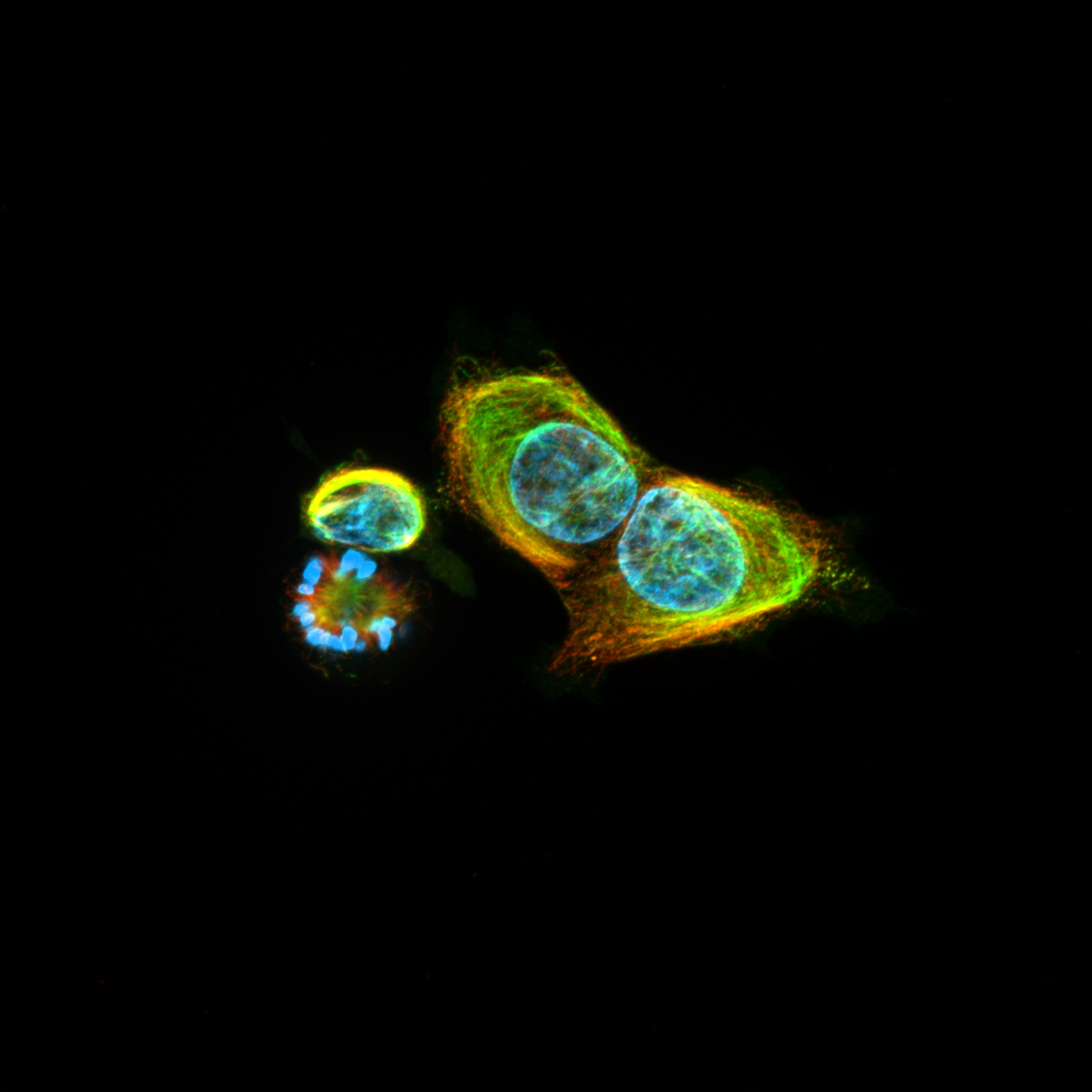

Supplement: Supplementary file 9 — Source data Fig. 6 [file 44321_2024_62_MOESM9_ESM.zip › Figure 6/Fig6I/Fig6I-INSC shRNA-Taxol-Merge.tif]

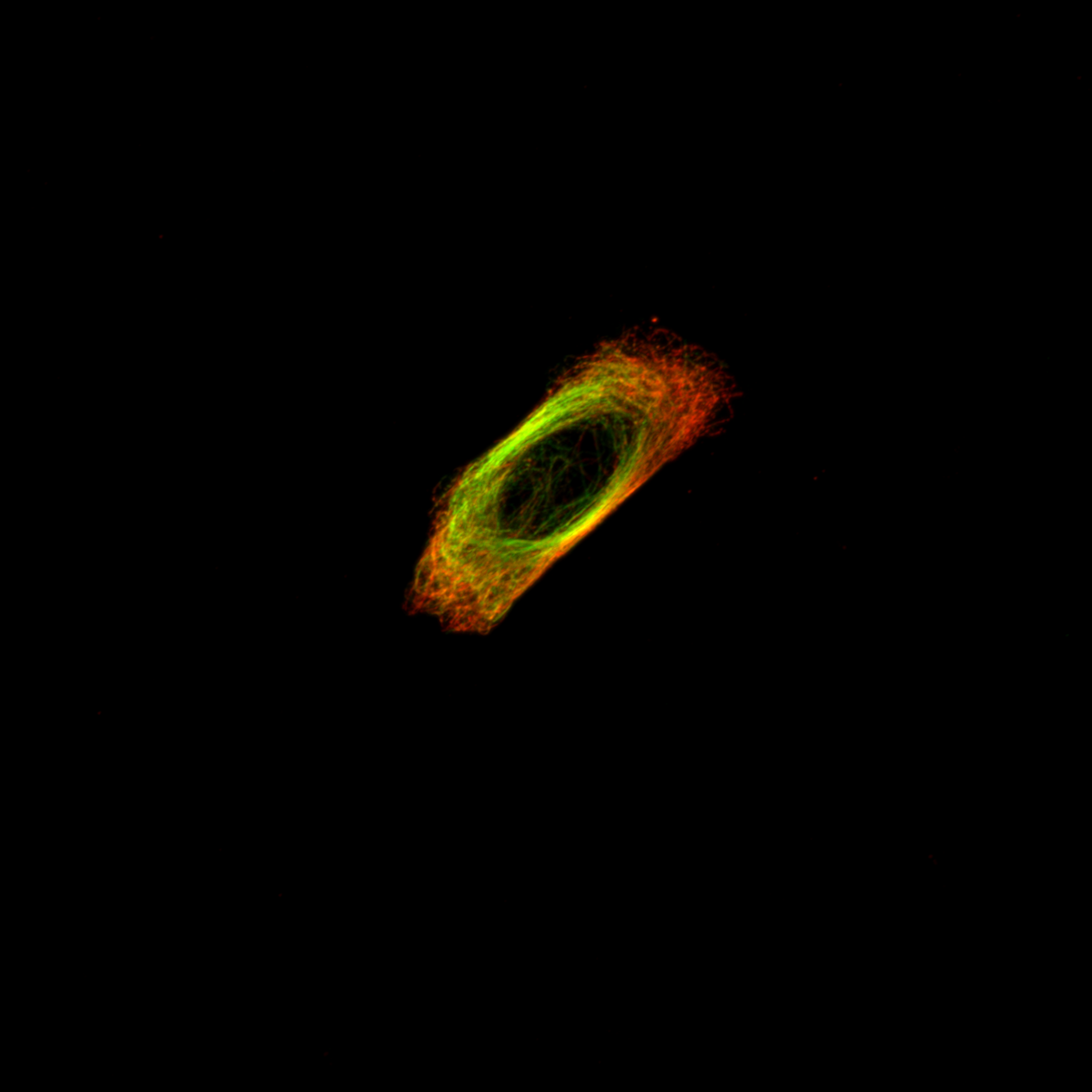

Supplement: Supplementary file 9 — Source data Fig. 6 [file 44321_2024_62_MOESM9_ESM.zip › Figure 6/Fig6I/Fig6I-scramble shRNA-Taxol-a-tubulin&acetyl-tubulin.tif]

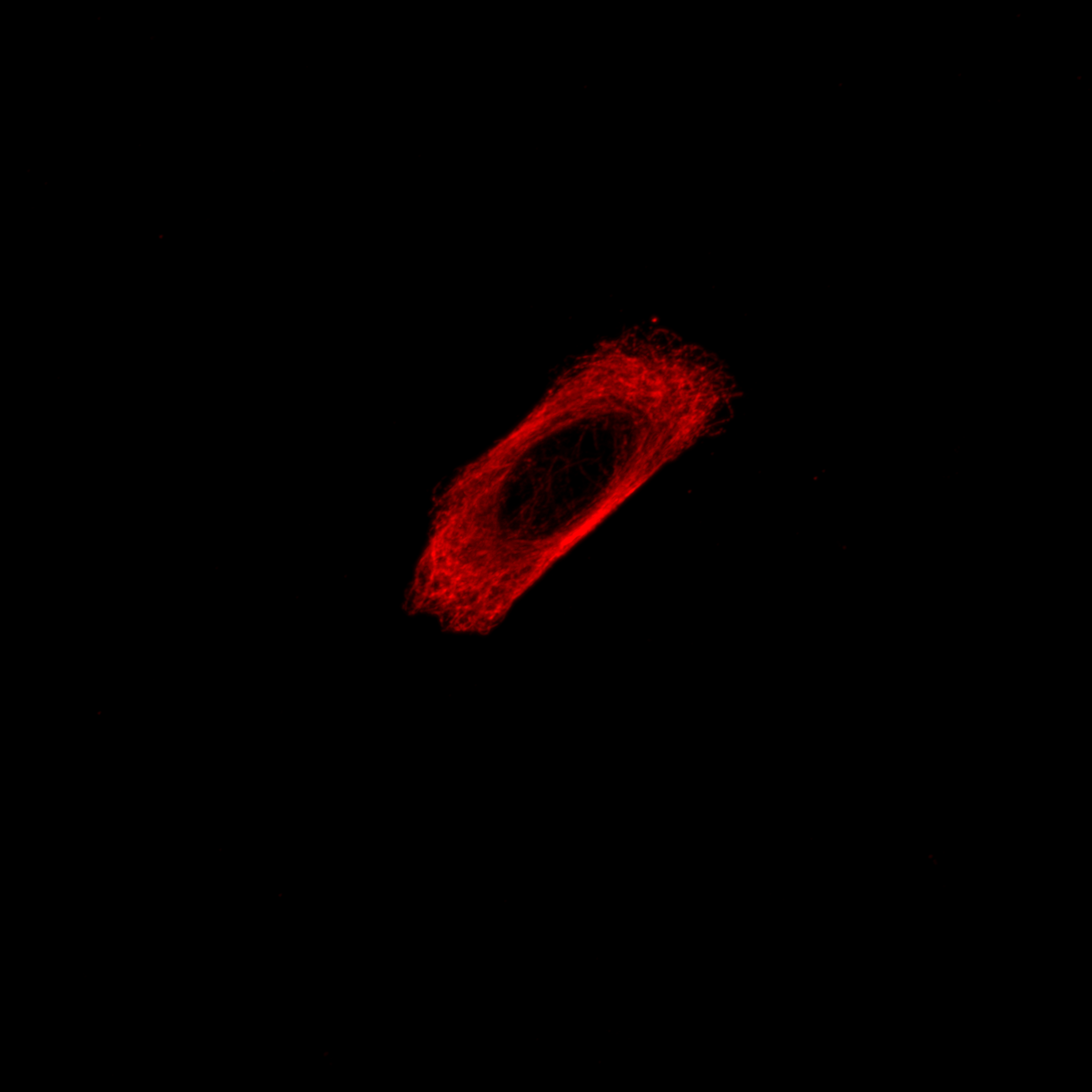

Supplement: Supplementary file 9 — Source data Fig. 6 [file 44321_2024_62_MOESM9_ESM.zip › Figure 6/Fig6I/Fig6I-scramble shRNA-Taxol-a-tubulin.tif]

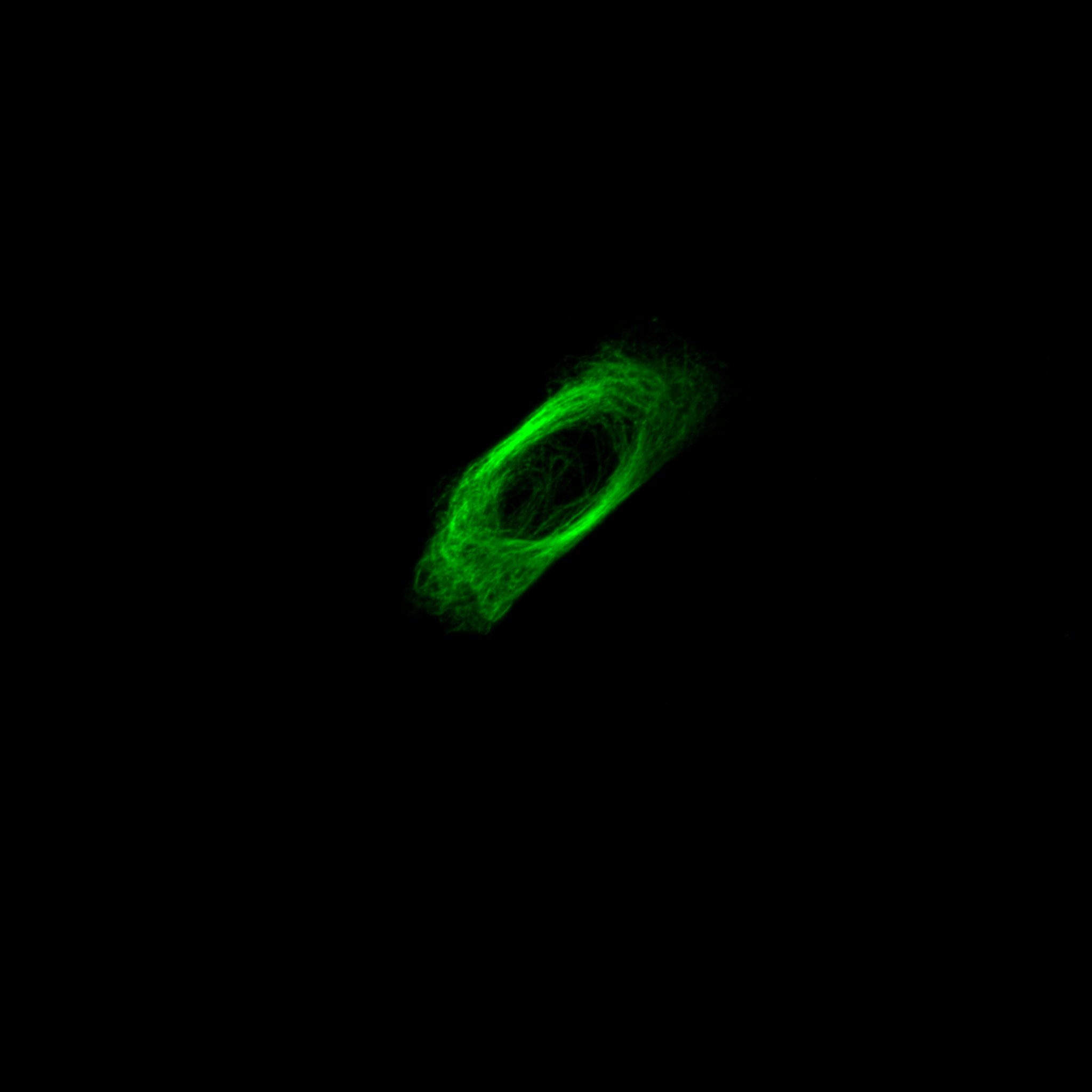

Supplement: Supplementary file 9 — Source data Fig. 6 [file 44321_2024_62_MOESM9_ESM.zip › Figure 6/Fig6I/Fig6I-scramble shRNA-Taxol-acetyl-tubulin.tif]

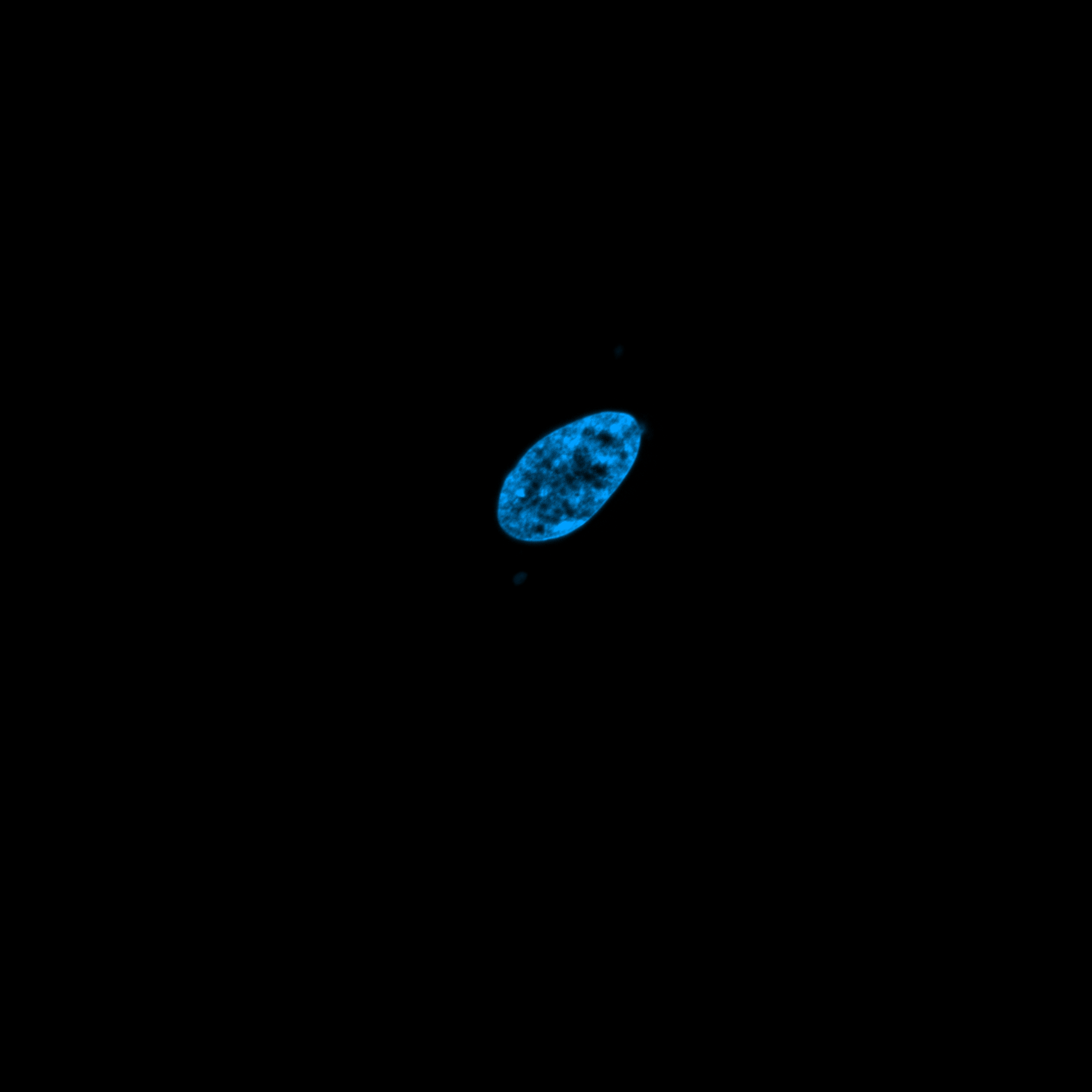

Supplement: Supplementary file 9 — Source data Fig. 6 [file 44321_2024_62_MOESM9_ESM.zip › Figure 6/Fig6I/Fig6I-scramble shRNA-Taxol-DAPI.tif]

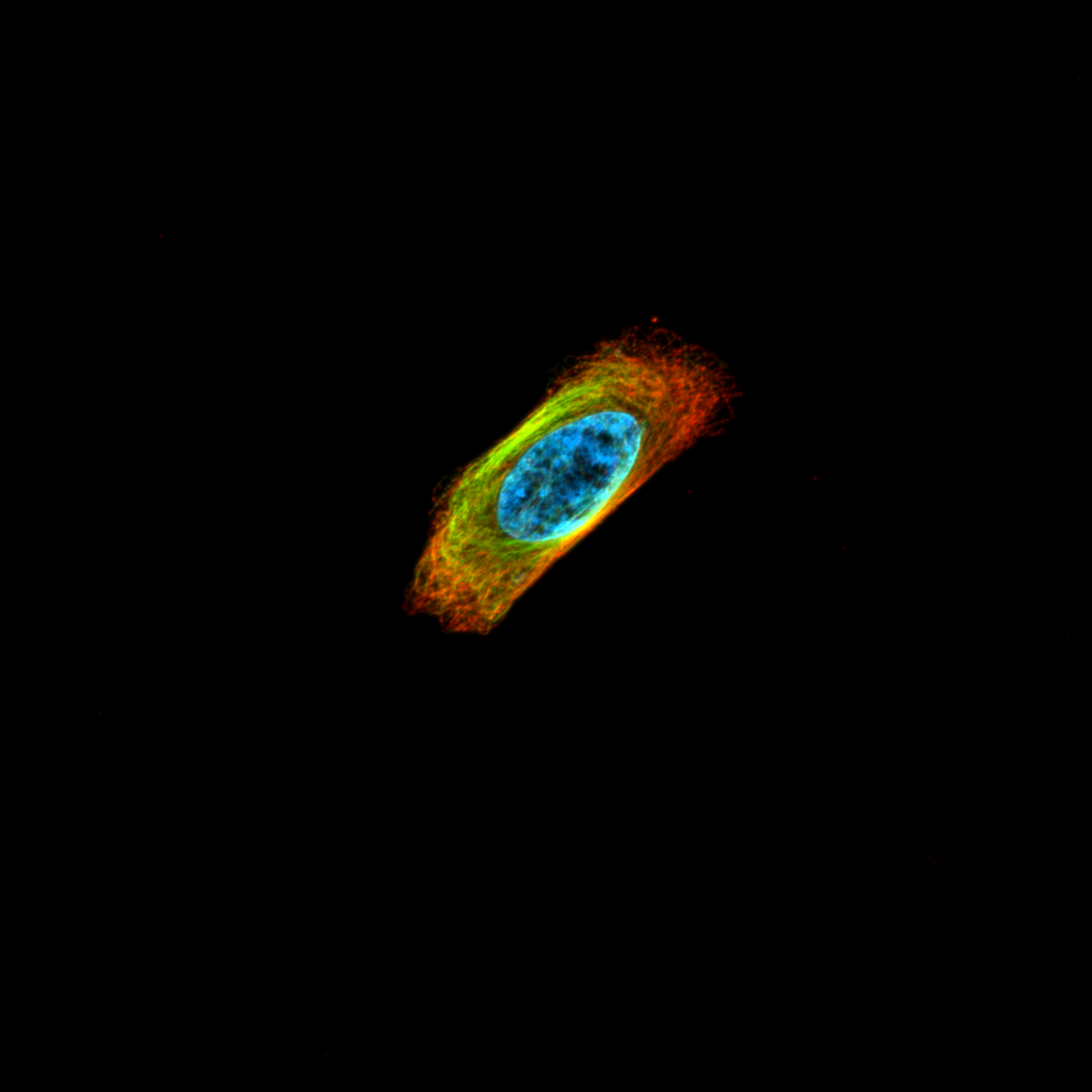

Supplement: Supplementary file 9 — Source data Fig. 6 [file 44321_2024_62_MOESM9_ESM.zip › Figure 6/Fig6I/Fig6I-scramble shRNA-Taxol-Merge.tif]
