# Supplementary material for: A missense mutation in human INSC causes peripheral neuropathy
Source: EMBO Mol Med. 2024 Apr 8;16(5):4. doi: 10.1038/s44321-024-00062-w (PMC11099080; doi:10.1038/s44321-024-00062-w)
Supplement: Supplementary file 11 — Expanded View Figures [file 44321_2024_62_MOESM11_ESM.pdf]

# Expanded View Figures

**Figure EV1. Expression of *dInsc* in FeCO neuron and its role in inducing tubulin aggregation.**

(**A**) The patterns of UAS-*mCD8-GFP* driven by *dInsc*$^{1407}$-Gal4 (left, same image in Fig. 2E) and *Iav*-Gal4 (right) in the leg. Green indicates *mCD8-GFP* and magenta indicates auto-fluorescence of the cuticle. Scale bars: 50 µm. (**B**) Representative confocal images of tubulin aggregation in the femur of 3-week-old *dInsc*$^{1407}$-Gal4>UAS-*dInsc*-RNAi flies compared with UAS-*LacZ* control. Red indicates *tubulin-mCherry*. Scale bars: 50 µm. (**C**, **D**) Three-dimensional imaging of the femur of (**D**) *dInsc*$^{1407}$-Gal4>*dInsc*-RNAi compared with (**C**) control flies. The intercellular mCherry fluorescence shows tubulin aggregation between muscle fibers. Red labeled tubulin, cyan labeled phalloidin (muscle fibers), and green indicate the auto-fluorescence of the cuticle. Arrowheads indicate the aggregative tubulin. Scale bars: 5 µm. (**E**) Representative confocal images of the adult brain of 3-week-old *dInsc*$^{1407}$-Gal4>UAS-*dInsc*-RNAi compared with the UAS-*LacZ* control flies. Green indicates mCherry fluorescence. Magenta indicates anti-DLG. Scale bars: 50 µm. (**F**) Representative confocal images of tubulin aggregation in the FeCO neuron and the femur of 3-week-old *dInsc*$^{1407}$-Gal4>*dInsc*-RNAi flies, compared with the control (UAS-*lacZ*) and the rescue (*hINSC*$^{WT}$ and *hINSC*$^{M70R}$) groups. Red indicates tubulin tracker signals. Green indicates the auto-fluorescence of cuticles. The FeCO neurons are encircled by the dashed line. Scale bars: 5 µm.

 © The Author(s)

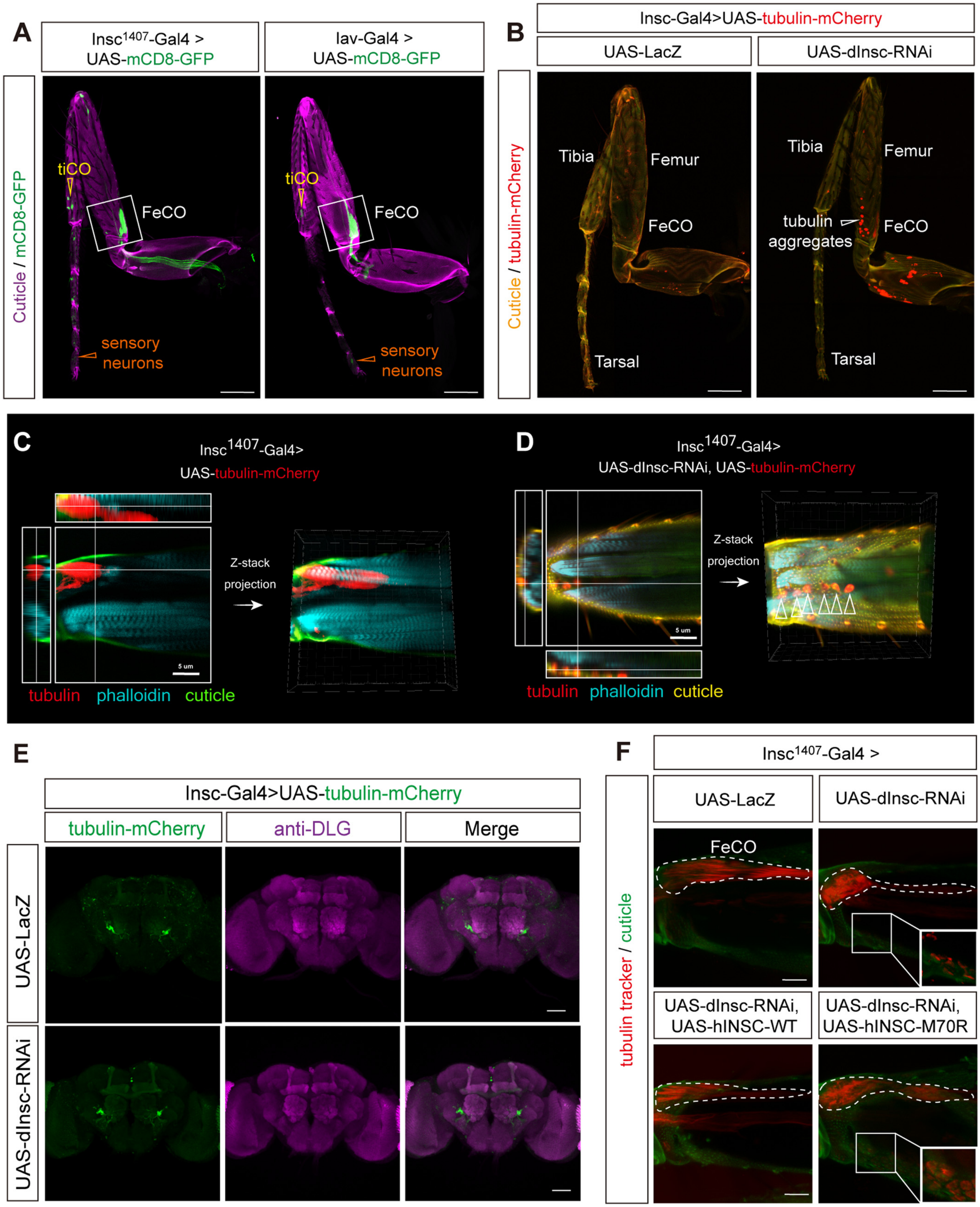
A
Insc1407-Gal4 > UAS-mCD8-GFP
Iav-Gal4 > UAS-mCD8-GFP
Cuticle / mCD8-GFP
tiCO
FeCO
sensory neurons
B
Insc-Gal4>UAS-tubulin-mCherry
UAS-LacZ
UAS-dInsc-RNAi
Cuticle / tubulin-mCherry
Tibia
Femur
FeCO
Tarsal
tubulin aggregates
C
Insc1407-Gal4> UAS-tubulin-mCherry
Z-stack projection
5 um
tubulin phalloidin cuticle
D
Insc1407-Gal4> UAS-dInsc-RNAi, UAS-tubulin-mCherry
Z-stack projection
5 um
tubulin phalloidin cuticle
E
Insc-Gal4>UAS-tubulin-mCherry
tubulin-mCherry
anti-DLG
Merge
UAS-LacZ
UAS-dInsc-RNAi
F
Insc1407-Gal4 >
UAS-LacZ
UAS-dInsc-RNAi
FeCO
tubulin tracker / cuticle
UAS-dInsc-RNAi, UAS-hINSC-WT
UAS-dInsc-RNAi, UAS-hINSC-M70R

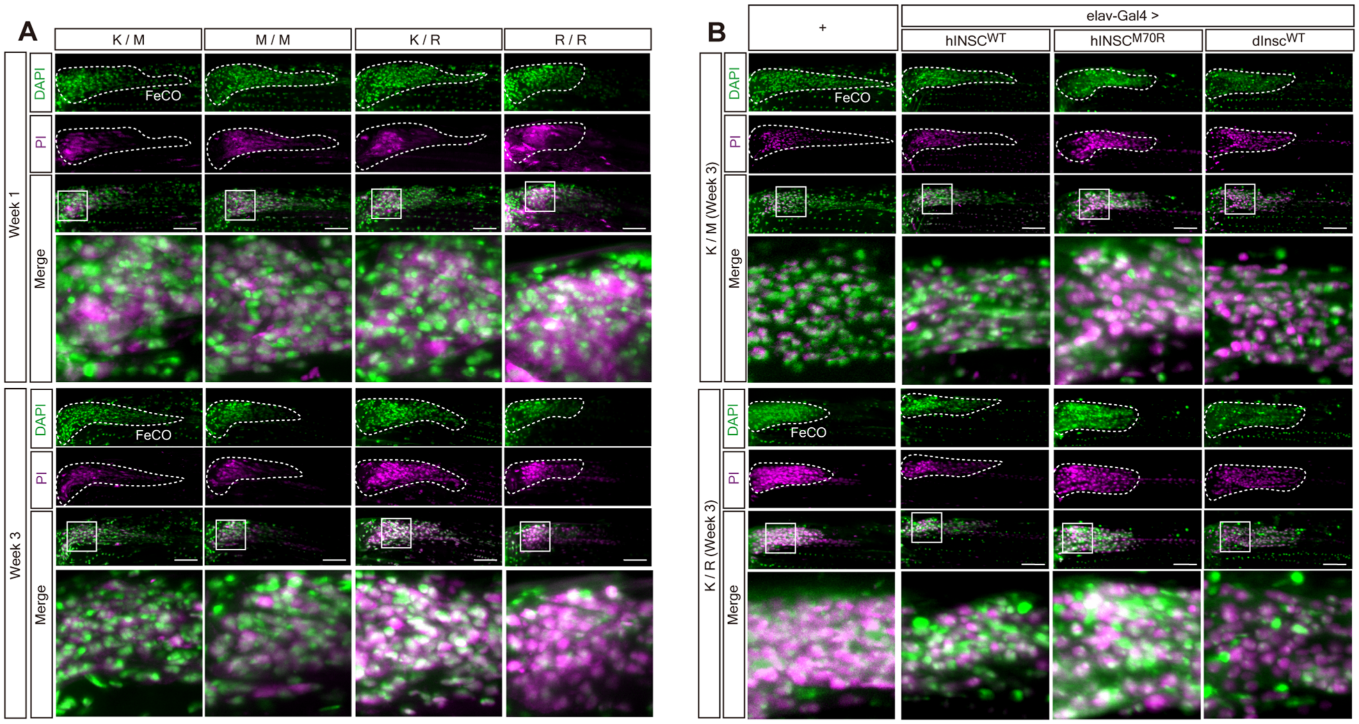


**Figure EV2. Representative confocal images of DAPI and PI co-staining in K305M and K305R CRISPR-KI flies.**

(**A**) Representative confocal images of FeCO neurons of 1- and 3-week-old *dInsc*$^{+/K305R}$ (*K/R*) and *dInsc*$^{K305R/K305R}$ (*R/R*) flies, co-stained with PI (magenta) and DAPI (green), compared with *dInsc*$^{+/K305M}$ (*K/M*) and *dInsc*$^{K305M/K305M}$ (*M/M*) CRISPR-KI flies. The FeCO neurons are encircled by the dashed line. Scale bar: 5 μm. (**B**) Representative confocal images of FeCO neurons of *K/M* and *K/R* flies overexpressing *hINSC*$^{WT}$, *hINSC*$^{M70R}$ and *dInsc*$^{WT}$ under the control of pan-neuronal *elav*-Gal4, co-stained with PI (magenta) and DAPI (green), compared with *K/M* and *K/R* CRISPR-KI flies, respectively. All flies are aged to 3 weeks. The FeCO neurons are encircled by the dashed line. Scale bar: 5 μm.

 © The Author(s)

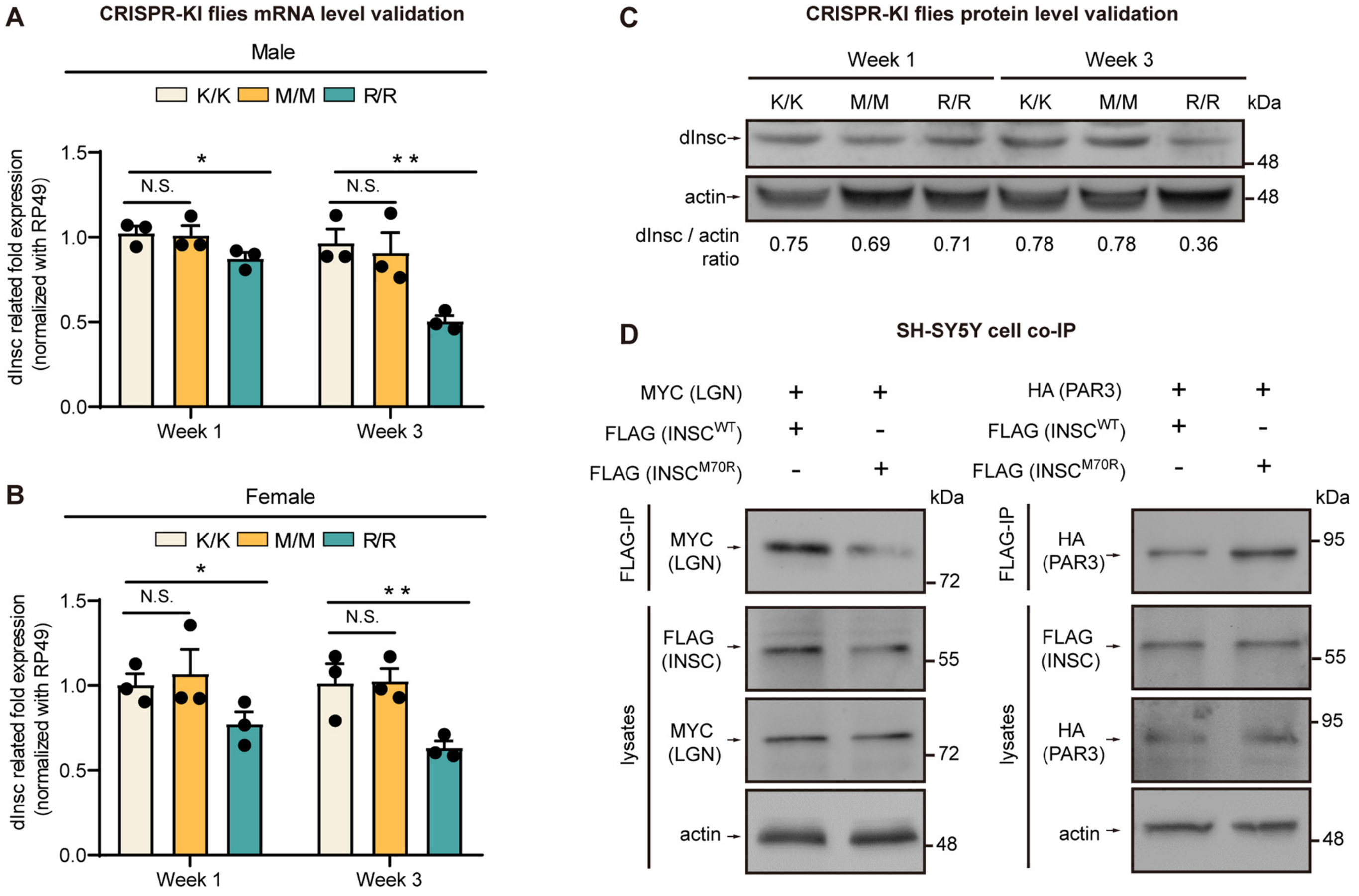


**Figure EV3. Both mRNA and protein level of R/R decrease in aging flies induced by PIL complex dysregulation.**

(**A**) Relative mRNA abundance in whole fly extracts of *M/M* and *R/R* male flies with its corresponding *K/K* control in week 1 and week 3; $n = 12$ flies/genotype from 3 independent technical replicates. (**B**) Relative mRNA abundance in whole fly extracts of *M/M* and *R/R* female flies with its corresponding *K/K* control in week 1 and week 3; $n = 12$ flies/genotype from three independent technical replicates. (**C**) Representative western blotting of whole fly extracts from *M/M* and *R/R* flies with its corresponding *K/K* control in week 1 and week 3; $n = 5$ flies/genotype. (**D**) Co-immunoprecipitation to examine the association of FLAG-hINSC (WT and M70R) with MYC-LGN (left) or HA-PAR3 (right) in SH-SY5Y cells.

© The Author(s) 

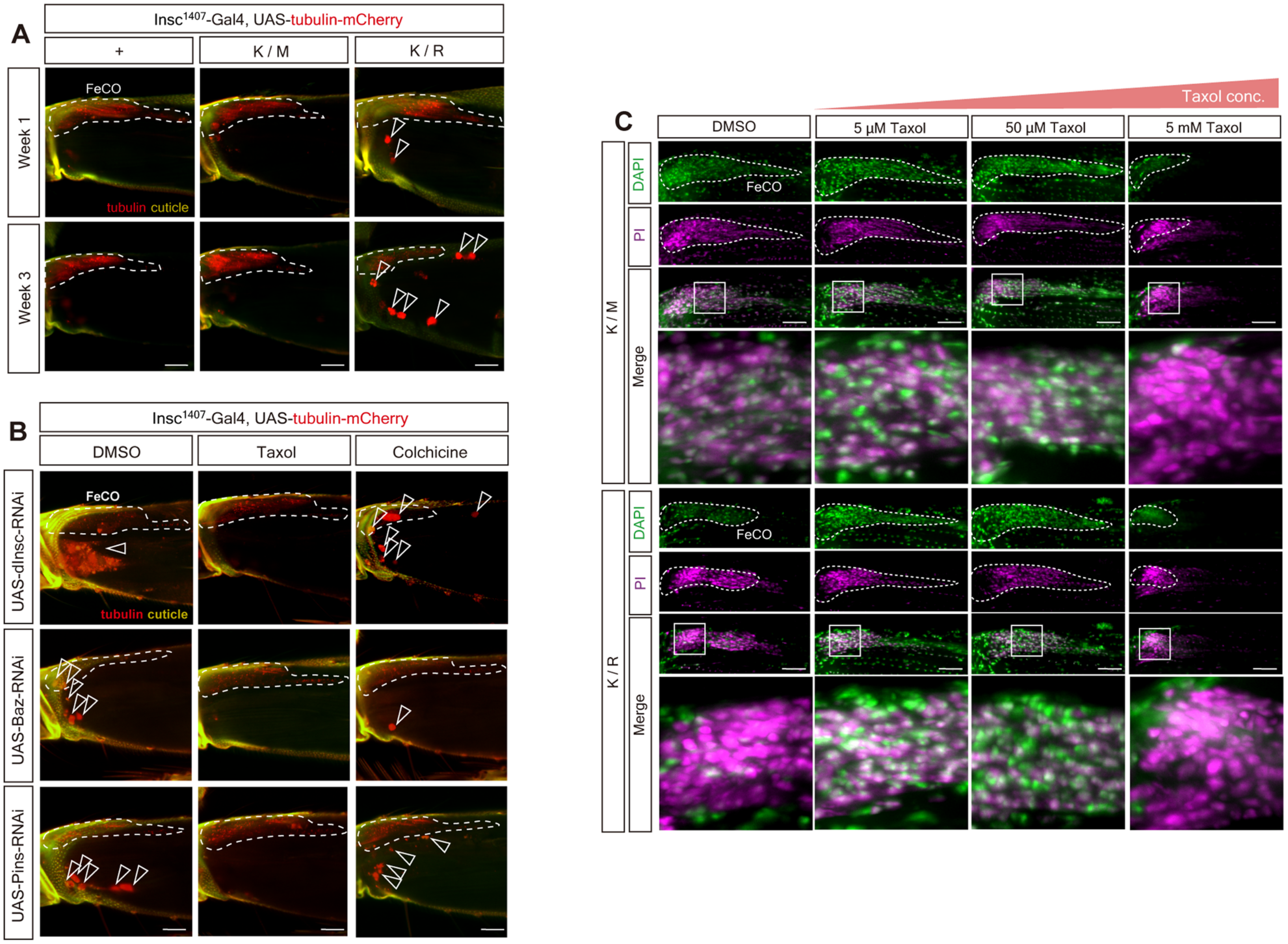


**Figure EV4. Taxol and Colchicine exert opposite effects on tubulin accumulation.**

(**A**) Representative confocal images of tubulin aggregation in *K/M* and *K/R* of 1- and 3-week-old CRISPR-KI flies with *dInsc*$^{1407}$-Gal4>UAS-*tubulin-mCherry*. Arrowheads indicate the aggregative tubulin. Scale bars: 5 µm. (**B**) Representative confocal images of tubulin aggregation (Red indicates *tubulin-mCherry*) in the femur of 3-week-old *dInsc*-RNAi, *Baz*-RNAi and *Pins*-RNAi flies under *dInsc*$^{1407}$-Gal4. Flies were treated with DMSO (vehicle control), Taxol (microtubule-stabilizing agent) and Colchicine (microtubule-destabilizing agent). Yellow indicates the auto-fluorescence of cuticles. The FeCO neurons are encircled by the dashed line. Arrowheads indicate the aggregative tubulin. Scale bars: 5 µm. (**C**) Representative confocal images of FeCO neurons of *K/M* and *K/R* CRISPR-KI flies treated with three different concentrations (5 µM, 50 µM, 5 mM) of Taxol for 7 days, co-stained with PI (magenta) and DAPI (green), and compared with vehicle control (DMSO). The FeCO neurons are encircled by the dashed line. Scale bars: 5 µm.

 © The Author(s)

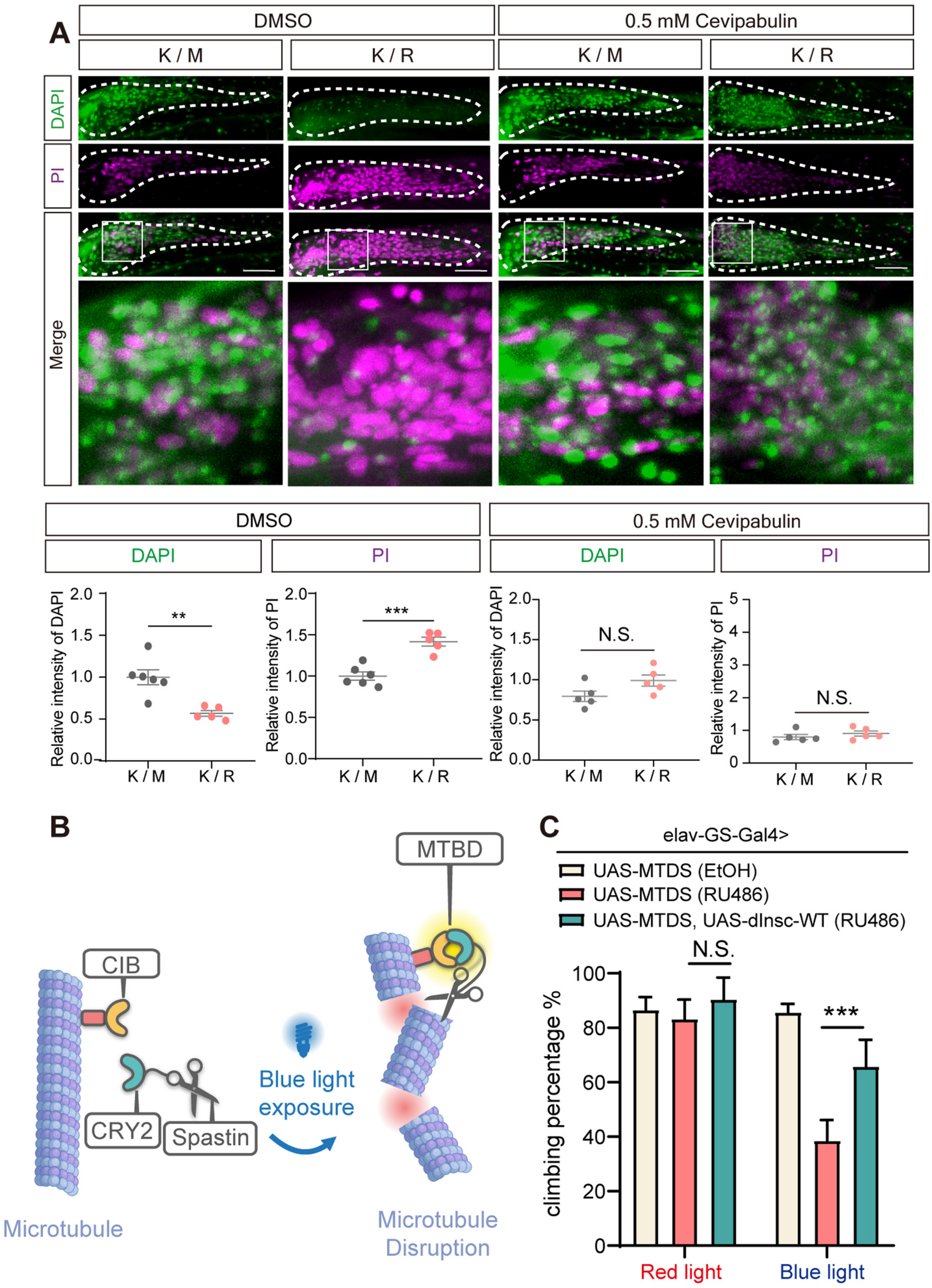
A
DMSO
0.5 mM Cevipabulin
K / M
K / R
DAPI
PI
Merge
Relative intensity of DAPI
Relative intensity of PI
**
***
N.S.
B
MTBD
CIB
CRY2
Spastin
Blue light exposure
Microtubule
Microtubule Disruption
C
elav-GS-Gal4>
UAS-MTDS (EtOH)
UAS-MTDS (RU486)
UAS-MTDS, UAS-dInsc-WT (RU486)
climbing percentage %
Red light
Blue light

**Figure EV5. The destabilization of microtubules caused by M70R mutation can be rescued with both microtubule-stabilizing agents and genetic manipulation.**

(**A**) Representative confocal images of FeCO neurons of *K/M* and *K/R* 3-week flies treated with 0.5 mM of microtubule stabilizer Cevipabulin for 7 days, co-stained with PI (magenta) and DAPI (green), compared with vehicle control (DMSO). The FeCO neurons are encircled by the dashed line. Scale bars: 5 μm. Quantifications are shown in the lower panels. (**B**) The schematic of light-inducible microtubule disassembly system (MTDS) in a fly model. The dimerization of CRY2 and CIB can be induced by blue light. The CRY2 is fused with a microtubule-severing enzyme Spastin, and CIB is fused with microtubule-binding domain (MTBD). Dimerization upon blue light stimuli induces accumulation of Spastins on microtubules, which in turn induces disassembly of microtubules in MTDS-expressing cells. (**C**) Quantification of the climbing activity of Week 1 flies of UAS-*MTDS* (EtOH), UAS-*MTDS* (RU486), and UAS-*MTDS*, UAS-*dInsc-WT* (RU486) under the control of RU486-inducible pan-neuronal driver (*elav-GS*-Gal4) upon 48 h of blue light exposure, comparing with the conditional control (red light); $n = 30$ flies/genotypes from 3 independent fly crosses.

 © The Author(s)
